# Supplementary material for: Wisconsin microbiome study, a cross-sectional investigation of dietary fibre, microbiome composition and antibiotic-resistant organisms: rationale and methods
Source: BMJ Open. 2018 Mar 27;8(3):e019450. doi: 10.1136/bmjopen-2017-019450 (PMC5875638; doi:10.1136/bmjopen-2017-019450)
Supplement: Supplementary file 1 [file bmjopen-2017-019450supp001.pdf]

Multi-Drug Resistant Organisms-related data in the Survey of the Health of Wisconsin instruments

Please refer to the index below to find SHOW instruments that have data related to MDRO. All these instruments can be found in subsequent pages of this document in the order that they are listed here.

SAQ: Self-Administered Questionnaire

CAPI: Computer-Assisted Personal Interview

	Instrument	Description	Details	Mode
1.	ALQ	Alcohol	History of drinking alcoholic beverages	SAQ
2.	ANX	Animal Exposure	Time spent on a farm and with farm animals	SAQ
3.	HHQ	Health History Questionnaire	History of medical conditions, diabetes complications, questions on comorbidities; HHQ194, HHQ580, HHQ581	CAPI
19.	HMH	Household Health History (Microbiome Household)	Healthy history information regarding a participant's household that may influence his/her microbiome	SAQ
20.	HMI	Your Health History (Microbiome Individual)	Health history information that may influence a participant's microbiome	SAQ
24.	HOQ	Housing Characteristics	HOQ250_2: What kind of pets do you keep inside your home now?	SAQ
27.	IUQ	Insurance, Access, and Utilization	Questions on hospital stays; IUQ190 IUQ192, IUQ194	CAPI
40.	LAB	LAB/Sample Collection	LAB300, LAB310, LAB320, LAB330, LAB340, LAB341, LAB342, LAB350, LAB360, LAB370, LAB380, LAB390, LAB400	CAPI
52.	OCQ	Occupation	Work in healthcare setting; OCQ185, OCQ186	CAPI
58.	PHQ	Depression	Two-item depression screener; derived depression diagnosis and score: PHQ2_DX, PHQ2_SCORE	CAPI
60.	PTSD	Post-Traumatic Stress Disorder	Abbreviated PTSD Checklist – Civilian version; derived PTSD diagnosis and score: PTSD6_DX_SCORE, PTSD6_TOTAL_SCORE	CAPI
61.	RXQ	Medications	Number of medications, antibiotics	CAPI
71.	SMQ	Smoking	Smoking history	SAQ

Alcohol Consumption

The next set of questions are about drinking alcoholic beverages. Alcoholic beverages include liquor (such as whiskey or gin), beer, wine, wine coolers, and any other type of drink with alcohol in it.

1. **How many glasses of wine or wine coolers do you usually have per week?**
   This means 5 ounce glasses of wine or 12 ounce bottles of wine cooler (size of a regular can of soda).

   Enter number of glasses: ☐☐ ALQ160_R2 FMT_NUMERIC

2. **How many glasses, bottles, or cans of beer do you usually have per week?**
   This means 12 ounce glasses, bottles, or cans of beer (size of a regular can of soda).

   Enter number of cans, glasses, or bottles: ☐☐ ALQ170_R2 FMT_NUMERIC

3. **How many drinks of hard liquor do you usually have per week?**
   This means one-and-a-half ounce shots.

   ALQ180_R2 FMT_NUMERIC

   Enter number of hard liquor drinks (e.g. 1.5 ounce shots): ☐☐

4. **In the past 12 months, on how many days did you have 5 or more drinks of any alcoholic beverage?**

   If you had 5 or more alcoholic beverages about 1 day per week on average, enter 52. If you usually did this about 2 times per month, enter 24.

   If there was no day in the past 12 months where you had 5 or more drinks, enter 0.

   Enter number of days: ☐☐☐ ALQ130_R3 FMT_NUMERIC

5. **Was there ever a time in your life when you drank 5 or more drinks of any kind of alcoholic beverage almost every day?**

   ALQ120_R3 FMT_YES_NO

   ○ Yes
   ○ No

Animal Exposure

Now we would like to ask you some questions about time you have spent on a farm and with farm animals.

A farm is defined as any establishment from which $1,000 or more of agricultural products were produced or sold, or would normally have been sold, during the year.

	Yes	No	Don't know
**1. Do you live on a farm?**	○	○	○
	ANX010	FMT_YES_NO.	
**2. Do you live on a hobby farm (i.e., a small farm operated for pleasure or supplemental income rather than for primary income)?**	○	○	○
	ANX020	FMT_YES_NO.	
**3. At any time in the <u>past year</u>, did you work, paid or unpaid, on a farm?**	○	○	○
	ANX030	FMT_YES_NO.	

If you filled in only "No" or "Don't know" in response to questions 1 through 3, you are done with the Animal Exposure questionnaire. **Please go to question 1, page 40.** →

If you filled in "Yes" in response to any questions 1 through 3, please continue with question 4.

↓

4. What kinds of animals are kept on the farm on which you live or have worked *(fill in all that apply)*?

- ○ Beef cattle ANX040_a
- ○ Dairy cows ANX040_b
- ○ Horses ANX040_c
- ○ Donkeys ANX040_d
- ○ Llamas ANX040_e
- ○ Chickens ANX040_f
- ○ Ostriches ANX040_g
- ○ Pigs ANX040_h
- ○ Goats for dairy ANX040_i
- ○ Goats for meat ANX040_j
- ○ Sheep ANX040_k
- ○ Other: *Print below.* ANX040_l

ANX040_m $FMT_CHAR.

a through l are FMT_YES_NO.

5. In the <u>past year</u>, were antibiotics given to any of the animals raised on the farm on which you lived or worked?

○ Yes ○ No ○ Don't know ANX050 FMT_YES_NO.

HEALTH HISTORY PART I

HHQ100pre This next questionnaire is about your personal health history.

I will ask you if a health professional ever told you that you have or had certain common health problems in your lifetime and if so, how old you were when these occurred.

For some health problems I will ask you about the treatment you received. If there is any question that you don't know the answer to let me know. If there is any question that makes you uncomfortable you may ask me to skip the question.

INTERVIEWER: HIT ENTER TO CONTINUE

HHQ100 Has a doctor or other health professional ever told you that you had congestive heart failure?

HHQ100 FMT_YES_NO.

<1> YES
<2> NO

<d> DON'T KNOW
<r> REFUSED

HHQ120 (Has a doctor or other health professional ever told you that you had…)

…**angina,** also called **angina pectoris**?

HHQ120 FMT_YES_NO.

<1> YES
<2> NO

<d> DON'T KNOW
<r> REFUSED

HHQ130 (Has a doctor or other health professional ever told you that you had…)

…a **heart attack?**

HHQ130 FMT_YES_NO.

<1> YES
<2> NO **(skip to HHQ140)**

<d> DON'T KNOW **(skip to HHQ140)**
<r> REFUSED **(skip to HHQ140)**

HHQ131 How many heart attacks have you had?

HHQ131 FMT_NUMERIC.

<1-99>

HEALTH HISTORY

<d> DON'T KNOW
<r> REFUSED

HHQ132 How old were you when you were first told you had a heart attack?

HHQ132 FMT_NUMERIC.

<1-130> YEARS

<d> DON'T KNOW
<r> REFUSED

HHQ140 Have you ever had heart surgery?

HHQ140 FMT_YES_NO.

<1> YES
<2> NO **(skip to HHQ150)**

<d> DON'T KNOW **(skip to HHQ150)**
<r> REFUSED **(skip to HHQ150)**

HHQ141 Which of the following types of heart surgery have you had?

(INTERVIEWER: HAND CARD. ENTER ALL THAT APPLY.)

<1> BYPASS SURGERY
<2> ANGIOPLASTY
<3> VALVE SURGERY
<4> PACEMAKER
<5> OTHER (SPECIFY)

<d> DON'T KNOW
<r> REFUSED

**1st RESPONSE**	**HHQ141_A**	**FMT_HHQ141_.**
**2nd RESPONSE**	**HHQ141_B**	**FMT_HHQ141_.**
**3rd RESPONSE**	**HHQ141_C**	**FMT_HHQ141_.**
**4th RESPONSE**	**HHQ141_D**	**FMT_HHQ141_.**
**5th RESPONSE**	**HHQ141_E**	**FMT_HHQ141_.**
**OTHER RESPONSE**	**HHQ141_OTHER**	**$FMT_CHAR.**

HIT 'x' TO EXIT

HHQ150 Has a doctor or other health professional ever told you that you had a transient ischemic attack (TIA)?

HHQ150 FMT_YES_NO.

<1> YES
<2> NO

<d> DON'T KNOW <r> REFUSED

HEALTH HISTORY

HHQ160 (Has a doctor or other health professional ever told you that you had…)

…a **stroke?**

HHQ160 FMT_YES_NO.

<1> YES
<2> NO **(skip to HHQ180)**

<d> DON'T KNOW **(skip to HHQ180)**
<r> REFUSED **(skip to HHQ180)**

HHQ162 How old were you when you were first told that you had a stroke?

HHQ162 FMT_NUMERIC.

<1-130> YEARS

<d> DON'T KNOW
<r> REFUSED

HHQ180 (Has a doctor or other health professional ever told you that you had…)

…**high cholesterol/hyperlipidemia**?

HHQ180 FMT_YES_NO.

<1> YES
<2> NO **(skip to HHQ190)**

<d> DON'T KNOW **(skip to HHQ190)**
<r> REFUSED **(skip to HHQ190)**

HHQ183 How is your high cholesterol/hyperlipidemia currently being treated? List all that apply.

(INTERVIEWER: HAND CARD. ENTER ALL THAT APPLY)

<1> NO TREATMENT
<2> PRESCRIBED MEDICINE
<3> WEIGHT CONTROL/LOSS
<4> EXERCISE
<5> SPECIAL DIET
<6> OTHER (SPECIFY)

<d> DON'T KNOW
<r> REFUSED

**1st RESPONSE**	**HHQ183_A**	**FMT_HHQ183_.**
**2nd RESPONSE**	**HHQ183_B**	**FMT_HHQ183_.**
**3rd RESPONSE**	**HHQ183_C**	**FMT_HHQ183_.**

HEALTH HISTORY

4th RESPONSE HHQ183_D FMT_HHQ183_.
5th RESPONSE HHQ183_E FMT_HHQ183_.
OTHER RESPONSE HHQ183_OTHER $FMT_CHAR.

HHQ190 (Has a doctor or other health professional ever told you that you had…)

…**diabetes**?

HHQ190 FMT_YES_NO.

<1> YES
<2> NO **(skip to HHQ200)**

<d> DON'T KNOW **(skip to HHQ200)**
<r> REFUSED **(skip to HHQ200)**

HHQ191 Which type of diabetes have you had?

(INTERVIEWER: PICK ONLY ONE)

HHQ191 FMT_HHQ191_.

<1> TYPE I
<2> TYPE II
<3> ONLY WHEN PREGNANT
<4> BORDERLINE DIABETES WHICH IS SOMETIMES CALLED PRE-DIABETES

<d> DON'T KNOW
<r> REFUSED

HHQ192 How old were you when you were first told you had diabetes?

HHQ192 FMT_NUMERIC.

<1-130> YEARS

<d> DON'T KNOW
<r> REFUSED

HHQ193 How is your diabetes currently being treated or controlled?

(INTERVIEWER: HAND CARD. ENTER ALL THAT APPLY.)

<1> NO TREATMENT
<2> INSULIN
<3> ORAL ANTI-DIABETICS (PILLS)
<4> WEIGHT CONTROL/LOSS
<5> EXERCISE
<6> SPECIAL DIET
<7> OTHER (SPECIFY)

HEALTH HISTORY

<d> DON'T KNOW
<r> REFUSED

**1st RESPONSE**	**HHQ193_A**	**FMT_HHQ193_.**
**2nd RESPONSE**	**HHQ193_B**	**FMT_HHQ193_.**
**3rd RESPONSE**	**HHQ193_C**	**FMT_HHQ193_.**
**4th RESPONSE**	**HHQ193_D**	**FMT_HHQ193_.**
**5th RESPONSE**	**HHQ193_E**	**FMT_HHQ193_.**
**6th RESPONSE**	**HHQ193_F**	**FMT_HHQ193_.**
**OTHER RESPONSE**	**HHQ193_OTHER**	**$FMT_CHAR.**

HHQ194 Has a doctor or other health care professional ever told you that you had any of the following complications associated with diabetes?

(INTERVIEWER: HAND CARD. ENTER ALL THAT APPLY.) ENTER X TO EXIT.

<1> NEUROPATHY (NERVE DISEASE)
<2> RETINOPATHY (EYE DISEASE)
<3> NEPHROPATHY (KIDNEY DISEASE)
<4> OTHER (SPECIFY)

<d> DON'T KNOW
<r> REFUSED

**1st RESPONSE**	**HHQ194_A**	**FMT_HHQ194_.**
**2nd RESPONSE**	**HHQ194_B**	**FMT_HHQ194_.**
**3rd RESPONSE**	**HHQ194_C**	**FMT_HHQ194_.**
**4th RESPONSE**	**HHQ194_D**	**FMT_HHQ194_.**
**OTHER RESPONSE**	**HHQ194_OTHER**	**$FMT_CHAR.**

HHQ200 (Has a doctor or other health professional ever told you that you had…)

…**high blood pressure/hypertension**?

HHQ200 FMT_YES_NO.

<1> YES
<2> NO **(skip to HHQ210)**

<d> DON'T KNOW **(skip to HHQ210)**
<r> REFUSED **(skip to HHQ210)**

HHQ202 How old were you when you were first told that you had **high blood pressure/hypertension**?

HHQ202 FMT_NUMERIC.

<1-130> YEARS

HEALTH HISTORY

<d> DON'T KNOW
<r> REFUSED

HHQ203 How is your **high blood pressure/hypertension** currently treated? List all that apply.

(INTERVIEWER: HAND CARD. ENTER ALL THAT APPLY.)

<1> NO TREATMENT
<2> PRESCRIBED MEDICINE
<3> WEIGHT CONTROL/LOSS
<4> EXERCISE
<5> SPECIAL DIET
<6> OTHER (SPECIFY)

<d> DON'T KNOW
<r> REFUSED

HIT 'X' TO EXIT

**1st RESPONSE**	**HHQ203_A**	**FMT_HHQ203_.**
**2nd RESPONSE**	**HHQ203_B**	**FMT_HHQ203_.**
**3rd RESPONSE**	**HHQ203_C**	**FMT_HHQ203_.**
**4th RESPONSE**	**HHQ203_D**	**FMT_HHQ203_.**
**5th RESPONSE**	**HHQ203_E**	**FMT_HHQ203_.**
**OTHER RESPONSE**	**HHQ203_OTHER**	**$FMT_CHAR.**

HHQ210 (Has a doctor or other health professional ever told you that you have…)

…**asthma**?

HHQ210 FMT_YES_NO.

<1> YES
<2> NO **(skip to HHQ230r2)**

<d> DON'T KNOW **(skip to HHQ230r2)**
<r> REFUSED **(skip to HHQ230r2)**

HHQ212 How old were you when you were first told you have asthma?

HHQ212 FMT_NUMERIC.

<1-130> YEARS

<d> DON'T KNOW
<r> REFUSED

HHQ214 Do you still have asthma?

HHQ214 FMT_YES_NO.

<1> YES

HEALTH HISTORY

<2> NO

<d> DON'T KNOW
<r> REFUSED

HHQ215 During the last 12 months, have you had an episode of asthma or an asthma attack?

HHQ215 FMT_YES_NO.

<1> YES
<2> NO

<d> DON'T KNOW
<r> REFUSED

HHQ216 During the past 12 months, have you visited an emergency room or urgent care because of your asthma?

HHQ216 FMT_YES_NO.

<1> YES
<2> NO

<d> DON'T KNOW
<r> REFUSED

HHQ217 How is your asthma currently being treated or controlled?

(INTERVIEWER: HAND CARD. ENTER ALL THAT APPLY.)

<1> USE NOTHING/NO TREATMENT
<2> INHALED BRONCHODILATOR
<3> INHALED STERIOD
<4> ORAL MEDICATION
<5> INJECTED MEDICATIONS
<6> CONTROLLING ALLERGIES AND/OR ASTHMA TRIGGERS
<7> WEIGHT CONTROL/LOSS/EXERCISE/SPECIAL DIET
<8> OTHER (SPECIFY)

<d> DON'T KNOW
<r> REFUSED

**1st RESPONSE**	**HHQ217_A**	**FMT_HHQ217_.**
**2nd RESPONSE**	**HHQ217_B**	**FMT_HHQ217_.**
**3rd RESPONSE**	**HHQ217_C**	**FMT_HHQ217_.**
**4th RESPONSE**	**HHQ217_D**	**FMT_HHQ217_.**
**5th RESPONSE**	**HHQ217_E**	**FMT_HHQ217_.**
**6th RESPONSE**	**HHQ217_F**	**FMT_HHQ217_.**
**7th RESPONSE**	**HHQ217_G**	**FMT_HHQ217_.**
**OTHER RESPONSE**	**HHQ217_OTHER**	**$FMT_CHAR.**

HEALTH HISTORY

HHQ218 During the past 30 days, how many days did you take a prescription asthma medication to PREVENT an asthma attack from occurring?

HHQ218 FMT_HHQ218_.

<1> NEVER
<2> 1-14 DAYS
<3> 15-24 DAYS
<4> 25-30 DAYS

<d> DON'T KNOW
<r> REFUSED

HHQ219 During the past 30 days, how many days did you take a prescription asthma medication DURING AN ASTHMA ATTACK to stop it?

HHQ219 FMT_HHQ219_.

<0> NEVER
<1> 1-4 TIMES
<2> 5-14 TIMES
<3> 15-29 TIMES
<4> 30-59 TIMES
<5> 60-99 TIMES
<6> MORE THAN 100 TIMES

<d> DON'T KNOW
<r> REFUSED

HHQ230r2 (Has a doctor or other health professional ever told you that you had…)

…**chronic bronchitis** or **emphysema?**

HHQ230_R2 FMT_YES_NO.

<1> YES
<2> NO

<d> DON'T KNOW
<r> REFUSED

HHQ270 (Has a doctor or other health professional ever told you that you had…)

…**allergies** or **hay fever**?

HHQ270 FMT_YES_NO.

<1> YES
<2> NO **(End of HHQ; GO TO SIQ230)**

<d> DON'T KNOW **(End of HHQ; GO TO SIQ230)**

HEALTH HISTORY

<r> REFUSED **(End of HHQ; GO TO SIC230)**

HHQ271 Which types of allergies have you had?

(INTERVIEWER: HAND CARD. ENTER ALL THAT APPLY)

<1> TREES, GRASSES, PLANTS, POLLEN
<2> MEDICINES
<3> FOODS
<4> CHEMICALS/SCENTS
<5> MOLDS
<6> ANIMALS/DANDER
<7> DUST MITES
<10> STINGING INSECTS
<11> OTHER (SPECIFY)

<d> DON'T KNOW
<r> REFUSED

**1st RESPONSE**	**HHQ271_A**	**FMT_HHQ271_.**
**2nd RESPONSE**	**HHQ271_B**	**FMT_HHQ271_.**
**3rd RESPONSE**	**HHQ271_C**	**FMT_HHQ271_.**
**4th RESPONSE**	**HHQ271_D**	**FMT_HHQ271_.**
**5th RESPONSE**	**HHQ271_E**	**FMT_HHQ271_.**
**6th RESPONSE**	**HHQ271_F**	**FMT_HHQ271_.**
**7th RESPONSE**	**HHQ271_G**	**FMT_HHQ271_.**
**8th RESPONSE**	**HHQ271_H**	**FMT_HHQ271_.**
**9th RESPONSE**	**HHQ271_I**	**FMT_HHQ271_.**
**OTHER RESPONSE**	**HHQ271_OTHER**	**$FMT_CHAR.**

HHQ276 Do you still have allergies or hay fever?

HHQ276 FMT_YES_NO.

<1> YES
<2> NO

<d> DON'T KNOW
<r> REFUSED

(SIQ230 and SIQ231 for MEN >=40 YEARS OLD. If <40, skip to SDQ010 below)

SIQ230 Have you ever had a prostate blood test, PSA test, and/or a rectal exam?

SIQ230 FMT_YES_NO.

<1> YES
<2> NO **(Skip to SDQ010)**

<d> DON'T KNOW **(Skip to SDQ010)**

<r> REFUSED **(Skip to SDQ010)**

SIQ231 Within how many years?

SIQ231 FMT_NUMERIC.

INTERVIEWER: IF LESS THAN 1 YEAR, ENTER 1 FOR YEARS

<1-130> YEARS

<d> DON'T KNOW
<r> REFUSED

SDQ010 At the **present time**, would you say your eyesight, with glasses or contact lenses, if you wear them, is excellent, good, fair, poor or very poor?

SDQ010 FMT_EGFPVP.

<1> EXCELLENT
<2> GOOD
<3> FAIR
<4> POOR
<5> VERY POOR

<d> DON'T KNOW
<r> REFUSED

SDQ270 Overall, how would you describe the condition of your teeth?

Would you say excellent, very good, good, fair or poor?

SDQ270 FMT_EVGGFP.

<1> EXCELLENT
<2> VERY GOOD
<3> GOOD
<4> FAIR
<5> POOR

<d> DON'T KNOW
<r> REFUSED

SDQ340 Have you ever been diagnosed by a dentist as having gum disease?

SDQ340 FMT_YES_NO.

<1> YES
<2> NO

<d> DON'T KNOW
<r> REFUSED

HEALTH HISTORY

SDQ360 During the past 12 months, was there a time when you needed dental care but did not get it at that time?

SDQ360 FMT_YES_NO.

<1> YES
<2> NO **(Skip to HHQ400)**

<d> DON'T KNOW **(Skip to HHQ400)**
<r> REFUSED **(Skip to HHQ400)**

SDQ361 What were the reasons that you could not get the dental care you needed?

(INTERVIEWER: ENTER ALL THAT APPLY. HAND CARD)

<1> COULD NOT AFFORD THE COST
<2> DID NOT WANT TO SPEND THE MONEY
<3> DO NOT HAVE INSURANCE
<4> INSURANCE DID NOT COVER RECOMMENDED PROCEDURES

<5> INSURANCE ONLY COVERS A PORTION OF THE COST
<6> DENTAL OFFICE IS TOO FAR AWAY
<7> DENTAL OFFICE IS NOT OPEN AT CONVENIENT TIMES

<8> ANOTHER DENTIST RECOMMENDED NOT DOING IT
<9> AFRAID, OR DO NOT LIKE DENTISTS
<10> UNABLE TO TAKE TIME OFF FROM WORK
<11> TOO BUSY
<12> I DID NOT THINK ANYTHING SERIOUS WAS WRONG--EXPECTED DENTAL PROBLEMS TO GO AWAY
<13> THE DENTIST WOULD NOT ACCEPT MY INSURANCE
<14> OTHER (SPECIFY)

<d> DON'T KNOW
<r> REFUSED

HIT 'x' TO EXIT

**1*st* response**	**SDQ361_A**	**FMT_SDQ361_.**
**2*nd* response**	**SDQ361_B**	**FMT_SDQ361_.**
**3*rd* response**	**SDQ361_C**	**FMT_SDQ361_.**
**4*th* response**	**SDQ361_D**	**FMT_SDQ361_.**
**5*th* response**	**SDQ361_E**	**FMT_SDQ361_.**
**6*th* response**	**SDQ361_F**	**FMT_SDQ361_.**
**7*th* response**	**SDQ361_G**	**FMT_SDQ361_.**
**8*th* response**	**SDQ361_H**	**FMT_SDQ361_.**
**9*th* response**	**SDQ361_I**	**FMT_SDQ361_.**
**10*th* response**	**SDQ361_J**	**FMT_SDQ361_.**
**11*th* response**	**SDQ361_K**	**FMT_SDQ361_.**
**12*th* response**	**SDQ361_L**	**FMT_SDQ361_.**
**13*th* response**	**SDQ361_M**	**FMT_SDQ361_.**
**14*th* response**	**SDQ361_N**	**FMT_SDQ361_.**

HEALTH HISTORY

Other response SDQ361_OTHER $FMT_CHAR.

HHQ400 Has a doctor or other health professional ever told you that you were overweight?

HHQ400 FMT_YES_NO.

<1> YES
<2> NO **(Skip to HHQ480)**

<d> DON'T KNOW **(Skip to HHQ480)**
<r> REFUSED **(Skip to HHQ480)**

HHQ412 How old were you when you were first told you were overweight?

HHQ412 FMT_NUMERIC.

<0-130> YEARS OLD

<d> DON'T KNOW
<r> REFUSED

HHQ480 Has a doctor or other health professional ever told you that you had cancer?

HHQ480 FMT_YES_NO.

<1> YES
<2> NO **(Skip to HHQ500int)**

<d> DON'T KNOW **(Skip to HHQ500int)**
<r> REFUSED **(Skip to HHQ500int)**

HHQ481 Which types of cancer on this card have you had?

(INTERVIEWER: ENTER ALL THAT APPLY, ENTER 'x' WHEN DONE)

<10> BLADDER
<11> BLOOD
<12> BONE
<13> BRAIN
<14> BREAST
<15> CERVIX/CERVICAL
<16> COLON
<17> ESOPHAGUS
<18> GALLBLADDER
<19> KIDNEY
<20> LARYNX/WINDPIPE
<21> LEUKEMIA
<22> LIVER
<23> LUNG
<24> LYMPHOMA/ HODGKINS DISEASE
<25> MELANOMA
<27> NERVOUS SYSTEM
<28> OVARY/OVARIAN
<29> PANCREAS/PANCREATIC
<30> PROSTATE
<31> RECTUM/RECTAL
<32> SKIN (NON MELANOMA)
<33> SKIN (UNKNOWN)
<34> SOFT TISSUE (MUSCLE/FAT)
<35> STOMACH
<36> TESTES/TESTICULAR
<37> THYROID
<38> UTERUS/UTERINE
<39> OTHER

<66> MORE THAN 3

<d> DON'T KNOW

HEALTH HISTORY

<26> MOUTH/TONGUE/LIP <r> REFUSED

HHQ481_A FMT_HHQ481_.
HHQ481_B FMT_HHQ481_.
HHQ481_C FMT_HHQ481_.
HHQ481_D FMT_HHQ481_.
HHQ481_OTHER $FMT_CHAR.

HHQ500int Now we will ask you questions about certain illnesses that have occurred in your biological or blood relatives--- your grandparents, parents, aunts, uncles, brothers, sisters, and children. Please do not include half or step sisters or brothers, cousins, nieces, nephews, or grandchildren. Please include both living and deceased relatives.

INTERVIEWER: HIT ENTER TO CONTINUE

HHQ510_R2 Were any of your biological or blood relatives ever told by a doctor or other health professional that they had diabetes?

HHQ510 FMT_YES_NO.

<1> YES
<2> NO

<d> DON'T KNOW
<r> REFUSED

HHQ520_R2 (Were any of your biological or blood relatives ever told by a doctor or other health professional that they had…)

…Alzheimer's disease or dementia?

HHQ520 FMT_YES_NO.

<1> YES
<2> NO

<d> DON'T KNOW
<r> REFUSED

HHQ530_R2 (Were any of your biological or blood relatives ever told by a doctor or other health professional that they had…)

…asthma?

HHQ530 FMT_YES_NO.

<1> YES
<2> NO

HEALTH HISTORY

<d> DON'T KNOW
<r> REFUSED

HHQ550_R2 (Were any of your biological or blood relatives ever told by a doctor or other health professional that they had…)

…high blood pressure or hypertension?

HHQ550 FMT_YES_NO.

<1> YES
<2> NO

<d> DON'T KNOW
<r> REFUSED

HHQ570_R3 (Were any of your biological or blood relatives ever told by a doctor or other health professional that they had…)

… a heart attack or a stroke?

HHQ570_R2 FMT_YES_NO.

<1> YES
<2> NO **(Skip to HHQ580new)**

<d> DON'T KNOW **(Skip to HHQ580new)**
<r> REFUSED **(Skip to HHQ580new)**

HHQ572_R3 What was the youngest age at which any biological or blood relative was first diagnosed with heart attack or a stroke?

HHQ572_R2 FMT_NUMERIC.

<0-130> YEARS OLD

<d> DON'T KNOW
<r> REFUSED

HHQ580new Has a doctor or other health professional ever told you that you had any of the following?
(INTERVIEWER: HAND CARD. ENTER ALL THAT APPLY, ENTER 'xx' FOR NONE OR FOR NO FURTHER DIAGNOSES)

<a> ALCOHOL ABUSE
<b> ALZHEIMER'S DISEASE
<c> ANEMIA
<d> ANXIETY
<e> AUTISM SPECTRUM DISORDER

<t> KIDNEY STONES
<u> LEARNING DISABILITY
<ua> LIVER DISEASE
<v> LYME DISEASE
<w> MIGRAINE HEADACHE
<x> MILD COGNITIVE IMPAIRMENT

HEALTH HISTORY

<f> CELIAC DISEASE
<g> CHLAMYDIA
<h> CROHN'S DISEASE
<i> CHRONIC KIDNEY DISEASE
<j> DEPRESSION
<k> DRUG ABUSE
<l> ECZEMA/DERMATITIS
<m> EPILEPSY
<n> GONERRHEA
POX
<oa> HEPATITIS A
<ob> HEPATITIS B
<oc> HEPATITIS C
<p> HERPES TYPE 1/COLD SORES
<q> HIV INFECTION/AIDS
<r> HUMAN PAPILLOMA VIRUS (HPV)
<s> IRRITABLE BOWEL SYNDROME

<y> MULTIPLE SCLEROSIS
<z> OSTEOARTHRITIS
<aa> OSTEOPOROSIS
<bb> PARKINSON'S DISEASE
<cc> POST TRAUMATIC STRESS DISORDER (PTSD)
<dd> PSORIASIS
<ee> REFLUX/GERD
<ff> RHEUMATOID ARTHRITIS
<gg> SHINGLES OR CHICKEN
<hh> SICKLE CELL DISEASE
<ii> STOMACH OR INTESTINAL ULCER
<jj> SYPHILIS
<kk> TUBERCULOSIS
<ll> URINARY INCONTINENCE
<mm> URINARY TRACT INFECTION

<xd> DON'T KNOW <xr> REFUSED
<xx> NO FURTHER DIAGNOSES

HHQ580_A $FMT_DISEASE.
HHQ580_B $FMT_DISEASE.
HHQ580_C $FMT_DISEASE.
HHQ580_D $FMT_ DISEASE.
HHQ580_E $FMT_ DISEASE.
HHQ580_F $FMT_ DISEASE.
HHQ580_G $FMT_ DISEASE.
HHQ580_H $FMT_ DISEASE.
HHQ580_I $FMT_ DISEASE.
HHQ580_J $FMT_ DISEASE.
HHQ580_K $FMT_ DISEASE.
HHQ580_L $FMT_ DISEASE.

HHQ581 Has a doctor or other health professional ever told you that you had any of these following conditions?

(INTERVIEWER: HAND CARD. ENTER ALL THAT APPLY, ENTER 'x' FOR NONE OR FOR NO FURTHER DIAGNOSES)

<1> CONNECTIVE TISSUE DISEASE
<2> PERIPHERAL VASCULAR DISEASE
<3> HEMIPLEGIA
<4> SKIN OR SOFT TISSUE INFECTION

<d> DON'T KNOW <r> REFUSED
<x> NO FURTHER DIAGNOSES

HHQ581_A FMT_HHQ581_R2_.

HEALTH HISTORY

HHQ581_B FMT_HHQ581_R2_.
HHQ581_C FMT_HHQ581_R2_.
HHQ581_D FMT_HHQ581_R2_.

CGQ_intro There are situations in which people provide regular **unpaid care or assistance** to a family member **including children** or a friend who has **a long-term illness or a disability**.

INTERVIEWER: HIT ENTER TO CONTINUE

CGQ010 **In the past 12 months**, did you provide any such care or assistance to a family member or friend living with you or living elsewhere?

CGQ010 FMT_YES_NO.

<1> YES
<2> NO **(Skip to RXQ032pre)**

<d> DON'T KNOW **(Go to CGQ020)**
<r> REFUSED **(Skip to RXQ032pre)**

CGQ020 Are you currently giving unpaid help to a family member or friend?

CGQ020 FMT_YES_NO.

<1> YES
<2> NO

<d> DON'T KNOW
<r> REFUSED

Household Health History

These questions ask about the health history of others, rather than yourself.

1. **In the past 12 months, have you visited someone staying in a healthcare facility (e.g., hospital, nursing home, inpatient rehabilitation facility)?**

   ○ Yes
   ○ No → **Go to question 4** HMH010 FMT_YES_NO.
   ○ Don't know → **Go to question 4**

2. **In the past 12 months, did you provide help in caring for the person(s) while they were staying in the healthcare facility?** ***By help in caring, we mean having physical or hands-on contact with the person.***

   ○ Yes ○ No ○ Don't know HMH020 FMT_YES_NO.

3. **In the past 12 months, about how many total days did you make a visit to someone who was staying in a healthcare facility (e.g., hospital, nursing home, inpatient rehabilitation facility)?**

   [|] total number of days HMH030 FMT_NUMERIC.

4. **Has anyone in your household had an infection with a drug-resistant germ?**

   ○ Yes ○ No ○ Don't know HMH040 FMT_YES_NO.

5. **Has anyone in your household had an infection from a hospital or healthcare setting?**

   ○ Yes → *Specify the infection(s) that they had below.* HMH050 FMT_YES_NO.
   [ ] HMH055 $FMT_CHAR.
   ○ No
   ○ Don't know

6. **Have you ever visited a person who was placed in isolation while in a hospital, nursing home, or inpatient rehabilitation facility?** ***That is, you were required to wear at least a pair of gloves and a gown before seeing them.***

   ○ Yes ○ No ○ Don't know HMH060 FMT_YES_NO.

Your Health History

This next set of questions is about your health history.

1. At the time of your birth, were you delivered by Caesarean section?

◯ Yes<br>◯ No<br>◯ Don't know	HMI010	FMT_YES_NO.

2a. Were you breastfed as an infant?

◯ Yes<br>◯ No → **Go to question 3**<br>◯ Don't know	HMI020	FMT_YES_NO.

2b. For how long were you breastfed?

☐☐ months<br>◯ Don't know	HMI025	FMT_NUMERIC.

3. Have you ever had surgery on your digestive system (e.g. esophagus, stomach, liver, appendix, small and large intestines, gall bladder, and/or pancreas)?

◯ Yes<br>◯ No<br>◯ Don't know	HMI030	FMT_YES_NO.

4. In the past 12 months, have you had any of the following medical devices?
Fill in all that apply.

◯ Urinary catheter	HMI040_a	FMT_YES_NO.
◯ Vascular catheter	HMI040_b	FMT_YES_NO.
◯ Feeding tube	HMI040_c	FMT_YES_NO.
◯ Rectal tube	HMI040_d	FMT_YES_NO.
◯ Don't know	HMI040_e	FMT_YES_NO.

5. In the past 12 months, have you had dialysis treatment?

- ◯ Yes
- ◯ No — HMI050 FMT_YES_NO.
- ◯ Don't know

6a. Have you ever been a patient in a nursing home or inpatient rehabilitation facility?

- ◯ Yes
- ◯ No → **Go to question 7a, page 11** — HMI060 FMT_YES_NO.
- ◯ Don't know

6b. How many times were you a patient in a nursing home or inpatient rehabilitation facility?

[ ][ ] times — HMI062 FMT_NUMERIC.

◯ Don't know

6c. When was your most recent stay in a nursing home or inpatient facility? *Please tell us what month and year this visit began.*

[ ][ ] month — HMI065_m FMT_NUMERIC.

[ ][ ][ ][ ] year — HMI065_y FMT_NUMERIC.

6d. What was the approximate length of stay?

[ ][ ] days — HMI068 FMT_NUMERIC.

7a. In the past year, have you taken an antibiotic *(a drug used to treat an infection)*?

○ Yes
○ No → **Go to question 8a** HMI070 FMT_YES_NO.
○ Don't know → **Go to question 8a**

7b. In the table below, please list the name(s) of the antibiotics, the illness or condition for which you took them, and the length of time you took them. *If you were prescribed the same antibiotic more than once in the past year, list it multiple times.*

Name the antibiotic you took in the last year below:	*The reason (illness or condition) for taking the medication:*	*For how many days did you take this antibiotic?*
1. HMI071_a $FMT_CHAR.	HMI071_b $FMT_CHAR.	HMI071_c $FMT_CHAR.
2. HMI072_a $FMT_CHAR.	HMI072_b $FMT_CHAR.	HMI072_c $FMT_CHAR.
3. HMI073_a $FMT_CHAR.	HMI073_b $FMT_CHAR.	HMI073_c $FMT_CHAR.
4. HMI074_a $FMT_CHAR.	HMI074_b $FMT_CHAR.	HMI074_c $FMT_CHAR.
5. HMI075_a $FMT_CHAR.	HMI075_b $FMT_CHAR.	HMI075_c $FMT_CHAR.

☐ **If you have had more than five antibiotic prescriptions in the past year, please check this box.** HMI076 FMT_YES_NO.

8a. Are you currently using probiotic supplements? *Specifically, we are referring to pills containing healthy bacteria.*

○ Yes
○ No → **Go to question 9, page 12** HMI080 FMT_YES_NO.
○ Don't know → **Go to question 9, page 12**

8b. When was the last time you took the probiotic supplement?

○ Today or ☐☐ number of days ago HMI085 FMT_NUMERIC.

9. In the past 12 months, have you taken a proton pump inhibitor? ***Proton pump inhibitors are drugs that suppress the production of acid in your stomach.*** **Some examples of trade (generic) names are: Aciphex® (rabeprazole), Protonix® (pantoprazole), Nexium® (esomeprazole), Prevacid® (lansoprazole), Kapidex® (dexlansoprazole), Zegerid® (omeprazole/sodium bicarbonate), Prilosec® (omeprazole), Dexilant® (dexlansoprazole).**

- ◯ Yes
- ◯ No — HMI090 FMT_YES_NO.
- ◯ Don't know

10. Has a doctor or other health care provider ever told you that you had an infection with a drug-resistant germ? ***A germ is resistant when one or more drugs ordinarily used to treat an infection with that germ cannot kill it.***

- ◯ Yes → *Specify the infection(s) you had below.* — HMI100 FMT_YES_NO.

HMI105 $FMT_CHAR.

- ◯ No
- ◯ Don't know

11. Has a doctor or other health care provider ever told you that you got an infection from a hospital or health care setting?

- ◯ Yes → *Specify the infection(s) you had below.* — HMI110 FMT_YES_NO.

HMI115 $FMT_CHAR.

- ◯ No
- ◯ Don't know

12. Have you ever been put in isolation as a patient in a hospital, nursing home, or inpatient rehabilitation facility? ***That is, visitors were required to wear at least a pair of gloves and gown before seeing you.***

- ◯ Yes
- ◯ No — HMI120 FMT_YES_NO.
- ◯ Don't know

Housing Characteristics

The next section asks questions about your home and your exposure to certain hazards in the home.

1. When was this home or building originally built?

HOQ040_R3 ○ Before 1900 HOQ040_R3 FMT_HOQ040_R2_.
○ 1901 to 1950
○ 1951 to 1978
○ 1979 to 1990
○ 1991 and after
○ Don't know

2. How long have you lived at this address?

HOQ060_R3 ○ 0-1 years HOQ060_R3 FMT_HOQ060_R2_.
○ 1-3 years
○ 3-10 years
○ >10 years

3. What kind of pets do you keep inside your home now? *Fill in all that apply.*

HOQ250_R2
○ None
○ Cat
○ Dog
○ Bird
○ Hamster, mice, guinea pig, gerbils
○ Reptile
○ Fish
○ Other

HOQ250_R2_A FMT_YES_NO.
HOQ250_R2_B FMT_YES_NO.
HOQ250_R2_C FMT_YES_NO.
HOQ250_R2_D FMT_YES_NO.
HOQ250_R2_E FMT_YES_NO.
HOQ250_R2_F FMT_YES_NO.
HOQ250_R2_G FMT_YES_NO.
HOQ250_R2_H FMT_YES_NO.

4. Do you have a basement in this home?

HOQ066_R2 ○ Yes
○ No HOQ066_R2 FMT_YES_NO.

5. **What is the main type of heating system in this home?**

HOQ067_R2
- ◯ Steam radiators
- ◯ Hot water radiators / heaters
- ◯ Forced air system using gas or fuel oil
- ◯ Active solar
- ◯ Wood burning stoves
- ◯ Portable electric space heaters
- ◯ Other types of space heaters
- ◯ Other: *Print below.*
- ◯ Don't know

HOQ067_R2 FMT_HOQ067_R2_.
HOQ067_OTHER_R2 $FMT_CHAR.

6. **Is your home connected to a private well or to a community water supply?**

HOQ070_R2
- ◯ Private well
- ◯ Community water supply → **Go to question 8**
- ◯ Don't know → **Go to question 8**

HOQ070_R2 FMT_HOQ070_R2_.

7. **Approximately how deep is your well?**

HOQ075_R2 FMT_HOQ075_.

HOQ075_R2
- ◯ < 50 feet
- ◯ 50-99 feet
- ◯ 100-149 feet
- ◯ > 150 feet
- ◯ Don't know

8. **Do you use a home water filter/treatment system in the home for drinking water?**

HOQ080_R2
- ◯ Yes
- ◯ No → **Go to question 10, page 38**
- ◯ Don't know → **Go to question 10, page 38**

HOQ080_R2 FMT_YES_NO.

9. **Are any of these water filter/t eatment systems in your home?** ***Fill in all that apply.***

HOQ083_R2
- ◯ Brita or other pitcher with water filter
- ◯ Ceramic or charcoal filter
- ◯ Water softener
- ◯ Aerator
- ◯ Reverse osmosis
- ◯ None of these are in our home
- ◯ Other: *Print below.*
- ◯ Don't know

HOQ083_R2_A FMT_YES_NO.
HOQ083_R2_B FMT_YES_NO.
HOQ083_R2_C FMT_YES_NO.
HOQ083_R2_D FMT_YES_NO.
HOQ083_R2_E FMT_YES_NO.
HOQ083_R2_F FMT_YES_NO.
HOQ083_R2_G FMT_YES_NO.
HOQ083_R2_OTHER $FMT_CHAR.

This next section is about chemicals, such as insecticides and weed killers, that may have been used outside or inside your home. These products could have been used by you, another household member, a landlord, or a company.

10. During the last 12 months, how often were weed killers or insecticides used on the foundation, yard/lawn, flowers, vegetables, o fruit trees *outside* your house?

HOQ261_R2

- ○ 0 times
- ○ 1 time
- ○ 2-3 times
- ○ 4-10 times
- ○ > 10 times
- ○ Don't know

HOQ261_R2 FMT_HOQ261_R2_.

11. During the last 12 months, how often were chemicals such as pesticides used inside your home to kill or control insects or other pests?

HOQ266_R2

- ○ 0 times → **Go to question 1, page 39**
- ○ 1 time
- ○ 2-3 times
- ○ 4-10 times
- ○ > 10 times
- ○ Don't know

HOQ266_R2 FMT_HOQ261_R2_.

12. Which rooms in your home were treated with this product? *Fill in all that apply.*

HOQ270_R2

- ○ Kitchen
- ○ Bathroom
- ○ Living room or family room
- ○ Bedroom
- ○ Laundry room
- ○ Basement
- ○ Other: *Print below.*
- ○ Don't know

HOQ270_R2_A	FMT_YES_NO.
HOQ270_R2_B	FMT_YES_NO.
HOQ270_R2_C	FMT_YES_NO.
HOQ270_R2_D	FMT_YES_NO.
HOQ270_R2_E	FMT_YES_NO.
HOQ270_R2_F	FMT_YES_NO.
HOQ270_R2_G	FMT_YES_NO.
HOQ270_R2_OTHER	$FMT_CHAR.

INSURANCE, ACCESS, UTILIZATION (IUQ)

IUQ010pre The next questions are about health insurance and your use of the health care system.

INTERVIEWER: HIT ENTER TO CONTINUE

IUQ010 During the last 12 months, how many months did you have health insurance?

(INTERVIEWER: IF NO INSURANCE DURING 12 PRECEDING MONTHS, ENTER "0".)

IUQ010 FMT_NUMERIC.

<0-12> **MONTHS** **(If 0, skip to IUQ012. If 12, skip to IUQ020_R2. Otherwise, go to IUQ015.)**

<d> DON'T KNOW **(Skip to IUQ015)**
<r> REFUSED **(Skip to IUQ015)**

IUQ012 If you wanted to, could you be covered by health insurance through a job or through a household family member's job? That is, do you or a household family member parent or spouse have an employer that offers health insurance?

IUQ012 FMT_IUQ012_.

<1> EMPLOYER (EITHER YOURS OR FAMILY MEMBER'S) OFFERS HEALTH INSURANCE **(SKIP TO IUQ014)**
<2> EMPLOYER (EITHER YOURS OR FAMILY MEMBER'S) **DOES NOT** OFFER HEALTH INSURANCE **(GO TO IUQ013)**

<d> DON'T KNOW **(GO TO IUQ013)**
<r> REFUSED **(GO TO IUQ013)**

IUQ013 Did you consider purchasing individual health insurance through the new health care program, known as the "Affordable Care Act" or "Obamacare," that allows many individuals to purchase subsidized insurance through the Marketplace?

Would you say yes, but it was too expensive because you did not qualify for a subsidy, yes, but it was too expensive even with a subsidy, you were not eligible to purchase through the marketplace, or you did not consider purchasing coverage through the Marketplace?

IUQ013 FMT_IUQ013_.

(INTERVIEWER: HAND CARD)

<1> YES, BUT IT WAS TOO EXPENSIVE BECAUSE I DID NOT QUALIFY FOR A SUBSIDY **(SKIP TO IUQ100)**
<2> YES, BUT IT WAS TOO EXPENSIVE EVEN WITH A SUBSIDY (**SKIP TO IUQ100)**
<3> I WAS NOT ELIGIBLE TO PURCHASE THROUGH THE MARKETPLACE **(SKIP TO IUQ100)**
<4> I DID NOT CONSIDER PURCHASING COVERAGE THROUGH THE MARKETPLACE **(SKIP TO IUQ100)**

INSURANCE, ACCESS, UTILIZATION (IUQ)

<d> DON'T KNOW (**SKIP TO IUQ100)**
<r> REFUSED **(SKIP TO IUQ100)**

IUQ014 Why don't you have health insurance coverage from that employer? You are not eligible for the health insurance plan, it is too expensive and you cannot afford the premiums, or you do not think it is worth it?

IUQ014 FMT_IUQ014_.

<1> I AM NOT ELIGIBLE FOR THE HEALTH INSURANCE PLAN (**SKIP TO IUQ100)**
<2> IT IS TOO EXPENSIVE – CANNOT AFFORD THE PREMIUMS **(SKIP TO IUQ100)**
<3> I DO NOT THINK IT IS WORTH IT **(Skip to IUQ100)**

<d> DON'T KNOW (**SKIP TO IUQ100)**
<r> REFUSED (**SKIP TO IUQ100)**

IUQ015 FMT_YES_NO.

IUQ015 Do you currently have health insurance?

<1> YES **(GO TO IUQ020_R2)**
<2> NO **(GO TO IUQ020_R2)**

<d> DON'T KNOW **(SKIP TO IUQ100)**
<r> REFUSED **(SKIP TO IUQ100)**

IUQ020_R2 What kinds of health insurance or health care coverage do you have now, or did you have during the last 12 months? In answering this question, please EXCLUDE plans that pay for only one type of service, such as nursing home care, accidents, family planning, or dental care, and plans that only provide extra cash when hospitalized.

(**INTERVIEWER: HAND CARD. ENTER ALL THAT APPLY.)**

<1> EMPLOYER OR UNION SPONSORED PLAN **[GOTO IUQ021]**
<2> PRIVATE INDIVIDUALLY PURCHASED HEALTH PLAN **[GOTO IUQ025]**
<3> MEDICARE, FOR PEOPLE 65 OR OLDER OR PEOPLE WITIH CERTAIN DISABILITIES **[GOTO IUQ030]**

<5> MEDICAID, MEDICAL ASSISTANCE, MA, BADGER CARE, BADGER CARE PLUS **[GOTO IUQ030]**

<8> INDIAN HEALTH SERVICE **[GOTO IUQ030]**
<9> MILITARY CARE (TRICARE/VA/CHAMP-VA) **[GOTO IUQ030]**
<10> OTHER PLAN (SPECIFY) **[GOTO IUQ025]**

<d> DON'T KNOW **[goto IUQ025]**
<r> REFUSED **[goto IUQ025]**

INSURANCE, ACCESS, UTILIZATION (IUQ)

HIT “x” TO EXIT

**IUQ020_R2_A**	**FMT_IUQ020_R2_.**
**IUQ020_R2_B**	**FMT_IUQ020_R2_.**
**IUQ020_R2_C**	**FMT_IUQ020_R2_.**
**IUQ020_R2_D**	**FMT_IUQ020_R2_.**
**IUQ020_R2_E**	**FMT_IUQ020_R2_.**
**IUQ020_R2_F**	**FMT_IUQ020_R2_.**
**IUQ020_R2_G**	**FMT_IUQ020_R2_.**
**IUQ020_R2_H**	**FMT_IUQ020_R2_.**
**IUQ020_R2_I**	**FMT_IUQ020_R2_.**
**IUQ020_R2_J**	**FMT_IUQ020_R2_.**
**IUQ020_R2_OTHER**	**FMT_CHAR R2_.**

IUQ021 Do you get the Employer or Union Sponsored Plan coverage through your own job or from a family member’s insurance plan?

**IUQ021**	**FMT_IUQ021_.**

<1> YOUR OWN JOB OR EMPLOYER

<2> A FAMILY MEMBER’S JOB OR EMPLOYER
<3> OTHER

<d> DON’T KNOW
<r> REFUSED

[ALL RESPONSES AT IUQ021 GO TO IUQ023]

IUQ023 Was your job based coverage purchased through the SHOP, Small Business Health Options Program?

**IUQ023**	**FMT_YES_NO.**

<1> YES **(IF IUQ020_R2 = 2, 10, d, or r GO TO IUQ025, ELSE SKIP TO IUQ026)**

<2> NO **(IF IUQ020_R2 = 2, 10, d, or r GO TO IUQ025, ELSE SKIP TO IUQ030)**

<d> DON’T KNOW **(IF IUQ020_R2 = 2, 10, d, or r GO TO IUQ025, ELSE SKIP TO IUQ030)**
<r> REFUSED **(IF IUQ020_R2 = 2, 10, d, or r GO TO IUQ025, ELSE SKIP TO IUQ030)**

IUQ025 The next questions ask about the new health care program, known as the Affordable Care Act or “Obamacare.” As you may know, the health care law creates health insurance exchanges or marketplaces where people can shop for insurance on

INSURANCE, ACCESS, UTILIZATION (IUQ)

Healthcare.gov. Some people can get financial help in the form of a tax credit from the federal government to buy a health insurance policy through these marketplaces.

Did you or a family member buy your private health insurance plan from this Marketplace, healthcare.gov?

IUQ025 FMT_YES_NO.

<1> YES **(GO TO IUQ026)**

<2> NO **(SKIP TO IUQ030)**

<d> DON'T KNOW **(GO TO IUQ026)**
<r> REFUSED **(GO TO IUQ026)**

IUQ026 Do you know what kind of health plan you have? Bronze, silver, gold, platinum, catastrophic or are you not sure?

IUQ026 FMT_IUQ026_.

<1> BRONZE
<2> SILVER
<3> GOLD
<4> PLATINUM
<5> CATASTROPHIC
<6> NOT SURE

<d> DON'T KNOW
<r> REFUSED

IUQ027 Did you or your family member get a federal tax credit or subsidy to help with or reduce the costs of buying your health insurance plan?

<1> YES
<2> NO

IUQ027 FMT_YES_NO.

<d> DON'T KNOW
<r> REFUSED

IUQ030 Does your health insurance plan, including any supplemental coverage you might have, cover all of the costs, some of the costs, or none of the costs associated with prescription medications?

IUQ030 FMT_ALL_SOME_NONE.

<1> ALL
<2> SOME
<3> NONE **(SKIP TO IUQ040)**

<d> DON'T KNOW
<r> REFUSED

INSURANCE, ACCESS, UTILIZATION (IUQ)

IUQ035 Is this prescription drug coverage through your regular plan or through a supplemental insurance program for prescription drug coverage?

(INTERVIEWER: ENTER ALL THAT APPLY)

<1> REGULAR PLAN
<2> SUPPLEMENTAL, MEDICARE PART D
<3> SUPPLEMENTAL, WISCONSIN SENIOR CARE
<4> SUPPLEMENTAL, OTHER (SPECIFY)

<d> DON'T KNOW
<r> REFUSED

HIT 'x' TO EXIT

**IUQ035_A**	**FMT_IUQ035_.**
**IUQ035_B**	**FMT_IUQ035_.**
**IUQ035_C**	**FMT_IUQ035_.**
**IUQ035_D**	**FMT_IUQ035_.**
**IUQ035_OTHER**	**FMT_CHAR.**

IUQ040 Does your insurance plan cover all of the costs, some of the costs, or none of the costs associated with preventive dental services including oral exam, cleaning, sealant, etc.?

**IUQ040**	**FMT_ALL_SOME_NONE.**

<1> ALL **(SKIP TO IUQ050)**
<2> SOME **(GO TO IUQ044)**
<3> NONE **(GO TO IUQ044)**

<d> DON'T KNOW
<r> REFUSED

IUQ044 If your health insurance plan did not cover all of the costs, do you have a separate dental plan that pays for preventive services?

<1> YES
<2> NO

**IUQ044**	**FMT_YES_NO.**

<d> DON'T KNOW
<r> REFUSED

IUQ050 Does your health insurance plan cover all of the costs, some of the costs, or none of the costs associated with other preventive services for adults, like checkups, immunizations, and screenings?

<1> ALL
<2> SOME
<3> NONE

**IUQ050**	**FMT_ALL_SOME_NONE.**

<d> DON'T KNOW
<r> REFUSED

INSURANCE, ACCESS, UTILIZATION (IUQ)

IUQ070 Does your plan require you to sign up with a certain primary care doctor, group of doctors, or a certain clinic that you must go to for all of your routine care?

<1> YES
<2> NO

<d> DON'T KNOW
<r> REFUSED

IUQ070 FMT_YES_NO.

IUQ100 In the last 12 months, have you used the internet to seek information or advice on your health, or that of your family?

<1> YES
<2> NO

<d> DON'T KNOW
<r> REFUSED

IUQ100 FMT_YES_NO.

IUQ105 In the last 12 months, have you telephoned a health care professional to discuss a health problem or question related to yourself or your family?

<1> YES
<2> NO

<d> DON'T KNOW
<r> REFUSED

IUQ105 FMT_YES_NO.

IUQ110 In the last 12 months, have you emailed a health care professional to discuss a health problem or question related to yourself or your family?

<1> YES
<2> NO

<d> DON'T KNOW
<r> REFUSED

IUQ110 FMT_YES_NO.

IUQ115 How often do you need to have someone help you when you read instructions, pamphlets, or other written material from your doctor or pharmacy?

Would you say never, rarely, sometimes, often or always?

<1> NEVER
<2> RARELY
<3> SOMETIMES
<4> OFTEN
<5> ALWAYS

<d> DON'T KNOW
<r> REFUSED

IUQ115 FMT_FREQ_IUQ115_.

INSURANCE, ACCESS, UTILIZATION (IUQ)

IUQ120 Do you have a usual place where you go when you feel sick or need advice about your health?

(INTERVIEWER: HAND CARD)

**IUQ120**	**FMT_IUQ120_.**
**IUQ120_OTHER**	**FMT_CHAR.**

<1> YES, I USUALLY GO TO A HOSPITAL EMERGENCY ROOM
<2> YES, I USUALLY GO TO A HOSPITAL OUTPATIENT DEPARTMENT
<3> YES, I USUALLY GO TO A CLINIC OR DOCTOR'S OFFICE
<4> YES, I USUALLY GO TO A COMMUNITY HEALTH CENTER
<5> YES, I USUALLY GO TO SOME OTHER PLACE (SPECIFY)
<6> NO, I DON'T HAVE A USUAL PLACE OF CARE **(Skip to IUQ140)**

<d> DON'T KNOW
<r> REFUSED

IUQ125 What is the name of the health facility you usually go to when you feel sick or need advice about your health and on what streets in what town/city is this facility located?

NAME: ____________________________

STREET ON WHICH THE FACILITY IS LOCATED: ______________________

NEAREST INTERSECTING OR CROSS STREET: ______________________

TOWN/CITY: ______________________________

<d> DON'T KNOW
<r> REFUSED

**IUQ125_A**	**FMT_CHAR.**
**IUQ125_C**	**FMT_CHAR.**
**IUQ125_D**	**FMT_CHAR.**
**IUQ125_B**	**FMT_CHAR.**

IUQ128 When you go to this health facility, do you usually see a general doctor, a specialist doctor, a nurse practitioner or physician assistant or someone else?

<1> GENERAL DOCTOR
<2> SPECIALIST DOCTOR
<3> NURSE PRACTITIONER/PHYSICIAN ASSISTANT
<4> SOMEONE ELSE

<d> DON'T KNOW
<r> REFUSED

**IUQ128**	**FMT_IUQ128_.**

IUQ130 When you go to this health facility ,do you usually see the same health care provider?

<1> YES
<2> NO **(SKIP TO IUQ140)**

**IUQ130**	**FMT_YES_NO.**

<d> DON'T KNOW
<r> REFUSED

INSURANCE, ACCESS, UTILIZATION (IUQ)

IUQ137 What is the specialty of the health care provider you usually see?

**IUQ137**	**FMT_IUQ137_.**
**IUQ137_OTHER**	**FMT_CHAR.**

<1> INTERNAL MEDICINE
.
<2> FAMILY PRACTICE

<3> OBSTETRICS/GYNECOLOGY
<4> OTHER SPECIALIST (SPECIFY)

<d> DON'T KNOW
<r> REFUSED

IUQ140 Sometimes people take fewer medicines than their health care provider prescribed, or they don't have their prescription filled right away.

At any time during the last 12 months, have you taken less medicine than your doctor prescribed or not had your prescription filled **because of the cost**?

<1> YES
<2> NO

IUQ140 FMT_YES_NO.

<d> DON'T KNOW
<r> REFUSED

IUQ170 In the last 12 months, how many different **times** have you seen a mental health professional such as a psychologist, psychiatrist, counselor, or psychiatric nurse about a personal problem or a problem with alcohol or drugs?

<0-76> TIMES DURING PREVIOUS YEAR

IUQ170 FMT_NUMERIC.

<d> DON'T KNOW
<r> REFUSED

IUQ180 In the last 12 months, how many different **times** did you go to a hospital emergency room for medical treatment for yourself?

<0-76> TIMES DURING PREVIOUS YEAR

IUQ180 FMT_NUMERIC.

<d> DON'T KNOW
<r> REFUSED

IUQ190 In the last 12 months, how many different **times** were you a patient in a hospital for at least one night or longer?

<0> NO TIMES **(skip to IUQ220)**
<1-76> TIMES DURING PREVIOUS YEAR

IUQ190 FMT_NUMERIC.

<d> DON'T KNOW **(skip to IUQ220)**

INSURANCE, ACCESS, UTILIZATION (IUQ)

<r> REFUSED **(skip to IUQ220)**

IUQ192 For each time you were in the hospital in the last 12 months, for how many days did you stay in the hospital? Begin with your most recent hospital stay and tell us about your stay for up to 5 hospital stays.

{stay 1} <1-30> DURATION

UNITS
{unit 1} <1> DAYS
<2> MONTHS

**IUQ192_S1**	**FMT_NUMCAT.**
**IUQ192_S2**	**FMT_NUMCAT.**
**IUQ192_S3**	**FMT_NUMCAT.**
**IUQ192_S4**	**FMT_NUMCAT.**
**IUQ192_S5**	**FMT_NUMCAT.**

<d> DON'T KNOW
<r> REFUSED

**IUQ192_U1**	**FMT_DAYS_MONTHS.**
**IUQ192_U2**	**FMT_DAYS_MONTHS.**
**IUQ192_U3**	**FMT_DAYS_MONTHS.**
**IUQ192_U4**	**FMT_DAYS_MONTHS.**
**IUQ192_U5**	**FMT_DAYS_MONTHS.**

STAY #1 @s1 DURATION @u1 UNIT (Will display number given in IUQ190)
STAY #2 @s2 DURATION @u2 UNIT
STAY #3 @s3 DURATION @u3 UNIT
STAY #4 @s4 DURATION @u4 UNIT
STAY #5 @s5 DURATION @u5 UNIT

IUQ194 For any of your hospitalizations in the last 12 months, how many times [endif]were you in an intensive care unit?

<0> NO
<1> YES <1-[fill IUQ190]> TIMES **[maximum is number of hospitalizations in IUQ190**]

**IUQ194**	**FMT_NUMCAT.**

<d> DON'T KNOW
<r> REFUSED

IUQ200 How would you rate the quality of the care you received when you were most recently a patient in a hospital for at least one night or longer **during the last year?**

Would you say it was excellent, very good, good, fair or poor?

<1> EXCELLENT
<2> VERY GOOD
<3> GOOD
<4> FAIR
<5> POOR

**IUQ200**	**FMT_EVGGFP.**

<d> DON'T KNOW
<r> REFUSED

IUQ220 How long has it been since you last saw a doctor or health care provider for a

INSURANCE, ACCESS, UTILIZATION (IUQ)

routine physical exam, check-up or screening procedure?

<0> NEVER **(SKIP TO IUQ260pre)**
<1-76> ENTER NUMBER **(Go to IUQ230)**

**IUQ220_N**	**FMT_NUMCAT.**
**IUQ220_U**	**FMT_FREQ.**

<d> DON'T KNOW **(Go to IUQ225)**
<r> REFUSED **(Skip to IUQ230)**

<1> DAYS **(Skip to IUQ230)**
<2> WEEKS **(Skip to IUQ230)**
<3> MONTHS **(Skip to IUQ230)**
<4> YEARS **(If more than 1 year, skip to IUQ260pre. Otherwise skip to IUQ230)**

IUQ225 Has it been never, 6 months or less, more than 6 months but no more than 1 year ago, more than 1 year ago but no more than 3 years ago or more than 3 years ago?

**IUQ225**	**FMT_IUQ225_255_.**

<1> NEVER **(Skip to IUQ260pre)**
<2> 6 MONTHS OR LESS **(Go to IUQ230)**
<3> MORE THAN 6 MONTHS BUT NO MORE THAN 1 YEAR AGO **(GO TO IUQ230)**
<4> MORE THAN 1 YEAR BUT NO MORE THAN 3 YEARS AGO **(Skip to IUQ260pre)**
<5> MORE THAN 3 YEARS AGO **(Skip to IUQ260pre)**

<d> DON'T KNOW **(Skip to IUQ260pre)**
<r> REFUSED **(Skip to IUQ260pre)**

IUQ230 How would you rate the quality of the care you received when you last saw a doctor or health care provider for a routine physical exam, check-up, or screening procedure during the last year?

**IUQ230**	**FMT_EVGGFP.**

Would you say it was excellent, very good, good, fair or poor?

<1> EXCELLENT
<2> VERY GOOD
<3> GOOD
<4> FAIR
<5> POOR

<d> DON'T KNOW
<r> REFUSED

IUQ260pre The next questions are about your **overall** level of satisfaction with quality and access to health care.

INSURANCE, ACCESS, UTILIZATION (IUQ)

INTERVIEWER: HIT ENTER TO CONTINUE

IUQ260_R2 Sometimes people have problems getting health care when they need it. During the last 12 months, was there any time that you felt that you needed medical care or surgery but did not get it?

IUQ260_R2 FMT_YES_NO.

<1> YES **(Go to IUQ265)**
<2> NO **(Skip to IUQ270)**

<d> DON'T KNOW **(Skip to IUQ270)**
<r> REFUSED **(Skip to IUQ270)**

IUQ265 What was the main reason you didn't get the health care you needed?

IUQ265 FMT_IUQ265_.
IUQ265_OTHER $FMT_CHAR.

(INTERVIEWER: HAND CARD)

<1> I couldn't afford health care
<2> My insurance company wouldn't approve, cover or pay for care
<3> My insurance company required a referral but I couldn't get one
<4> The doctor (or clinic) refused to accept my insurance plan
<5> Medical care was too far away
<6> It was too expensive to get to health care
<7> I couldn't get there when the doctor's office was open
<8> It took too long to get an appointment
<9> I couldn't get through on the telephone to make an appointment
<10> The waiting list was too long
<11> Other (Specify)

<d> DON'T KNOW
<r> REFUSED

IUQ270 In the past 12 months, did you experience **delay** in obtaining any type of health care?

IUQ270 FMT_YES_NO.

<1> YES
<2> NO **(Skip to IUQ280)**

<d> DON'T KNOW **(Skip to IUQ280)**
<r> REFUSED **(Skip to IUQ280)**

IUQ275 What was the main reason for the difficulty or delay in obtaining health care?

(INTERVIEWER: HAND CARD)

IUQ275 FMT_IUQ275_.

<1> I couldn't afford health care
<2> My insurance company wouldn't approve, cover or pay for care
<3> My insurance company required a referral but I couldn't get one
<4> The doctor refused to accept my insurance plan

INSURANCE, ACCESS, UTILIZATION (IUQ)

<5> Medical care was too far away
<6> It was too expensive to get to health care
<7> I couldn’t get there when the doctor’s office was open
<8> It took too long to get an appointment
<9> I couldn’t get through on the telephone to make an appointment
<10> The waiting list was too long
<11> Other (Specify)

<d> DON’T KNOW
<r> REFUSED

IUQ280 Overall, how would you rate the quality of the health care you received during the last 12 months?

IUQ280 FMT_EVGGFP.

(INTERVIEWER: HAND CARD)

Would you say it was excellent, very good, good, fair, poor or you did not receive any care?

<1> EXCELLENT
<2> VERY GOOD
<3> GOOD
<4> FAIR
<5> POOR
<6> NOT APPLICABLE (DID NOT RECEIVE ANY CARE)

<d> DON’T KNOW
<r> REFUSED

IUQ290 Overall, how satisfied were you with **the way** health care services were provided during the last 12 months?

IUQ290 FMT_SATIS_5CAT.

(INTERVIEWER: HAND CARD)

Were you very satisfied, somewhat satisfied, neither satisfied nor dissatisfied, somewhat dissatisfied, very dissatisfied or you did not receive any care?

<1> VERY SATISFIED
<2> SOMEWHAT SATISFIED
<3> NEITHER SATISFIED NOR DISSATISFIED
<4> SOMEWHAT DISSATISFIED
<5> VERY DISSATISFIED
<6> NOT APPLICABLE (DID NOT RECEIVE ANY CARE)

<d> DON’T KNOW
<r> REFUSED

IUQ300 I want to hear you read as many words as you can from this list. Begin with the first word and read aloud. When you come to a word you cannot read, do the best you can or say, 'blank' and go onto the next word.

INSURANCE, ACCESS, UTILIZATION (IUQ)

IUQ300 FMT_NUMERIC.

INTERVIEWER: HAND CARD AND RECORD THE NUMBER OF CORRECT PRONUNCIATIONS

IF THE RESPONDENT TAKES MORE THAN FIVE SECONDS ON A WORD, POINT TO THE NEXT WORD, IF NECESSARY, TO MOVE THE SUBJECT ALONG. IF THE SUBJECT BEGINS TO MISS EVERY WORD, HAVE HIM OR HER PRONOUNCE ONLY KNOWN WORDS.

Menopause

Antibiotics

Exercise

Jaundice

Rectal

Anemia

Behavior

<0-7> CORRECT PRONUNCIATIONS

<d> DON'T KNOW
<r> REFUSED

Laboratory Tests (LAB)

[CONSENT QUESTIONS HAVE BEEN REMOVED. SEE CONS2 CODEBOOK.]

LABdate Date of Lab: **LABDATE DATE.**
<d> Don't know <r> Refused

Date:
MM/DD/YYYY

[PARTICIPATION VARIABLES ARE NOT INCLUDED IN CODEBOOKS]

PART010 Was this an in-home appointment?

<1> YES **(Skip to LAB020)**
<2> NO **(go to PART020)**

PART020 Is the participant a confirmed driver who needs to be reimbursed for mileage to the appointment?
<1> YES **(go to PART030)**
<2> NO **(Skip to PART040)**

PART030 What was the round trip mileage to the sample collection site?
<0-99> ENTER NUMBER OF MILES

PART040 Does the participant need to be compensated for childcare?
<1> ONE CHILD ($12)
<2> TWO CHILDREN ($15)
<3> THREE OR MORE CHILDREN ($19.50)
<4> NO COMPENSATION REQUIRED

PART050 Does the participant need to be reimbursed for expenses like a bus, parking, or a taxi?
<1> YES, FOR A BUS **(go to PART060)**
<2> YES, FOR PARKING **(go to PART060)**
<3> YES, FOR A TAXI **(go to PART060)**
<4> NO **(Skip to LAB010)**

PART060 What was the total of the other expenses?
<0.00-99.00> ENTER NUMBER OF DOLLARS

LAB020 Phlebotomist Numbers:

<0-9999>

(ENTER ALL THAT APPLY, ENTER 'x' WHEN DONE)

LAB030 Processor Numbers:
<0-9999>

(ENTER ALL THAT APPLY, ENTER 'x' WHEN DONE)

Laboratory Tests (LAB)

LAB300 Did the participant attempt to donate any samples for the Wisconsin Microbiome Study?

<1> YES **(go to LAB310)**
<2> NO **(Skip to LAB410)** **LAB300 FMT_YES_NO.**

LAB310 Type: **Nasal Swab**
Status: **LAB310_S FMT_LAB_STATUS.**
<d> DONE
<f> FAILED **[goto LAB320]**
<r> REFUSED **[goto LAB320]**
<na> NOT ATTEMPTED **[goto LAB320]**

Collection Time: (Military time) **LAB310_C HHMM5.**
HH:MM

Refrigeration Time: (Military time) **LAB310_R HHMM5.**
HH:MM

LAB320 Type: **Oropharyngeal Swab**
Status: **LAB320_S FMT_LAB_STATUS.**
<d> DONE
<f> FAILED **[goto LAB330]**
<r> REFUSED **[goto LAB330]**
<na> NOT ATTEMPTED **[goto LAB330]**

Collection Time: (Military time) **LAB320_C HHMM5.**
HH:MM

Refrigeration Time: (Military time) **LAB320_R HHMM5.**
HH:MM

LAB330 Type: **Saliva Cup**
Status: **LAB330_S FMT_LAB_STATUS.**
<d> DONE
<f> FAILED **[goto LAB340]**
<r> REFUSED **[goto LAB340]**
<na> NOT ATTEMPTED **[goto LAB340]**

Collection Time: (Military time) **LAB330_C HHMM5.**
HH:MM

Refrigeration Time: (Military time) **LAB330_R HHMM5.**
HH:MM

LAB340 Type: **Axilla/Groin Swab**
Status: **LAB340_S FMT_LAB_STATUS.**

Laboratory Tests (LAB)

<d> DONE
<f> FAILED [**goto LAB341]**
<r> REFUSED **[goto LAB341]**
<na> NOT ATTEMPTED **[goto LAB341]**

Collection Time: (Military time) **LAB340_C HHMM5.**
HH:MM

Refrigeration Time: (Military time) **LAB340_R HHMM5.**
HH:MM

LAB341 What under-arm product do you most often use?

<1> DEODORANT ALONE
<2> ANTIPERSPIRANT ALONE
<3> DEODORANT/ANTIPERSPIRANT COMBINATION
<4> I USE NO PRODUCTS UNDER MY ARM [GO TO LAB350]
<5> OTHER PRODUCT, PLEASE SPECIFY

____

<d> DON'T KNOW <r> REFUSED **LAB341 FMT_LAB341_.**

LAB342 How often do you use the product above?

<1> LESS THAN ONCE A MONTH
<2> A FEW TIMES A MONTH
<3> A FEW TIMES A WEEK
<4> EVERY DAY

<d> DON'T KNOW <r> REFUSED **LAB342 FMT_LAB342_.**

LAB350 **Stool Sample**
Status: **LAB350 FMT_LAB_STATUS.**
<d> DONE
<i> INCOMPLETE – GAVE SHIPPER **(skip to LAB410)**
<r> REFUSED **(skip to LAB410)**

LAB360 When was the stool sample produced?
<d> Don't know <r> Refused
Date: **LAB360_DATE DATE.**
MM/DD/YYYY

Time: (Military time) **LAB360_TIME HHMM5.**
HH:MM

LAB370 When was the stool sample first refrigerated?
<d> Don't know <r> Refused

Laboratory Tests (LAB)

Date: **LAB370_DATE DATE.**
MM/DD/YYYY

Time: (Military time) **LAB370_TIME HHMM5.**
HH:MM

LAB380 When was sample received by phlebotomist:

Date: **LAB380_DATE DATE.**
MM/DD/YYYY

Time: (Military time) **LAB380_TIME HHMM5.**
HH:MM

LAB390 Time placed in refrigerator or cooler: (Military time)
HH:MM **LAB390_TIME HHMM5.**

LAB400 When were samples removed from cooler and mailed?

Date: **LAB400_DATE DATE.**
MM/DD/YYYY

Time: (Military time) **LAB400_TIME HHMM5.**
HH:MM

LAB410 Did the subject attempt to donate any samples for SHOW Core?
<1> YES **(Go to LAB010)**
<2> NO **(Skip to 250)** **LAB410 FMT_YES_NO.**

LAB010 Check for any of the follow that restricted your choice of arm/vein:
(CHECK ALL THAT APPLY, ENTER ‘x’ WHEN DONE)

<1>	Mastectomy	**LAB010_A**	**FMT_LAB010_.**
<2>	Hematoma	**LAB010_B**	**FMT_LAB010_.**
<3>	Burns, Scars, Tattoos	**LAB010_C**	**FMT_LAB010_.**
<4>	Damaged veins	**LAB010_D**	**FMT_LAB010_.**
<5>	Shunt, Fistula or Graft	**LAB010_E**	**FMT_LAB010_.**
<6>	Recent IV	**LAB010_F**	**FMT_LAB010_.**
<7>	Caste	**LAB010_G**	**FMT_LAB010_.**
<8>	Edema	**LAB010_H**	**FMT_LAB010_.**
<9>	Obesity	**LAB010_I**	**FMT_LAB010_.**
<10>	Skin sores	**LAB010_J**	**FMT_LAB010_.**

LAB040
When was the last time you ate or drank anything other than plain water?

[INTERVIEWER: THIS QUESTION ELICITS THE LAST TIME THE SP ATE OR DRANK ANYTHING AND DETERMINES FASTING TIME. SPS ARE ALLOWED

Laboratory Tests (LAB)

TO CONSUME DIET SODA, BLACK COFFEE, OR TEA WITH ARTIFICIAL SWEETENERS LIKE SWEET'N LOW, NUTRASWEET, EQUAL, OR SPLENDA SINCE THESE HAVE NO EFFECT ON STUDY ANALYTES.

PARTICIPANTS ARE NOT ALLOWED TO HAVE CONSUMED: COFEE OR TEA WITH CREAM OR SUGAR, FLAVORED WATER, ALCOHOL, GUM, MINTS, LOZENGES, COUGH DROPS, COLD REMEDIES, ANTACIDS, ANTI-DIARRHEALS, LAXATIVES, OR DIETARY SUPPLEMENTS SUCH AS VITAMINS AND MINERALS.]

Last Ate:
<d> Don't know <r> Refused

Date: **LAB040_DATE DATE**
MM/DD/YYYY

Time: (Military time) **LAB040_TIME HHMM5.**
HH:MM

LAB050 1ST Draw Attempt TIME
(Military time)
<d> Don't know <r> Refused

HH:MM

Draw Time: **LAB050 HHMM5.**
HH:MM

LAB060 Type: **5mL SST Gold top for ML**

ML Label**:** [ALLOW 9 CHARACTERS]

Status: **LAB060 FMT_LAB_STATUS.**

<d> DONE
<f> FAILED
<p> PARTIAL
<r> REFUSED
<na> NOT ATTEMPTED

QC: <1> Checked <2> Unchecked

Comments?
<1> Enter Comments
<2> No Comments

Laboratory Tests (LAB)

LAB070 Type: **10mL Redtop for Repository 1**

SPID Label **(SCAN):**

Status: **LAB070 FMT_LAB_STATUS.**
<d> DONE
<f> FAILED
<p> PARTIAL
<r> REFUSED
<na> NOT ATTEMPTED

QC: <1> Checked <2> Unchecked

Comments?
<1> Enter Comments
<2> No Comments

LAB080 Type: **10mL Redtop for Repository 2**

SPID Label **(SCAN): [FILL FROM LAB070]**

Status: **LAB080 FMT_LAB_STATUS.**
<d> DONE
<f> FAILED
<p> PARTIAL
<r> REFUSED
<na> NOT ATTEMPTED

QC: <1> Checked <2> Unchecked

Comments?
<1> Enter Comments
<2> No Comments

LAB090 Type: **10 mL Lavender 1**

SPID Label **(SCAN): [FILL FROM LAB070]**

Status: **LAB090 FMT_LAB_STATUS.**
<d> DONE
<f> FAILED
<p> PARTIAL
<r> REFUSED
<na> NOT ATTEMPTED

NO DNA: <1> Checked <2> Unchecked

QC: <1> Checked <2> Unchecked

Laboratory Tests (LAB)

Comments?
<1> Enter Comments
<2> No Comments

LAB100 Type: **10 mL Lavender 2**

SPID Label **(SCAN): [FILL FROM LAB070]**

Status: **LAB100 FMT_LAB_STATUS.**
<d> DONE
<f> FAILED
<p> PARTIAL
<r> REFUSED
<na> NOT ATTEMPTED

NO DNA: <1> Checked <2> Unchecked

QC: <1> Checked <2> Unchecked

Comments?
<1> Enter Comments
<2> No Comments

LAB110 Type: **3 mL Lavender for ML 1**

ML Label:

Status: **LAB110 FMT_LAB_STATUS.**
<d> DONE
<f> FAILED
<p> PARTIAL
<r> REFUSED
<na> NOT ATTEMPTED

QC: <1> Checked <2> Unchecked

Comments?
<1> Enter Comments
<2> No Comments

LAB120 Type: **3 mL Lavender for ML 2**

ML Label:

Laboratory Tests (LAB)

Status: **LAB120 FMT_LAB_STATUS.**
<d> DONE
<f> FAILED
<p> PARTIAL
<r> REFUSED
<na> NOT ATTEMPTED

QC: <1> Checked <2> Unchecked

Comments?
<1> Enter Comments
<2> No Comments

LAB125 Type: **2.5 mL PaxGene Redtop for Biorepository**

SPID Label: **[FILL FROM LAB070]**

Status: **LAB125 FMT_LAB_STATUS.**
<d> DONE
<f> FAILED
<r> REFUSED
<na> NOT ATTEMPTED

QC: <1> Checked <2> Unchecked

Comments?
<1> Enter Comments
<2> No Comments

LAB130 **Number of Attempted Sticks**
Status: **LAB130 FMT_NUMERIC.**
<1-10> ATTEMPTS
<f> FAILED
<r> REFUSED
<na> NOT ATTEMPTED

Comments?
<1> Enter Comments
<2> No Comments

LAB140 END DRAW TIME **LAB140 HHMM5.**
(Military time)

<d> Don’t know <r> Refused

HH:MM

Laboratory Tests (LAB)

LAB150 Plasma Centrifuge Start Time: **LAB150 HHMM5.**
(Military time)

<d> Don't know <r> Refused

Blood: HH:MM

LAB155 Serum Centrifuge Start Time: **LAB155 HHMM5.**
(Military time)

<d> Don't know <r> Refused

HH:MM

LAB160 Number of plasma vials: **LAB160 FMT_NUMERIC.**

<0-30> CRYOVIALS

<d> Don't know <r> Refused

LAB170 Number of serum vials: **LAB170 FMT_NUMERIC.**

<0-30> CRYOVIALS

<d> Don't know <r> Refused

LAB180 Plasma Freezer Entry Time **LAB180 HHMM5.**
(Military time)

<d> Don't know <r> Refused

HH:MM

LAB185 Serum Freezer Entry Time **LAB185 HHMM5.**

(Military time)

<d> Don't know <r> Refused

HH:MM

LAB200 Type: **Urine Sample**

SPID Label: **[FILL FROM LAB070]**

Laboratory Tests (LAB)

Status: **LAB200 FMT_LAB_STATUS.**
<1> DONE

<7> REFUSED (**goto LAB235)**
<8> FAILED (**goto LAB235)**
<9> NOT ATTEMPTED (**goto LAB235)**

Comments?
<1> Enter Comments
<2> No Comments

LAB190 Urine Sample Collection time **LAB190 HHMM5.**
(Military time)

<d> Don't know <r> Refused

HH:MM

Urine Sample Centrifuge Time **LAB191 HHMM5.**
HH:MM

LAB210 Urine Sample **LAB210 FMT_NUMERIC.**

<0-50> mL of urine centrifuged

<d> Don't know
<r> Refused

LAB220 Number of urine vials: **LAB220 FMT_NUMERIC.**

<0-30> CRYOVIALS

<d> Don't know <r> Refused

QC: <1> Checked <2> Unchecked

LAB230 Urine Freezer Time **LAB230 HHMM5.**
(Military time)

HH:MM

<d> Don't know <r> Refused

LAB235 BLOOD SPOTS
SPID Label:

Laboratory Tests (LAB)

Blood Spot Status: **LAB235 FMT_LAB_STATUS.**
<d> DONE <r> REFUSED (Skips to LAB240)
<f> FAILED (skips toLAB240) <na> NOT ATTEMPTED-BLOOD DRAW COMPLETED (Skip to LAB240)

Blood Spot Collection Time **LAB235_TIM1 HHMM5.**
(Military time)

HH:MM

Blood Spot Freezer Time **LAB235_TIM2 HHMM5.**
(Military time)

HH:MM

Number of spots completed on card
<0-8> **LAB235_NBS FMT_NUMERIC.**
<d> Don't know <r> Refused

LAB236 Blood Spots Comments?
<1> Enter Comments
<2> No Comments

LAB240 Saliva Sample
SPID Label: **[FILL FROM LAB070]**

Saliva Status: **LAB240_A FMT_LAB_STATUS.**
<d> DONE <r> REFUSED
<f> FAILED <na> NOT ATTEMPTED-BLOOD DRAW COMPLETED

Saliva Collection Time **LAB240 HHMM5.**
(Military time)

HH:MM

Saliva Freezer Time **LAB241 HHMM5.**
(Military time)

HH:MM

QC: <1> Checked <2> Unchecked

LAB250 Problems/Comments **LAB250 $FMT_CHAR.**
<1> Enter Comments

Laboratory Tests (LAB)

<2> No Comments

PART160 Was the 24 Hour Dietary Recall completed?

<1> YES
<2> NO, REFUSED
<3> PENDING PARTICIPATION

OCCUPATION (OCQ)

OCQ1pre In this part of the survey I will ask you questions about your work experience

INTERVIEWER: HIT ENTER TO CONTINUE

OCQ100 Which of the following were you doing **last week?**

(INTERVIEWER: HAND CARD.)

<1> Working at a job or business **(Skip to OCQ125)**
<2> With a job or business but not at work (for example, on vacation or sick) **(Skip to OCQ120)**
<3> Not working but looking for work **(Go on to OCQ110)**
<4> Not working at a job or business and not looking for a job **(Go on to OCQ110)**

<d> DON'T KNOW **(Skip to PAQ200pre)**
<r> REFUSED **(Skip to PAQ200pre)**

**OCQ100**	**FMT_OCQ100_.**

OCQ110 What is the main reason you are not in the paid workforce?

<1> TAKING CARE OF HOUSE OR FAMILY
<2> GOING TO SCHOOL
<3> RETIRED
<4> UNABLE TO WORK FOR HEALTH REASONS
<5> ON LAYOFF
<6> DISABLED
<7> OTHER (INTERVIEWER: SPECIFY RESPONSE FOLLOWED BY //)

<d> DON'T KNOW
<r> REFUSED

**OCQ110**	**FMT_OCQ110.**
**OCQ110_OTHER**	**$FMT_CHAR.**

OCQ115 How long have you been out of the paid workforce?

<1-76> ENTER NUMBER OF WEEKS, MONTHS, OR YEARS
<666> NEVER WORKED FOR PAY

<d> DON'T KNOW
<r> REFUSED

**OCQ115_N**	**FMT_NUMCAT.**

<2> WEEKS
<3> MONTHS

OCCUPATION (OCQ)

<4> YEARS

**OCQ115_U**	**FMT_FREQ.**

[SKIP TO PAQ200pre AFTER OCQ115]

OCQ120 What is the **main** reason you did not work **last week**?

<1> VACATION / LEAVE
<2> SICK OR TAKING CARE OF SICK FAMILY MEMBER
<3> CHILDCARE PROBLEMS
<4> EMPLOYER DID NOT SCHEDULE ME
<4> OTHER (INTERVIEWER: SPECIFY RESPONSE FOLLOWE D BY //)

<d> DON'T KNOW
<r> REFUSED

**OCQ120**	**FMT_OCQ120_.**
**OCQ120_OTHER**	**$FMT_CHAR.**

OCQ122 Do you **usually** work 35 hours or more per week in total at all jobs or businesses?

<1> YES
<2> NO

<d> DON'T KNOW
<r> REFUSED

**OCQ122**	**FMT_YES_NO.**

[SKIP TO OCQ130p AFTER OCQ122]

OCQ125 How many hours did you work **last week** at **all** jobs or businesses?

<1-168> HOURS WORKED LAST WEEK

<d> DON'T KNOW
<r> REFUSED

**OCQ125**	**FMT_NUMERIC.**

OCQ127 Do you **usually** work 35 hours or more per week in total at all jobs or businesses?

<1> YES
<2> NO

<d> DON'T KNOW
<r> REFUSED

**OCQ127**	**FMT_YES_NO.**

OCCUPATION (OCQ)

OCQ130p I'm going to ask you some questions about your currently held job. If you have more than one job, please answer these questions thinking only of the job which is the primary source of your income.

INTERVIEWER: HIT ENTER TO CONTINUE

OCQ130 What kind of work do you do for pay?

(INTERVIEWER: DO NOT PROBE. ENTER JOB TITLE OR WHATEVER THEY TELL YOU.)

<1> ENTER RESPONSE FOLLOWED BY //

<d> DON'T KNOW
<r> REFUSED

**OCQ130**	**FMT_OCQ_TEXTCODE.**
**OCQ130_TEXT**	**$FMT_CHAR.**

OCQ140 What are your most important activities or duties on this job or business?

(For example: sells cars, keeps account books, operates printing press, finished concrete.)

(**INTERVIEWER: USE ACTIVE PROBES AS NECESSARY TO GET A CLEAR PICTURE OF WHAT THEY DO ON THEIR JOB.)**

<1> ENTER RESPONSE FOLLOWED BY //

<d> DON'T KNOW
<r> REFUSED

**OCQ140**	**FMT_OCQ_TEXTCODE.**
**OCQ140_TEXT**	**$FMT_CHAR.**

OCCUPATION (OCQ)

OCQ150 What kind of business or industry is this?

(For example: TV and radio management, retail shoe store, state labor department, farm.)

(INTERVIEWER: USE ACTIVE PROBES AS NECESSARY TO GET A CLEAR PICTURE OF THE INDUSTRY IN WHICH THEY WORK. INCLUDE THE NAME OF THE BUSINESS, JOB OR INDUSTRY)

<1> ENTER RESPONSE FOLLOWED BY //

<d> DON'T KNOW
<r> REFUSED

**OCQ150**	**FMT_OCQ_TEXTCODE.**
**OCQ150_TEXT**	**$FMT_CHAR.**

OCQ160 Is this mainly manufacturing, wholesale trade, retail trade or something else?

<1> MANUFACTURING (MAKING A PRODUCT)
<2> WHOLESALE (SELLING TO BUSINESSES)
<3> RETAIL (SELLING TO CONSUMERS)
<4> SOMETHING ELSE (FOR EXAMPLE: EDUCATION, TRANSPORTATION, GOVERNMENT, HEALTHCARE, AGRICULTURE, MINING, INSURANCE, BANKING, ENTERTAINMENT, REAL ESTATE, SERVICES, ETC.)

<d> DON'T KNOW
<r> REFUSED

**OCQ160**	**FMT_OCQ160_225_.**

OCQ170 Looking at this card, which of these **best** describes this job or work situation?

(INTERVIEWER: HAND CARD)

<1> An employee of a **private** company, business, or individual for wages, salary or commission
<2> A **federal** government employee
<3> A **state** government employee
<4> A **local** government employee
<5> Self-employed in **own** business, professional practice or farm
<6> Working **without pay** in family business or farm

<d> DON'T KNOW
<r> REFUSED

**OCQ170**	**FMT_OCQ170_226_.**

OCCUPATION (OCQ)

OCQ175 On average, how many hours per week do you work at this job?

<1-168> HOURS PER WEEK

<d> DON'T KNOW
<r> REFUSED

OCQ175 FMT_NUMERIC.

OCQ180 At any time over the past year, have you worked in agriculture? For example farming, livestock production, commercial fishing, or forestry.

<1> YES
<2> NO

<d> DON'T KNOW
<r> REFUSED

OCQ180 FMT_YES_NO.

OCQ185 At any time over the past year, have you worked or volunteered in a setting that provides healthcare to patients?

(INTERVIEWER: HAND CARD)

For example: medical clinic, doctor's office, dentist's office, hospital, nursing home or some other health-care facility. This includes emergency responders and public safety personnel, part-time and unpaid work in a health care facility as well as professional nursing care provided in the home. This also includes non-health care professionals, such as administrative staff, who work in a health-care facility.

<1> YES
<2> NO **[Skip or go to OCQ14010pre]**

<d> DON'T KNOW
<r> REFUSED **[Skip or go to OCQ14010pre]**

OCQ185 FMT_YES_NO.

OCQ186 Did you provide direct patient care as part of your routine? By direct patient care, we mean physical or hands on contact with patients?

<1> YES
<2> NO

<d> DON'T KNOW

OCCUPATION (OCQ)

<r> REFUSED

OCQ186 FMT_YES_NO.

OCQ14010pre Many people shop, exercise, and run errands near where they work. To better understand the resources that might be available to you, we would now like to collect information about your job.

INTERVIEWER: HIT ENTER TO CONTINUE

OCQ14010 What is the address of your current place of employment?

<1> ENTER ADDRESS (goto STREET)
<99> WORKS FROM HOME **(SKIP TO PAQ200pre)**

OCQ14010_1 FMT_OCQ14010_1_.

<d> DON'T KNOW **(GO TO OCQ14020)**
<r> REFUSED **(GO TO OCQ14020)**

STREET ________________________

OCQ14010_A $FMT_CHAR.

CITY____________________________

OCQ14010_B $FMT_CHAR.

ZIPCODE ________

OCQ14010_C $FMT_CHAR.

OCQ14020 How many miles is your current place of employment from your home?

<0-300> MILES

<d> DON'T KNOW
<r> REFUSED

OCQ14020 FMT_NUMERIC.

Depression diagnostic and Severity Measure (PHQ)

PHQ001 Please indicate how much you have been bothered by these problems.

This section will take about 5 minutes.

Enter **1** to continue.

<1> CONTINUE

PHQ010 Over the **past 2** weeks, how often have you been bothered by any of the following problems:

Little interest or pleasure in doing things?

Would you say not at all, several days, more than half the days, or nearly every day?

Enter **1** for **not at all**, **2** for **several days**, **3** for **more than half the days**, or **4** for **nearly every day**.

<1> NOT AT ALL **PHQ010 FMT_PHQ_OFTEN.**

<2> SEVERAL DAYS
<3> MORE THAN HALF THE DAYS
<4> NEARLY EVERY DAY

<d> DON'T KNOW **<r> REFUSED**
<q> REPLAY QUESTION <h> REPLAY RESPONSES
<y> TURN QUESTION AUDIO OFF (SOUND IS NOW **ON**)
<s> TURN RESPONSE AUDIO OFF (SOUND IS NOW **ON**)

PHQ020 Over the **past 2** weeks, how often have you been bothered by any of the following problems:

Feeling down, depressed, or hopeless?

Would you say not at all, several days, more than half the days, or nearly every day?

Enter **1** for **not at all**, **2** for **several days**, **3** for **more than half the days**, or **4** for **nearly every day**.

<1> NOT AT ALL **PHQ020 FMT_PHQ_OFTEN.**
<2> SEVERAL DAYS
<3> MORE THAN HALF THE DAYS
<4> NEARLY EVERY DAY

<d> DON'T KNOW **<r> REFUSED**
<q> REPLAY QUESTION <h> REPLAY RESPONSES

Depression diagnostic and Severity Measure (PHQ)

<y> TURN QUESTION AUDIO OFF (SOUND IS NOW **ON**)
<s> TURN RESPONSE AUDIO OFF (SOUND IS NOW **ON**)

PTSD Checklist

Below is a list of problems and complaints that people sometimes have in response to stressful life experiences.

Please read each one below, and fill in the circle to indicate how much you have been bothered by the problem in <u>the last month</u>.

		Not at all	A little bit	Moderately	Quite a bit	Extremely	
**1.**	Repeated disturbing memories, thoughts, or images of a stressful experience from the past	○	○	○	○	○	PTSD01_R2 FMT_PTSD.
**2.**	Feeling very upset when something reminded you of a stressful experience from the past	○	○	○	○	○	PTSD04_R2 FMT_PTSD.
**3.**	Avoided activities or situations because they reminded you of a stressful experience from the past	○	○	○	○	○	PTSD07_R2 FMT_PTSD.
**4.**	Feeling distant or cut off from other people	○	○	○	○	○	PTSD10_R2 FMT_PTSD.
**5.**	Having trouble falling or staying asleep	○	○	○	○	○	PTSD13_R2 FMT_PTSD.
**6.**	Feeling irritable or having angry outbursts	○	○	○	○	○	PTSD14_R2 FMT_PTSD.
**7.**	Difficulty concentrating	○	○	○	○	○	PTSD15_R2 FMT_PTSD.

PRESCRIPTION MEDICATIONS AND SELECT OTC DRUGS (RXQ)

RXQ032pre Now I'd like to gather information about any medication you might be taking.

INTERVIEWER: HIT ENTER TO CONTINUE

RXQ032 In the **past 30 days**, have you used a **prescription medicine**? Include only those products prescribed by a health professional such as a doctor, a nurse practitioner or a dentist.

RXQ032 FMT_YES_NO.

<1> YES
<2> NO **(Skip to RXQ296)**

<d> DON'T KNOW **(Skip to RXQ296)**
<r> REFUSED **(Skip to RXQ296)**

RXQ042 I'd like to see the CONTAINERS for **all** the prescription medicines that you used or took in the **past 30 days.**

INTERVIEWER: ENTER THE NAME OF EACH DRUG FROM THE PRESCRIPTION BOTTLE UNTIL NO MORE PRESCRIPTION MEDICINES, THEN HIT X TO EXIT QUESTION

<1> ENTER NAME(S)
<d> DON'T KNOW
<r> REFUSED

RXQ042@a___________________________________

RXQ042@b___________________________________

RXQ042@c ___________________________________

RXQ042@d ___________________________________

RXQ042@e___________________________________

RXQ042@f___________________________________

RXQ042@g___________________________________

RXQ042@h____________________________________

RXQ042@i ___________________________________

RXQ042@qq ARE THERE MORE PRESCRIPTION MEDICINES?

<1> YES (go to RXQ042@j) <2> NO

PRESCRIPTION MEDICATIONS AND SELECT OTC DRUGS (RXQ)

INTERVIEWER: ENTER THE NAME OF EACH DRUG FROM THE PRESCRIPTION BOTTLE UNTIL NO MORE PRESCRIPTION MEDICINES, THEN HIT X TO EXIT QUESTION

RXQ042p2@j___________________________________

RXQ042p2@k___________________________________

RXQ042p2@l ___________________________________

RXQ042p2@m ___________________________________

RXQ042p2@n ___________________________________

RXQ042p2@o___________________________________

RXQ042p2@p___________________________________

RXQ042p2@q ___________________________________

RXQ042p2@r___________________________________

RXQ042p2@s ___________________________________

RXQ042p2@t ___________________________________

**RXQ042_A**	**$FMT_CHAR.**
**RXQ042_B**	**$FMT_CHAR.**
**RXQ042_C**	**$FMT_CHAR.**
**RXQ042_D**	**$FMT_CHAR.**
**RXQ042_E**	**$FMT_CHAR.**
**RXQ042_F**	**$FMT_CHAR.**
**RXQ042_G**	**$FMT_CHAR.**
**RXQ042_H**	**$FMT_CHAR.**
**RXQ042_I**	**$FMT_CHAR.**
**RXQ042_J**	**$FMT_CHAR.**
**RXQ042_K**	**$FMT_CHAR.**
**RXQ042_L**	**$FMT_CHAR.**
**RXQ042_M**	**$FMT_CHAR.**
**RXQ042_N**	**$FMT_CHAR.**
**RXQ042_O**	**$FMT_CHAR.**
**RXQ042_P**	**$FMT_CHAR.**
**RXQ042_Q**	**$FMT_CHAR.**
**RXQ042_R**	**$FMT_CHAR.**
**RXQ042_S**	**$FMT_CHAR.**
**RXQ042_T**	**$FMT_CHAR.**

RXQ231 Are there any **prescription medications** that you have used in the **past**

PRESCRIPTION MEDICATIONS AND SELECT OTC DRUGS (RXQ)

30 days for which you no longer have a prescription bottle or container? Again, these are products prescribed by a health professional such as a doctor, a nurse practitioner or a dentist.

RXQ231 FMT_YES_NO.

<1> YES
<2> NO **(Skip to RXQ294)**

<d> DON'T KNOW **(Skip to RXQ294)**
<r> REFUSED **(Skip to RXQ294)**

RXQ235 What is the name of each such drug?

INTERVIEWER: ENTER THE NAME OF EACH DRUG UNTIL NO MORE DRUGS, THEN HIT X TO EXIT QUESTION

<1> ENTER NAME(S)
<d> DON'T KNOW
<r> REFUSED

RXQ235@a ____________________________________

RXQ235@b ____________________________________

RXQ235@c ____________________________________

RXQ235@d ____________________________________

RXQ235@e ____________________________________

RXQ235@f ____________________________________

RXQ235@g ____________________________________

RXQ235@h ____________________________________

RXQ235@i ____________________________________

RXQ235@qq ARE THERE MORE PRESCRIPTION MEDICINES?

<1> YES (go to RXQ235@j) <2> NO

INTERVIEWER: ENTER THE NAME OF EACH DRUG UNTIL NO MORE DRUGS, THEN HIT X TO EXIT QUESTION

RXQ235p2@j ____________________________________

PRESCRIPTION MEDICATIONS AND SELECT OTC DRUGS (RXQ)

RXQ235p2@k ___________________________________

RXQ235p2@l ___________________________________

RXQ235p2@m ___________________________________

RXQ235p2@n ___________________________________

RXQ235p2@o ___________________________________

RXQ235p2@p ___________________________________

RXQ235p2@q ___________________________________

RXQ235p2@r ___________________________________

RXQ235p2@s ___________________________________

RXQ235p2@t ___________________________________

**RXQ235_A**	**$FMT_CHAR.**
**RXQ235_B**	**$FMT_CHAR.**
**RXQ235_C**	**$FMT_CHAR.**
**RXQ235_D**	**$FMT_CHAR.**
**RXQ235_E**	**$FMT_CHAR.**
**RXQ235_F**	**$FMT_CHAR.**
**RXQ235_G**	**$FMT_CHAR.**
**RXQ235_H**	**$FMT_CHAR.**
**RXQ235_I**	**$FMT_CHAR.**
**RXQ235_J**	**$FMT_CHAR.**
**RXQ235_K**	**$FMT_CHAR.**
**RXQ235_L**	**$FMT_CHAR.**
**RXQ235_M**	**$FMT_CHAR.**
**RXQ235_N**	**$FMT_CHAR.**
**RXQ235_O**	**$FMT_CHAR.**
**RXQ235_P**	**$FMT_CHAR.**
**RXQ235_Q**	**$FMT_CHAR.**
**RXQ235_R**	**$FMT_CHAR.**
**RXQ235_S**	**$FMT_CHAR.**
**RXQ235_T**	**$FMT_CHAR.**

RXQ294 Are there any other prescription medications that you used in the past 30 days?

**RXQ294**	**FMT_YES_NO.**

<1> YES
<2> NO

<d> DON'T KNOW
<r> REFUSED

PRESCRIPTION MEDICATIONS AND SELECT OTC DRUGS (RXQ)

[Loop back to RXQ235 as many times as needed.]

RXQ295 I have listed {TOTAL NUMBER} prescription medication(s) that you have taken in the **past 30 days**.

(**INTERVIEWER:** REVIEW TOTAL NUMBER OF PRESCRIBED MEDICATIONS AND THEIR NAMES WITH RESPONDENT)

(INTERVIEWER: USE PAGE UP/DOWN TO NAVIGATE THROUGH THE MEDICATIONS**).**

Is this correct?

RXQ295 FMT_YES_NO.

<1> YES
<2> NO (GO BACK TO ADD MEDICATION) **[goto RXQ042]**

RXQ296 In the last 30 days, have you taken any of the following types of over the counter, non-prescription drugs?

(INTERVIEWER: ENTER ALL THAT APPLY. HAND CARD)

<1> No
<2> Low dose aspirin to protect heart
<3> Drugs for pain/analgesics (including regular dose aspirin, Motrin, Tylenol, etc.)
<4> Allergy medications
<5> Drugs to help stop smoking, including nicotine gum
<6> Drugs for intestinal problems
<8> Drugs for cold and cough
<10> Drugs to help you lose or gain weight
<11> Vitamins or minerals (including calcium supplements)
<12> Other (Specify)

<d> DON'T KNOW
<r> REFUSED

**RXQ296_A**	**FMT_RXQ296_.**
**RXQ296_B**	**FMT_RXQ296_.**
**RXQ296_C**	**FMT_RXQ296_.**
**RXQ296_D**	**FMT_RXQ296_.**
**RXQ296_E**	**FMT_RXQ296_.**
**RXQ296_F**	**FMT_RXQ296_.**
**RXQ296_G**	**FMT_RXQ296_.**
**RXQ296_H**	**FMT_RXQ296_.**
**RXQ296_I**	**FMT_RXQ296_.**
**RXQ296_OTHER**	**$FMT_CHAR.**

PRESCRIPTION MEDICATIONS AND SELECT OTC DRUGS (RXQ)

RXQ300pre The following questions are about your exposure to vitamin D from supplements.

INTERVIEWER: HIT ENTER TO CONTINUE

RXQ300 In the **past 30 days**, have you used any multivitamins, vitamin D supplements with or without calcium, or cod liver oil?

<1> YES
<2> NO **(END QUESTIONNAIRE)**

<d> DON'T KNOW **(END QUESTIONNAIRE)**
<r> REFUSED **(END QUESTIONNAIRE)**

RXQ300 FMT_YES_NO.

RXQ301 I'd like to see the CONTAINERS for any multivitamins, vitamin D supplements with or without calcium, or cod liver oil that you took in the **past 30 days**.

INTERVIEWER: ENTER THE NAME OF EACH SUPPLEMENT FROM THE BOTTLE UNTIL NO MORE SUPPLEMENTS, THEN HIT X TO EXIT QUESTION

<1> ENTER NAME(S)
<d> DON'T KNOW
<r> REFUSED

RXQ301@a____________________________________

RXQ301@b____________________________________

RXQ301@c ____________________________________

RXQ301@d ____________________________________

RXQ301@e ____________________________________

RXQ301@f ____________________________________

RXQ301_A $FMT_CHAR.
RXQ301_B $FMT_CHAR.
RXQ301_C $FMT_CHAR.
RXQ301_D $FMT_CHAR.
RXQ301_E $FMT_CHAR.
RXQ301_F $FMT_CHAR.

RXQ302 Are there any multivitamin, **vitamin D supplements or bottles of cod liver oil** that you have used in the **past 30 days for which you no longer have a bottle or container**?

PRESCRIPTION MEDICATIONS AND SELECT OTC DRUGS (RXQ)

<1> YES **(GO TO RXQ 303)**
<2> NO **(SKIP TO RXQ304pre)**

<d> DON'T KNOW **(SKIP TO RXQ304pre)**
<r> REFUSED **(SKIP TO RXQ304pre)**

RXQ302 FMT_YES_NO.

RXQ303 What is the name of each such supplement?

INTERVIEWER: ENTER THE NAME OF EACH SUPPLEMENT UNTIL NO MORE SUPPLEMENTS, THEN HIT X TO EXIT QUESTION

<1> ENTER NAME(S)
<d> DON'T KNOW
<r> REFUSED

RXQ303@a ___________________________________

RXQ303@b ___________________________________

RXQ303@c ___________________________________

RXQ303@d ___________________________________

RXQ303@e ___________________________________

RXQ303@f ___________________________________

RXQ303_A $FMT_CHAR.
RXQ303_B $FMT_CHAR.
RXQ303_C $FMT_CHAR.
RXQ303_D $FMT_CHAR.
RXQ303_E $FMT_CHAR.
RXQ303_F $FMT_CHAR.

[if RXQ301@ ne <1> and RXQ303 ne <1> END SECTION]

RXQ304pre For these questions, please only think about the last 30 days. Please answer each question as best you can, and estimate if you are not sure.

INTERVIEWER: HIT ENTER TO CONTINUE

RXQ304_# In the last 30 days, on how many days per week have you taken [Fill drug name]? Would you say none or less than 1 day per week, 1 day per week, 2 days per week, 3 to 4 days per week, 5 to 6 days per week or every day?

(**INTERVIEWER: HAND CARD)**

PRESCRIPTION MEDICATIONS AND SELECT OTC DRUGS (RXQ)

<0> NONE OR LESS THAN 1 DAY PER WEEK
<1> 1 DAY PER WEEK
<2> 2 DAYS PER WEEK
<3> 3-4 DAYS PER WEEK
<5> 5-6 DAYS PER WEEK
<7> EVERY DAY

<d> DON'T KNOW
<r> REFUSED

**RXQ304_A**	**FMT_RXQ304_.**
**RXQ304_B**	**FMT_RXQ304_.**
**RXQ304_C**	**FMT_RXQ304_.**
**RXQ304_D**	**FMT_RXQ304_.**
**RXQ304_E**	**FMT_RXQ304_.**
**RXQ304_F**	**FMT_RXQ304_.**
**RXQ304_G**	**FMT_RXQ304_.**
**RXQ304_H**	**FMT_RXQ304_.**
**RXQ304_I**	**FMT_RXQ304_.**
**RXQ304_J**	**FMT_RXQ304_.**
**RXQ304_K**	**FMT_RXQ304_.**
**RXQ304_L**	**FMT_RXQ304_.**

RXQ305_# **INTERVIEWER: ENTER THE SERVING SIZE (NUMBER OF PILLS OR CHEWS OR VOLUME OF LIQUID OR AMOUNT OF POWDER) ON THE SUPPLEMENT LABEL OF [fill drug name].**

<0.00 – 30.00> NUMBER (SERVING SIZE)

<d> DON'T KNOW
<r> REFUSED

**RXQ305_A1**	**FMT_NUMERIC.**
**RXQ305_B1**	**FMT_NUMERIC.**
**RXQ305_C1**	**FMT_NUMERIC.**
**RXQ305_D1**	**FMT_NUMERIC.**
**RXQ305_E1**	**FMT_NUMERIC.**
**RXQ305_F1**	**FMT_NUMERIC.**
**RXQ305_G1**	**FMT_NUMERIC.**
**RXQ305_H1**	**FMT_NUMERIC.**
**RXQ305_I1**	**FMT_NUMERIC.**
**RXQ305_J1**	**FMT_NUMERIC.**
**RXQ305_K1**	**FMT_NUMERIC.**
**RXQ305_L1**	**FMT_NUMERIC.**

THE UNIT FOR THE SERVING OF [fill drug name]:

<1> PILLS OR CHEWS
<2> VOLUME IN MLS OR CC
<3> VOLUME IN OUNCES (OZ)
<4> TEASPOON (TSP)
<5> TABLESPOON (TBSP)

PRESCRIPTION MEDICATIONS AND SELECT OTC DRUGS (RXQ)

<6> SCOOPS (POWDER)

<d> DON’T KNOW
<r> REFUSED

**RXQ305_A2**	**FMT_RXQ305_.**
**RXQ305_B2**	**FMT_RXQ305_.**
**RXQ305_C2**	**FMT_RXQ305_.**
**RXQ305_D2**	**FMT_RXQ305_.**
**RXQ305_E2**	**FMT_RXQ305_.**
**RXQ305_F2**	**FMT_RXQ305_.**
**RXQ305_G2**	**FMT_RXQ305_.**
**RXQ305_H2**	**FMT_RXQ305_.**
**RXQ305_I2**	**FMT_RXQ305_.**
**RXQ305_J2**	**FMT_RXQ305_.**
**RXQ305_K2**	**FMT_RXQ305_.**
**RXQ305_L2**	**FMT_RXQ305_.**

RXQ306_# **INTERVIEWER: ENTER THE IU OF VITAMIN D PER SERVING ON THE SUPPLEMENT LABEL OF [Fill drug name]**

(IF NONE, ENTER 0):

<0-50,000> IU

<d> DON’T KNOW
<r> REFUSED

**RXQ306_A**	**FMT_NUMERIC.**
**RXQ306_B**	**FMT_NUMERIC.**
**RXQ306_C**	**FMT_NUMERIC.**
**RXQ306_D**	**FMT_NUMERIC.**
**RXQ306_E**	**FMT_NUMERIC.**
**RXQ306_F**	**FMT_NUMERIC.**
**RXQ306_G**	**FMT_NUMERIC.**
**RXQ306_H**	**FMT_NUMERIC.**
**RXQ306_I**	**FMT_NUMERIC.**
**RXQ306_J**	**FMT_NUMERIC.**
**RXQ306_K**	**FMT_NUMERIC.**
**RXQ306_L**	**FMT_NUMERIC.**

RXQ307 On the days you took the {fill drug name}, how many {fill unit} did you take?

<1-30.00> {fill unit}

<d> DON’T KNOW
<r> REFUSED

**RXQ307_A**	**FMT_NUMERIC.**
**RXQ307_B**	**FMT_NUMERIC.**
**RXQ307_C**	**FMT_NUMERIC.**

PRESCRIPTION MEDICATIONS AND SELECT OTC DRUGS (RXQ)

**RXQ307_D**	**FMT_NUMERIC.**
**RXQ307_E**	**FMT_NUMERIC.**
**RXQ307_F**	**FMT_NUMERIC.**
**RXQ307_G**	**FMT_NUMERIC.**
**RXQ307_H**	**FMT_NUMERIC.**
**RXQ307_I**	**FMT_NUMERIC.**
**RXQ307_J**	**FMT_NUMERIC.**
**RXQ307_K**	**FMT_NUMERIC.**
**RXQ307_L**	**FMT_NUMERIC.**

[**REPEAT RXQ304 – RXQ307 for each drug listed at RXQ301 AND RXQ303]**

Smoking and Other Tobacco Products

The next questions are about your history of using tobacco products.

1. **Have you smoked 100 or more cigarettes in your entire life?** SMQ020_R2 FMT_YES_NO.

   ○ Yes ○ No → **Go to question 11, page 21**

2. **How old were you when you started smoking cigarettes regularly?** SMQ030_R2. FMT_NUMERIC.

   Enter age when you started smoking: ☐☐

3. **Do you smoke cigarettes now?**

   ○ Yes ○ No → **Go to question 9, page 21** SMQ040_R2 FMT_YES_NO.

4. **Is your usual cigarette brand menthol or non-menthol?**

   ○ Menthol ○ Non-menthol SMQ045_R2 FMT_SMQ045_.

5. **On average, when you smoked during the past 30 days, about how many cigarettes did you smoke per day?** ***If you smoked less than 1 cigarette per day, enter 1 (1 pack = 20 cigarettes).***

   Enter number of cigarettes per day: ☐☐ SMQ050_R2 FMT_NUMERIC.

6. **For about how many years have you smoked this amount?**

   Enter number of years: ☐☐ SMQ060_R2 FMT_NUMERIC.

7. **Would you like to completely quit smoking cigarettes?**

   ○ Yes ○ No SMQ064_R2 FMT_YES_NO.

8. **During the past 12 months, has a doctor or other health professional talked to you about your smoking?**

   ○ Yes ○ No SMQ065_R2 FMT_YES_NO.

Go to question 11, page 21 →

Please answer questions 9 and 10 *only if* you answered NO to question 3. Otherwise, begin with question 11.

9. How old were you when you stopped smoking?

Enter the age you stopped smoking: [|] SMQ120_R2 FMT_NUMERIC.

10. On average, over the entire time you smoked, about how many cigarettes did you smoke per day? *If you smoked less than 1 cigarette per day, enter 1 (1 pack = 20 cigarettes).*

Enter number of cigarettes daily: [|] SMQ140_R2 FMT_NUMERIC.

<u>Everyone should answer the following questions.</u>

Now think about a typical week.

11. How many hours per week are you currently exposed to cigarette smoke in social settings outside your own home? *(This would include time spent with friends or relatives who smoke, time spent in restaurants or taverns, or other social affairs where people are smoking.)*

Enter hours per week: [|] SMQ230_R2 FMT_NUMERIC.

12. Do any people currently smoke cigarettes inside your home?

○ Yes ○ No SMQ185_R2 FMT_YES_NO.

The next questions are about your use of tobacco products other than cigarettes (such as cigars, pipes, water pipes, hookahs, very small cigars that look like cigarettes, bidis, or cigarillos).

13. Have you <u>ever</u> smoked tobacco products other than cigarettes? *(Do not include electronic cigarettes or e-cigarettes.)*

○ Yes ○ No SMQ233_R2 FMT_YES_NO.

14. Do you <u>now</u> smoke tobacco products other than cigarettes every day, some days, or not at all?

○ Every day
○ Some days
○ Not at all

SMQ240_R2 FMT_SMQFREQ.

15. **Have you ever used any smokeless tobacco products, such as chewing tobacco, snuff, snus, dip, orbs, sticks, or strips?**

◯ Yes ◯ No SMQ250_R2 FMT_YES_NO.

16. **Do you now use any smokeless tobacco products, such as chewing tobacco, snuff, snus, dip, orbs, sticks, or strips?**

◯ Every day SMQ260_R2 FMT_SMQFREQ.
◯ Some days
◯ Not at all

17. **In the past 12 months, how often has tobacco smoke entered your living space from somewhere else in or around your home (for example, from a neighbor)?**

◯ Most of the time
◯ Often SMQ270_R2 FMT_SMQ270_.
◯ Sometimes
◯ Rarely
◯ Never

18. **During the past 7 days, on how many days did you ride in a vehicle where someone other than you was smoking tobacco?** ***If none, then enter 0.***

Enter the number of days: ☐ SMQ280_R2 FMT_NUMERIC.

◯ Don't know

19. **Not counting motorcycles, in the vehicles that you or your family members who live with you own or lease, is smoking...**

◯ Always allowed in all vehicles SMQ290_R2 FMT_SMQ290_.
◯ Sometimes allowed in at least one vehicle
◯ Never allowed in any vehicle
◯ I/we don't own or lease a vehicle

20. Have you ever used electronic cigarettes or e-cigarettes?
An electronic cigarette, or e-cigarette, is a new product that looks like a regular cigarette, but is not lighted like a cigarette. It runs on a battery and has a smoke-like vapor that is produced electronically. The vapor contains nicotine, but the e-cigarette does not contain or burn any tobacco.

- ◯ Yes
- ◯ No

SMQ300_R2 FMT_YES_NO.

21. Do you now use electronic cigarettes (e-cigarettes) every day, some days, or not at all?

- ◯ Every day
- ◯ Some days
- ◯ Not at all

SMQ310_R2 FMT_SMQFREQ.

22. Do you think secondhand smoke is...

- ◯ Very harmful to one's health
- ◯ Somewhat harmful to one's health
- ◯ Not very harmful to one's health
- ◯ Not harmful to one's health

SMQ320_R2 FMT_SMQ320_.
