# Supplementary material for: Wisconsin microbiome study, a cross-sectional investigation of dietary fibre, microbiome composition and antibiotic-resistant organisms: rationale and methods
Source: BMJ Open. 2018 Mar 27;8(3):e019450. doi: 10.1136/bmjopen-2017-019450 (PMC5875638; doi:10.1136/bmjopen-2017-019450)
Supplement: Author's manuscript [file bmjopen-2017-019450.draft_revisions.pdf]

BMJ Open

BMJ Open is committed to open peer review. As part of this commitment we make the peer review history of every article we publish publicly available.

When an article is published we post the peer reviewers' comments and the authors' responses online. We also post the versions of the paper that were used during peer review. These are the versions that the peer review comments apply to.

The versions of the paper that follow are the versions that were submitted during the peer review process. They are not the versions of record or the final published versions. They should not be cited or distributed as the published version of this manuscript.

BMJ Open is an open access journal and the full, final, typeset and author-corrected version of record of the manuscript is available on our site with no access controls, subscription charges or pay-per-view fees (http://bmjopen.bmj.com).

If you have any questions on BMJ Open's open peer review process please email
info.bmjopen@bmj.com

BMJ Open

The Wisconsin Microbiome Study, a Cross-Sectional Investigation of Dietary Fiber, Microbiome Composition, and Antibiotic-Resistant Organisms: Rationale and Methods

Journal:	*BMJ Open*
Manuscript ID	bmjopen-2017-019450
Article Type:	Protocol
Date Submitted by the Author:	01-Sep-2017
Complete List of Authors:	Eggers, Shoshannah; University of Wisconsin Madison School of Medicine and Public Health, Department of Population Health Sciences<br>Malecki, Kristen; University of Wisconsin Madison School of Medicine and Public Health, Department of Population Health Sciences<br>Peppard, Paul; University of Wisconsin Madison School of Medicine and Public Health, Department of Population Health Sciences<br>Mares, Julie; University of Wisconsin Madison School of Medicine and Public Health, Department of Ophthalmology and Visual Sciences<br>Shirley, Daniel; University of Wisconsin Madison School of Medicine and Public Health, Division of Infectious Disease, Department of Medicine<br>Shukla, Sanjay; Marshfield Clinic Research Institute, Center for Human Genetics<br>Poulsen, Keith; University of Wisconsin Madison School of Veterinary Medicine, Department of Medical Sciences<br>Gangon, RE; University of Wisconsin Madison School of Medicine and Public Health, Department of Biostatistics and Medical Informatics<br>Duster, Megan; University of Wisconsin Madison School of Medicine and Public Health, Division of Infectious Disease, Department of Medicine<br>Kates, Ashley; University of Wisconsin Madison School of Medicine and Public Health, Division of Infectious Disease, Department of Medicine<br>Suen, Garret; University of Wisconsin Madison College of Agricultural and Life Sciences, Department of Bacteriology<br>Sethi, Ajay; University of Wisconsin Madison School of Medicine and Public Health, Department of Population Health Sciences<br>Safdar, Nasia ; University of Wisconsin Madison School of Medicine and Public Health, Division of Infectious Disease, Department of Medicine; William S Middleton Memorial Veterans Hospital
Keywords:	Epidemiology < INFECTIOUS DISEASES, Public health < INFECTIOUS DISEASES, MICROBIOLOGY, MOLECULAR BIOLOGY

SCHOLARONE™
Manuscripts

The Wisconsin Microbiome Study, a Cross-Sectional Investigation of Dietary Fiber, Microbiome Composition, and Antibiotic-Resistant Organisms: Rationale and Methods

Shoshannah Eggers, BS[1]
Kristen Malecki, PhD, MPH[1]
Paul Peppard, PhD[1]
Julie Mares, PhD[2]
Daniel Shirley, MD, MS[3]
Sanjay K. Shukla, PhD[4]
Keith Poulsen, DVM, PhD[5]
Ronald Gangnon, PhD[1,6]
Megan Duster, BS, MT (ASCP)[3]
Ashley Kates, PhD[3]
Garret Suen, PhD[7]
Ajay K. Sethi, PhD, MHS[1]
Nasia Safdar, MD, PhD[3,8]

Affiliations:
1. Department of Population Health Sciences, School of Medicine and Public Health, University of Wisconsin – Madison, Madison, WI, USA; 2. Department of Ophthalmology and Visual Sciences, School of Medicine and Public Health, University of Wisconsin – Madison, Madison, WI, USA; 3. Division of Infectious Disease, Department of Medicine, School of Medicine and Public Health, University of Wisconsin – Madison, Madison, WI, USA; 4. Center for Human Genetics, Marshfield Clinic Research Institute, Marshfield, WI, USA; 5. Department of Medical Sciences, School of Veterinary Medicine, University of Wisconsin – Madison, Madison, WI, USA; 6. Department of Biostatistics and Medical Informatics, School of Medicine and Public Health, University of Wisconsin – Madison, Madison, WI, USA; 7. Department of Bacteriology, College of Agricultural and Life Sciences, University of Wisconsin – Madison, Madison, WI, USA; 8. William S. Middleton Veterans Affairs Medical Center, Madison, WI, USA.

Correspondence: Nasia Safdar, MD, PhD, e-mail: ns2@medicine.wisc.edu, address: 2500 Overlook Terrace, Madison, WI 53705.
Alternate Correspondence: Ajay K. Sethi, PhD, MHS, e-mail: ajay.sethi@wisc.edu, address: Warf Office Bldg, 610 Walnut St #707, Madison, WI 53726.

Word Count: 2995

For peer review only

ABSTRACT

Introduction: Prevention of multidrug-resistant organism (MDRO) infections, such as those caused by methicillin-resistant *Staphylococcus aureus*, vancomycin-resistant enterococci, fluoroquinolone-resistant Gram-negative bacteria, and *Clostridium difficile* is crucial. Evidence suggests that dietary fiber increases gut microbial diversity, which may help prevent colonization and subsequent infection by MDROs. The aim of the Winning the War on Antibiotic Resistance (WARRIOR) project is to examine associations of dietary fiber consumption with the composition of the gut microbiota and gut colonization by MDROs. The secondary purpose of the study is to create a biorepository of multiple body site specimens for future microbiota research.

Methods and Analysis: The WARRIOR project collects biological specimens, including nasal, oral, and skin swabs, and saliva and stool samples, along with extensive data on diet and MDRO risk factors, as an ancillary study of the Survey of the Health of Wisconsin (SHOW). The SHOW is a population-based health survey collecting data on several different health determinants and outcomes, as well as objective body measurements and biological specimens. WARRIOR participants include 600 randomly selected Wisconsin residents age 18 and over. Specimens are screened for MDRO colonization and DNA is extracted for 16S rRNA microbiota sequencing. Data will be analyzed to assess the relationship between dietary fiber, the gut microbiota composition, and gut MDRO colonization.

For peer review only - http://bmjopen.bmj.com/site/about/guidelines.xhtml

Ethics and Dissemination: The WARRIOR project is approved by the University of Wisconsin Institutional Review Board. The main results of this study will be published in a peer-reviewed scientific journal.

Keywords: Infectious Disease Epidemiology, Infectious Disease Public Health, Microbiology, Molecular Biology

Strengths and Limitations:

- This study uses a large, non-clinical, population based sample with a wide variety of exposures to MDRO risk factors.
- The extensive data and biological specimens collected by the SHOW and the WARRIOR project allow for future use in many more studies examining a variety of different hypotheses.
- The primary limitation of this study is its cross-sectional nature, however plans for follow-up data collection are underway.

BACKGROUND

Trillions of microorganisms colonize the human body and play an important role in our health by affecting metabolism, nutrition, immune function, and nervous system signaling.[1] Given their association with these varying biological mechanisms, imbalance, or dysbiosis, of the gut microbiota has been linked to many adverse health effects including increased risk for infection, obesity, diabetes, inflammatory bowel disease, allergic disease, frailty in aging, and mental health conditions.[1,2] There is no consensus on what microbial composition constitutes a

For peer review only - http://bmjopen.bmj.com/site/about/guidelines.xhtml

healthy gut microbiota, although a more diverse microbiota is thought to be better, especially in the case of healthy immune response and protection against infection.[3]

Infection with multi-drug resistant organisms (MDROs) is increasingly common and effective treatment options are rapidly decreasing.[4] Vancomycin-resistant enterococci (VRE), fluoroquinolone-resistant Gram negative bacilli (FQRGNB), methicillin-resistant *Staphylococcus aureus* (MRSA), and *Clostridium difficile* (*C. diff*) are all MDROs with the capacity to cause seriously detrimental health effects.[5] VRE often causes infections associated with hospitalization, including urinary, bloodstream, catheter and surgical wound infections.[6] FQRGNB can cause pneumonia, sepsis, meningitis, and surgical site infections.[7] *S. aureus* is carried by approximately 30% of the U.S. population, while MRSA is carried by about 1%.[8] *S. aureus* carriage can be commensal, but leads to increased risk for infection by MRSA. *C. diff* causes more than 450,000 infections, leading to 15,000 mortalities annually, and has exceeded MRSA as the most frequent cause of hospital-acquired infection.[9,10] The lack of effective treatment options for these infections also endangers the efficacy and outcomes of other medical treatments, including surgery and those for cancer.[11] MDROs are often transmitted in health care settings but are increasingly being acquired through community sources.[12] In addition to causing clinical disease, MDROs can cause asymptomatic colonization which is not only a strong predictor of future infection,[13] but can also be a source of transmission via asymptomatic carriers of MDROs.[14] Preventing colonization by MDROs is therefore vital to preventing infection.

A balanced microbiota can prevent colonization and infection with MDROs and other pathogens via several pathways. One mechanism is competitive inhibition, whereby commensal microbes compete for the same resources and mucosal binding sites as pathogenic bacteria and

For peer review only - http://bmjopen.bmj.com/site/about/guidelines.xhtml

limit their growth.[15] The makeup of the microbiota also plays a large role in the development of the immune system and continues to influence immune response and maintain homeostasis throughout our lives.[16] Beneficial bacteria within the microbiota produce cytokines, short and long chain fatty acids, and other signaling molecules that increase mucus production, and strengthen epithelial barriers, as well as increasing Type 1 T helper cell (Th1) response, all of which help to fight off pathogenic bacteria.[17]

Many factors are known to influence the composition of the human gut microbiota, including age, sex and genetics, as well as modifiable factors including birth-mode, diet, exercise, environment, smoking, cohabitation, animal contact, and use of antibiotics, probiotics, and prebiotics.[18–22] Recent literature suggests dietary factors can alter the gut microbiota and may play a role in the risk of infection by gut pathogens.[23] Dietary fiber appears promising in promoting a diverse, healthy gut microbiota by selecting for fiber-degrading microbes that produce immune-enhancing compounds like butyrate.[24] Butyrate, and other short-chain fatty acids, are end-products of microbial fermentation that can enter systemic circulation and inhibit the expression of specific pro-inflammatory cytokines.[25] Moreover, disease causing disturbances to the gut microbiota may be due to Western diets abundant in fats and simple carbohydrates but lacking in fiber.[26] Although these links between fiber and immune function via the gut microbiota are promising, there is a paucity of data on the relationship of fiber with colonization resistance against MDROs, particularly in non-clinical populations.

The purpose of the Winning the War on Antibiotic Resistance (WARRIOR) study is to examine the relationships between dietary fiber, the gut microbiota, and colonization by MDROs in a state-wide, non-clinical, population-based sample of adults, and to further create a microbiome sample repository for future research. We aim to determine the association between

For peer review only - http://bmjopen.bmj.com/site/about/guidelines.xhtml

For peer review only

diets either high or low in fiber and gut microbial diversity in order to examine the different effects of specific types of dietary fiber on the gut microbiota and MDRO colonization. The primary hypothesis is that higher dietary fiber consumption will be associated with increased gut microbial diversity and lower prevalence of MDRO colonization.

METHODS/DESIGN

Overview

The WARRIOR project aims to collect data and biological samples from 600 Wisconsin residents age 18 and over. WARRIOR is an ancillary study of the ongoing Survey of the Health of Wisconsin (SHOW), for which methods have been previously published.[27] The SHOW is an annual cross-sectional, statewide, population-based health survey, modeled after the National Health and Nutrition Examination Survey (NHANES), which collects a wide range of health, behavior, and environment data as well as objective body measurements and biological specimens. The SHOW was initiated in 2008 and the WARRIOR project is a two-year ancillary study that began at the start of the 2016 survey year. Survey components that were added to the SHOW by the WARRIOR project include additional dietary assessments, questions about MDRO risk factors, and additional specimen collection including swabs of oral, skin and nasal tissues, as well as saliva and stool samples.

Recruitment and Compensation

Subjects are enrolled for the WARRIOR project during the SHOW recruitment, and complete the WARRIOR project components in addition to the SHOW survey components. The SHOW participants age 18 and over are invited to participate in the WARRIOR project.

For peer review only - http://bmjopen.bmj.com/site/about/guidelines.xhtml

Participants complete an informed consent for both the SHOW and WARRIOR components, as approved by the University of Wisconsin-Madison Institutional Review Board. Participants are compensated for each component of the survey that they complete.

Dietary Assessment

The WARRIOR project added two dietary assessments, in addition to those already included in the SHOW, that allow for the assessment of usual total fiber intake and fiber from different sources, and intake of macronutrients, phytochemicals, vitamins and minerals. Usual diet over the past year is queried using the National Cancer Institute's diet history questionnaire II (DHQ II).[28] The second added dietary component is an Automated Self-Administered 24-Hour Dietary Assessment (ASA24) [29] completed online by participants, which queries intake over a 24-hour period. When the WARRIOR project started, participants were asked to complete the ASA24 four times. Completion of the ASA24 was found to be difficult for many participants due to lack of a reliable internet connection, as well as the length and complexity of the assessment. Completion of all four ASA24s added significantly to participant survey fatigue, and completion rates were 21% for 1 recall, 23% for 2 recalls and 16% for 3 or 4 recalls after the first five months. Ultimately, our protocol was modified to request the completion of the ASA24 twice, at appointments where there are computer and personnel assistance for online completion. Participants are compensated for attempting to complete at least one ASA24.

MDRO Risk Factor Assessment

Several risk factors for MDRO colonization, outlined in the conceptual model illustrated in Figure 1, were incorporated into the SHOW's interview and questionnaire components.

For peer review only - http://bmjopen.bmj.com/site/about/guidelines.xhtml

Exposure to domestic and farm animals are assessed because they can carry MDROs and can affect non-pathogenic components of the microbiome. We ask about farm exposure, where MDROs are often present, particularly among livestock, and the use of antibiotics and proton pump inhibitors, which can have substantial and direct effects on the bacteria within the microbiome by selecting for antibiotic resistance. Questions about exposure to hospitals and history of MDRO infection, both important predictors of future MDRO infection, are also included. Because these questions are distributed throughout the existing SHOW components, they did not suffer noticeably different completion rates from SHOW components.

Biological Sampling

In addition to the blood and urine specimens collected by the SHOW, the WARRIOR project collects saliva and stool samples, and separate swabs of the nose, mouth, and skin (combined axilla/groin). Participants self-collect a stool sample at home using a collection kit provided by the SHOW interviewer. Participants collect the stool sample within the 24 hours prior to their SHOW clinic visit and refrigerate the sample until submitting it at their appointment. Approximately 1-2 mL of saliva is collected, and swabs of the axilla/groin, nares, and buccal mucosa and tonsils are taken at the clinic appointment. All WARRIOR samples are then shipped and received at the Infectious Disease Research Laboratory at the University of Wisconsin – Madison within 24 hours, where they are immediately processed for MDRO colonization testing. While stool collection and shipment proved to be easier for participants than anticipated, saliva collection was more inconsistent than expected, as ease and rate of saliva production can vary greatly among individuals.

For peer review only - http://bmjopen.bmj.com/site/about/guidelines.xhtml

Microbiological Analysis

In 2016, swabs, saliva, and stool were screened for the presence MRSA, VRE, and FQRGNB; in 2017 screening for *C. diff* was added. Specimens are processed immediately upon receipt by the lab. Swabs are vortexed in 1 mL of tryptic soy broth (TSB) (Remel, Lenexa, KS) while 100 µL of saliva and 0.1 g of stool are used to inoculate the TSB, resulting in a total of 5 assays per subject that completes all biological components of the WARRIOR project. Broths are incubated overnight aerobically at 36 °C. Aliquots of each broth are plated to mannitol salt agar (Remel, Lenexa KS) supplemented with 4 mg/L of cefoxitin (Sigma-Aldrich, St. Louis, MO) for MRSA detection, [30] enterococcosel agar (BD/Difco, Sparks, MD) supplemented with 6 mg/L of vancomycin (Sigma-Aldrich, St. Louis, MO) for VRE detection, and MacConkey's agar (BD/Difco, Sparks, MD) supplemented with 4 mg/L of ciprofloxacin (Sigma-Aldrich, St. Louis, MO) for detection of FQRGNB. Colonies matching suspected morphology on selective agar are subcultured on blood agar plates (BAP) (BD, Sparks, MD) for identification. Identification of isolates is performed using conventional biochemical methods and identification is confirmed via sequencing of the 16S rRNA gene. Resistance to cefoxitin and ciprofloxacin are determined using Kirby-Bauer disc diffusion susceptibility testing methods and breakpoints published in the Clinical Laboratory Standards Institute (CLSI) documents M07-A10 and M100-S26.[31,32] The E-test (Bio-Merieux, Marcy l'Etoile, France) is used to determine the minimum inhibitory concentration (MIC) of vancomycin. For the added *C. diff* detection, 0.1 g of stool is inoculated into 1 mL of pre-reduced *Clostridium difficile* Brucella Broth (CDBB) and incubated anaerobically at 36 °C overnight.[33] 50 µL is plated on a *Clostridium difficile* Brucella Agar (CDBA) plate and incubated for 48 hours at 36 °C. Colonies matching suspected colony morphology are subcultured to a pre-reduced BAP and subsequently identified using Gram

For peer review only

For peer review only - http://bmjopen.bmj.com/site/about/guidelines.xhtml

staining and catalase testing. Presence of toxin genes is assessed using an in-house PCR assay and bacterial identification is confirmed via sequencing of the 16S rRNA gene.[34]

Microbiota analysis is performed using DNA extracted and purified from stool samples to address the aims of the WARRIOR project, and DNA extracted from other sample matrices will be used for future unspecified research. The purified DNA is then normalized to a concentration of 5ng/μL and amplified using PCR with barcoded primers to the 16S V4 region and sequenced on an Illumina Miseq (2x250 bp reads).[35]

Stool genomic DNA extraction:

Approximately 180-220 mg of each fecal sample is added to a 2 mL bead-beating tube containing 500 μL 2X Sodium Chloride-Tris-EDTA (STE) buffer, 300 mg of 1.0-mm-diameter zirconia/silica beads and vortexed to homogenize the stool. The sample is then centrifuged for 15 min at 4 °C at 500 x *g*. A total of 800 μL of 2X STE buffer is added to the supernatant and up to 1000 μL is transferred to a new bead-beat tube containing 0.1-mm-diameter zirconia/silica beads, and one 4 mm stainless steel bead. For chemical lysis, 115 μL of an enzymatic cocktail containing 50 μL lysozyme (10 mg/mL), 10 μL mutanolysin (1 mg/mL), 5 μL lysostaphin (5 mg/mL), and 50 μL 20% sodium dodecyl sulfate is added to each tube. Additionally, 700 μL phenol:chloroform:isoamyl alcohol is added to the sample. Bead-beat tubes are then vortexed and incubated at 56 °C for 30 min. For mechanical lysis, bead-beat tubes are vortexed and then placed in a Mini-BeadBeater-24 (Cat 112011, Biospec Products, Bartlesville, OK) and beat for 3 min. Tubes are centrifuged at 16,000 x *g* for 10 min at 4 °C. The top aqueous layer is transferred to a clean 2 mL tube and washed with an additional 500 μL phenol:chloroform:isoamyl alcohol and vortexed. The sample is then centrifuged at 16,000 x *g* for 10 min at 4 °C. The

For peer review only - http://bmjopen.bmj.com/site/about/guidelines.xhtml

phenol:chloroform:isoamuyl alcohol wash is repeated between 2 and 10 times to remove impurities from the sample until the aqueous layer is clean. The top aqueous layer is then transferred to a clean 2 mL microcentrifuge tube containing 70 µL of 3M sodium acetate and 700 µL isopropanol. The samples are inverted several times and subsequently incubated at -20 °C for 30 min to 1 hr. Each sample is centrifuged at 16,000 x *g* (4 °C) for 20 min to collect the DNA pellet, which is then washed with 500 µL cold 70% ethanol. The ethanol wash is repeated, and sample DNA pellets are dried for 5 min using a Savant SpeedVac (DNA120-230, Thermo Scientific, Waltham, MA). Finally, dried DNA pellets are re-suspended in 100 µL TE buffer and stored overnight at 4 °C or at 37 °C for one hour to dissolve the DNA pellet. Samples are then purified using NucleoSpin® Gel and PCR Clean-up kit according to manufacturer's directions (Macherey-Nagel, Germany) and eluted in 40 µL TE buffer. DNA is quantified using PicoGreen in a microplate reader (BioTek Instruments) and stored long-term at -80 °C.

Swab and saliva genomic DNA extraction:

The swab head is placed into a 2mL bead-beating tube containing 750 µL 1X PBS and 500 mg of 0.1-mm-diameter zirconia/silica beads. For chemical lysis, 25 µL of an enzymatic cocktail containing 5 µL lysozyme (10 mg/mL), 15 µL mutanolysin (1 mg/mL), and 5 µL lysostaphin (5 mg/mL) is added to each bead-beat tube and vortexed. The bead-beat tubes are then incubated at 37 °C for 30 min before 60 µL of a second enzymatic cocktail containing 10 µL proteinase K (20 mg/mL) and 50 µL 10% sodium dodecyl sulfate is added to each tube. Bead-beat tubes are then vortexed and incubated at 55 °C for 45 min. For mechanical lysis, bead-beat tubes are vortexed and then placed in a Mini-BeadBeater-24 (Cat 112011, Biospec Products, Bartlesville, OK) and beat for 3 min. Tubes are centrifuged at 16,000 x *g* for 3 min at 4 °C. The top aqueous layer is

For peer review only

For peer review only - http://bmjopen.bmj.com/site/about/guidelines.xhtml

transferred to a clean 2 mL microcentrifuge tube containing 70 µL of 3M sodium acetate and 700 µL isopropanol. The samples are inverted several times and subsequently incubated at -20 °C for 30 min to 1 hr. The following ethanol wash, pellet drying and resuspension, column purification, DNA quantification and storage steps are identical to those used in the Stool genomic DNA extraction method above.

Statistical Considerations

The proposed sample size of 600 subjects will provide 80% power to detect a partial correlation (after adjustment for covariates) of 0.125 between dietary fiber intake and the primary outcome, a diversity index using a two-sided 2.5% level test.

Raw sequencing data will be processed using mothur.[35] Contigs (overlapping sequences) will be compiled, and low-quality reads will be removed. Sequences of short length and chimeras will be detected and removed using UCHIME.[36] Sequences will be assigned to operational taxonomic units (OTUs) at the species level (97% similarity) using the GreenGenes database.[37] OTU counts and the diversity and richness indices will be calculated. Several different regression methods will be used to assess the association of the usual intake of total dietary fiber and fiber from specific sources to gut microbial diversity, as well as the relationship between fiber consumption and MDRO colonization.

DISCUSSION

Emergence of antibiotic resistance and MDROs are a global public health crisis. These infections are often very serious, leading to increased medical care usage and death. Gaining a

For peer review only

better understanding of how the gut microbiota influence colonization of MDROs will help in developing new therapeutic and prevention strategies.

This is the first statewide microbiota study assessing the relationship of MDROs and diet in a random, non-clinical, general population sample. Studies of community acquired MDROs are becoming more common, however many of these sample from community-living facilities, daycares, or within livestock workers.[38–40] This study is innovative in that it samples by household within census block groups, and participants have a wider variety of exposure levels to different community acquired MDRO risk factors.

Other than low rates of ASA24 completion, participation in the added WARRIOR project components exceeded expectations. We anticipated approximately 50% of the SHOW participants would be willing to enroll in the WARRIOR project. In the first year of recruitment however, participation rates were much higher. Most people were willing to participate by submitting one or more biological samples. Having a large part of the compensation structured around the WARRIOR project components also helped with recruitment. Incorporating the MDRO risk factor questions within the usual SHOW survey likely also helped bolster completion rates.

While this study will help us better understand the relationship of dietary fiber, the gut microbiome, and MDRO colonization, and serves as a biorepository for future analysis using the other biological samples collected, there are some limitations. Current protocols are cross-sectional; however, we plan to do longitudinal follow-up of the WARRIOR sample. Dietary intake data, and many confounding variables to be considered, are collected by self-report, although there are important exceptions (e.g., physical activity and sleep are assessed by multiday accelerometry).

For peer review only - http://bmjopen.bmj.com/site/about/guidelines.xhtml

The data collected for the WARRIOR project, in addition to the extensive SHOW data, creates a rich resource that can be used for many future studies. Future directions include investigating other components of the diet that may be associated with the gut microbiota and MDRO colonization. Given the many varied biological samples taken, a variety of relationships with the oral, skin, and nasal microbiota could also be examined. The established study infrastructure provided by the SHOW also allows for the possibility of collecting additional specimens in the future, e.g., environmental samples such as water and dust, or additional analysis of individual-level data generated from the SHOW biorepository.

ACKNOWLEDGEMENTS

Ethics Approval: The SHOW and WARRIOR projects were reviewed and approved by the University of Wisconsin Institutional Review Board (2013-0251). All subjects consented to study participation.

Competing Interests: None declared.

Funding: This work was supported by the University of Wisconsin School of Medicine and Public Health through the Wisconsin Partnership Program. This funding source had no role in the collection, analysis, or interpretation of data or the writing of this manuscript.

Contributors: SE made contributions to the acquisition of data and was a major contributor in writing the manuscript. KM and PP made contributions to design and acquisition of data and critically revised the manuscript for important intellectual content. JM, DS, SKS, KP, RG, and GS made contributions to conception and design of the study and critically revised the manuscript for important intellectual content. MD and AK made contributions to the design and acquisition of data and were involved in drafting and revising the manuscript. AS and NS made

For peer review only - http://bmjopen.bmj.com/site/about/guidelines.xhtml

substantial contributions to the conception and design of the study and acquisition of the data, and critically revised the manuscript for important intellectual content. All authors read and approved the final manuscript.

REFERENCES

1 Bull MJ, Plummer NT. Part 1: The Human Gut Microbiome in Health and Disease. *Integr Med Clin J* 2014;**13**:17–22.

2 Zapata HJ, Quagliarello VJ. The Microbiota and Microbiomein Aging: Potential Implications in Health and Age-related Diseases. *J Am Geriatr Soc* 2015;**63**:776–81. doi:10.1111/jgs.13310

3 Chang JY, Antonopoulos DA, Kalra A, *et al.* Decreased Diversity of the Fecal Microbiome in Recurrent Clostridium difficile—Associated Diarrhea. *J Infect Dis* 2008;**197**:435–8. doi:10.1086/525047

4 Marston HD, Dixon DM, Knisely JM, *et al.* Antimicrobial Resistance. *JAMA* 2016;**316**:1193–204. doi:10.1001/jama.2016.11764

5 Pacio GA, Visintainer P, Maguire G, *et al.* Natural History of Colonization With Vancomycin-Resistant Enterococci, Methicillin-Resistant Staphylococcus aureus, and Resistant Gram-Negative Bacilli Among Long-Term–Care Facility Residents. *Infect Control Hosp Epidemiol* 2003;**24**:246–50. doi:10.1086/502201

6 VRE in Healthcare Settings | HAI | CDC. https://www.cdc.gov/HAI/organisms/vre/vre.html (accessed 14 Nov 2016).

7 CDC - Gram-negative Bacteria Infections in Healthcare Settings - HAI. https://www.cdc.gov/hai/organisms/gram-negative-bacteria.html (accessed 27 Oct 2016).

8 Graham I Philip L, Lin SX, Larson EL. A U.S. Population-Based Survey of Staphylococcus aureus Colonization. *Ann Intern Med* 2006;**144**:318–25. doi:10.7326/0003-4819-144-5-200603070-00006

9 Miller BA, Chen LF, Sexton DJ, *et al.* Comparison of the Burdens of Hospital-Onset, Healthcare Facility-Associated Clostridium difficile Infection and of Healthcare-Associated Infection due to Methicillin-Resistant Staphylococcus aureus in Community Hospitals. *Infect Control Hosp Epidemiol* 2011;**32**:387–90. doi:10.1086/659156

10 Lessa FC, Mu Y, Bamberg WM, *et al.* Burden of Clostridium difficile Infection in the United States. *N Engl J Med* 2015;**372**:825–34. doi:10.1056/NEJMoa1408913

For peer review only - http://bmjopen.bmj.com/site/about/guidelines.xhtml

11 WHO | Antimicrobial resistance. WHO. http://www.who.int/mediacentre/factsheets/fs194/en/ (accessed 14 Nov 2016).

12 Vysakh PR, Jeya M. A Comparative Analysis of Community Acquired and Hospital Acquired Methicillin Resistant Staphylococcus Aureus. *J Clin Diagn Res JCDR* 2013;**7**:1339–42. doi:10.7860/JCDR/2013/5302.3139

13 Safdar N, Bradley EA. The Risk of Infection after Nasal Colonization with Staphylococcus Aureus. *Am J Med* 2008;**121**:310–5. doi:10.1016/j.amjmed.2007.07.034

14 Riggs MM, Sethi AK, Zabarsky TF, *et al.* Asymptomatic carriers are a potential source for transmission of epidemic and nonepidemic Clostridium difficile strains among long-term care facility residents. *Clin Infect Dis Off Publ Infect Dis Soc Am* 2007;**45**:992–8. doi:10.1086/521854

15 Coyte KZ, Schluter J, Foster KR. The ecology of the microbiome: Networks, competition, and stability. *Science* 2015;**350**:663–6. doi:10.1126/science.aad2602

16 Hooper LV, Littman DR, Macpherson AJ. Interactions Between the Microbiota and the Immune System. *Science* 2012;**336**:1268–73. doi:10.1126/science.1223490

17 Round JL, Mazmanian SK. The gut microbiota shapes intestinal immune responses during health and disease. *Nat Rev Immunol* 2009;**9**:313–23. doi:10.1038/nri2515

18 Yatsunenko T, Rey FE, Manary MJ, *et al.* Human gut microbiome viewed across age and geography. *Nature* 2012;**486**:222–7. doi:10.1038/nature11053

19 Dominianni C, Sinha R, Goedert JJ, *et al.* Sex, Body Mass Index, and Dietary Fiber Intake Influence the Human Gut Microbiome. *PLoS ONE* 2015;**10**. doi:10.1371/journal.pone.0124599

20 Khachatryan ZA, Ktsoyan ZA, Manukyan GP, *et al.* Predominant Role of Host Genetics in Controlling the Composition of Gut Microbiota. *PLOS ONE* 2008;**3**:e3064. doi:10.1371/journal.pone.0003064

21 Dominguez-Bello MG, Costello EK, Contreras M, *et al.* Delivery mode shapes the acquisition and structure of the initial microbiota across multiple body habitats in newborns. *Proc Natl Acad Sci* 2010;**107**:11971–5. doi:10.1073/pnas.1002601107

22 Sartor RB. Therapeutic manipulation of the enteric microflora in inflammatory bowel diseases: antibiotics, probiotics, and prebiotics. *Gastroenterology* 2004;**126**:1620–33. doi:10.1053/j.gastro.2004.03.024

23 Kau AL, Ahern PP, Griffin NW, *et al.* Human nutrition, the gut microbiome and the immune system. *Nature* 2011;**474**:327–36. doi:10.1038/nature10213

24 Kuo S-M. The Interplay Between Fiber and the Intestinal Microbiome in the Inflammatory Response. *Adv Nutr* 2013;**4**:16–28. doi:10.3945/an.112.003046

For peer review only

25 Nastasi C, Candela M, Bonefeld CM, *et al.* The effect of short-chain fatty acids on human monocyte-derived dendritic cells. *Sci Rep* 2015;**5**:16148. doi:10.1038/srep16148

26 Filippo CD, Cavalieri D, Paola MD, *et al.* Impact of diet in shaping gut microbiota revealed by a comparative study in children from Europe and rural Africa. *Proc Natl Acad Sci* 2010;**107**:14691–6. doi:10.1073/pnas.1005963107

27 Nieto FJ, Peppard PE, Engelman CD, *et al.* The Survey of the Health of Wisconsin (SHOW), a novel infrastructure for population health research: rationale and methods. *BMC Public Health* 2010;**10**:785. doi:10.1186/1471-2458-10-785

28 Diet History Questionnaire (DHQ-II) and Canadian Diet History Questionnaire (C-DHQ II). https://epi.grants.cancer.gov/dhq2/about/ (accessed 17 Jun 2017).

29 Automated Self-Administered 24-Hour (ASA24®) Dietary Assessment Tool. https://epi.grants.cancer.gov/asa24/ (accessed 17 Jul 2017).

30 Safdar N, Narans L, Gordon B, *et al.* Comparison of culture screening methods for detection of nasal carriage of methicillin-resistant Staphylococcus aureus: a prospective study comparing 32 methods. *J Clin Microbiol* 2003;**41**:3163–6.

31 Clinical and Laboratory Standards Institute. Performance standards for antimicrobial susceptibility testing; 26th informational supplement. Wayne, PA: : Clinical and Laboratory Standards Institute 2016.

32 Clinical and Laboratory Standards Institute. Methods for dilution antimicrobial susceptibility tests for bacteria that grow aerobically; approved standard—10th ed. Wayne, PA: : Clinical and Laboratory Standards Institute 2006.

33 Nerandzic MM, Donskey CJ. Effective and reduced-cost modified selective medium for isolation of Clostridium difficile. *J Clin Microbiol* 2009;**47**:397–400. doi:10.1128/JCM.01591-08

34 Warrack S, Duster M, Van Hoof S, *et al.* Clostridium difficile in a children's hospital: Assessment of environmental contamination. *Am J Infect Control* 2014;**42**:802–4. doi:10.1016/j.ajic.2014.03.008

35 Kozich JJ, Westcott SL, Baxter NT, *et al.* Development of a Dual-Index Sequencing Strategy and Curation Pipeline for Analyzing Amplicon Sequence Data on the MiSeq Illumina Sequencing Platform. *Appl Environ Microbiol* 2013;**79**:5112–20. doi:10.1128/AEM.01043-13

36 Edgar RC, Haas BJ, Clemente JC, *et al.* UCHIME improves sensitivity and speed of chimera detection. *Bioinformatics* 2011;**27**:2194–200. doi:10.1093/bioinformatics/btr381

37 DeSantis TZ, Hugenholtz P, Larsen N, *et al.* Greengenes, a Chimera-Checked 16S rRNA Gene Database and Workbench Compatible with ARB. *Appl Environ Microbiol* 2006;**72**:5069–72. doi:10.1128/AEM.03006-05

For peer review only

For peer review only - http://bmjopen.bmj.com/site/about/guidelines.xhtml

38 Mathews AA, Marina T, Appalaraju B. Prevalence of community acquired MRSA in a tertiary care facility. *J Commun Dis* 2010;**42**:249–54.

39 Neyra RC, Frisancho JA, Rinsky JL, *et al.* Multidrug-resistant and methicillin-resistant Staphylococcus aureus (MRSA) in hog slaughter and processing plant workers and their community in North Carolina (USA). *Environ Health Perspect* 2014;**122**:471–7. doi:10.1289/ehp.1306741

40 Sedighi I, Moez HJ, Alikhani MY. Nasal carriage of methicillin resistant Staphylococcus aureus and their antibiotic susceptibility patterns in children attending day-care centers. *Acta Microbiol Immunol Hung* 2011;**58**:227–34. doi:10.1556/AMicr.58.2011.3.6

FIGURE TITLE AND LEGEND

Figure 1. A conceptual model illustrating the pathways between dietary fiber consumption and MDRO colonization, including mediators and confounding factors.

For peer review only

For peer review only - http://bmjopen.bmj.com/site/about/guidelines.xhtml

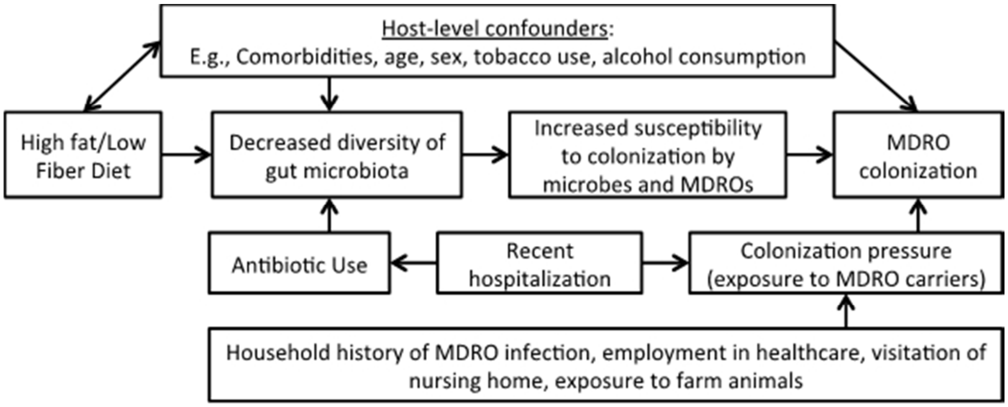

Figure 1. A conceptual model illustrating the pathways between dietary fiber consumption and MDRO colonization, including mediators and confounding factors.

161x64mm (96 x 96 DPI)

The Wisconsin Microbiome Study, a Cross-Sectional Investigation of Dietary Fiber, Microbiome Composition, and Antibiotic-Resistant Organisms: Rationale and Methods

Journal:	*BMJ Open*
Manuscript ID	bmjopen-2017-019450.R1
Article Type:	Protocol
Date Submitted by the Author:	28-Dec-2017
Complete List of Authors:	Eggers, Shoshannah; University of Wisconsin Madison School of Medicine and Public Health, Department of Population Health Sciences<br>Malecki, Kristen; University of Wisconsin Madison School of Medicine and Public Health, Department of Population Health Sciences<br>Peppard, Paul; University of Wisconsin Madison School of Medicine and Public Health, Department of Population Health Sciences<br>Mares, Julie; University of Wisconsin Madison School of Medicine and Public Health, Department of Ophthalmology and Visual Sciences<br>Shirley, Daniel; University of Wisconsin Madison School of Medicine and Public Health, Division of Infectious Disease, Department of Medicine<br>Shukla, Sanjay; Marshfield Clinic Research Institute, Center for Human Genetics<br>Poulsen, Keith; University of Wisconsin Madison School of Veterinary Medicine, Department of Medical Sciences<br>Gangon, RE; University of Wisconsin Madison School of Medicine and Public Health, Department of Biostatistics and Medical Informatics<br>Duster, Megan; University of Wisconsin Madison School of Medicine and Public Health, Division of Infectious Disease, Department of Medicine<br>Kates, Ashley; University of Wisconsin Madison School of Medicine and Public Health, Division of Infectious Disease, Department of Medicine<br>Suen, Garret; University of Wisconsin Madison College of Agricultural and Life Sciences, Department of Bacteriology<br>Sethi, Ajay; University of Wisconsin Madison School of Medicine and Public Health, Department of Population Health Sciences<br>Safdar, Nasia ; University of Wisconsin Madison School of Medicine and Public Health, Division of Infectious Disease, Department of Medicine; William S Middleton Memorial Veterans Hospital
**Primary Subject Heading**:	Infectious diseases
Secondary Subject Heading:	Epidemiology
Keywords:	Epidemiology < INFECTIOUS DISEASES, Public health < INFECTIOUS DISEASES, MICROBIOLOGY, MOLECULAR BIOLOGY

For peer review only

For peer review only - http://bmjopen.bmj.com/site/about/guidelines.xhtml

The Wisconsin Microbiome Study, a Cross-Sectional Investigation of Dietary Fiber, Microbiome Composition, and Antibiotic-Resistant Organisms: Rationale and Methods

Shoshannah Eggers, BS[1]
Kristen Malecki, PhD, MPH[1]
Paul Peppard, PhD[1]
Julie Mares, PhD[2]
Daniel Shirley, MD, MS[3]
Sanjay K. Shukla, PhD[4]
Keith Poulsen, DVM, PhD[5]
Ronald Gangnon, PhD[1,6]
Megan Duster, BS, MT (ASCP)[3]
Ashley Kates, PhD[3]
Garret Suen, PhD[7]
Ajay K. Sethi, PhD, MHS[1]
Nasia Safdar, MD, PhD[3,8]

Affiliations:
1. Department of Population Health Sciences, School of Medicine and Public Health, University of Wisconsin – Madison, Madison, WI, USA; 2. Department of Ophthalmology and Visual Sciences, School of Medicine and Public Health, University of Wisconsin – Madison, Madison, WI, USA; 3. Division of Infectious Disease, Department of Medicine, School of Medicine and Public Health, University of Wisconsin – Madison, Madison, WI, USA; 4. Center for Human Genetics, Marshfield Clinic Research Institute, Marshfield, WI, USA; 5. Department of Medical Sciences, School of Veterinary Medicine, University of Wisconsin – Madison, Madison, WI, USA; 6. Department of Biostatistics and Medical Informatics, School of Medicine and Public Health, University of Wisconsin – Madison, Madison, WI, USA; 7. Department of Bacteriology, College of Agricultural and Life Sciences, University of Wisconsin – Madison, Madison, WI, USA; 8. William S. Middleton Veterans Affairs Medical Center, Madison, WI, USA.

Correspondence: Nasia Safdar, MD, PhD, e-mail: ns2@medicine.wisc.edu, address: 2500 Overlook Terrace, Madison, WI 53705.
Alternate Correspondence: Ajay K. Sethi, PhD, MHS, e-mail: ajay.sethi@wisc.edu, address: Warf Office Bldg, 610 Walnut St #707, Madison, WI 53726.

Word Count: 3647

For peer review only - http://bmjopen.bmj.com/site/about/guidelines.xhtml

ABSTRACT

Introduction: Prevention of multidrug-resistant organism (MDRO) infections, such as those caused by methicillin-resistant *Staphylococcus aureus*, vancomycin-resistant enterococci, fluoroquinolone-resistant Gram-negative bacteria, and *Clostridium difficile* is crucial. Evidence suggests that dietary fiber increases gut microbial diversity, which may help prevent colonization and subsequent infection by MDROs. The aim of the Winning the War on Antibiotic Resistance (WARRIOR) project is to examine associations of dietary fiber consumption with the composition of the gut microbiota and gut colonization by MDROs. The secondary purpose of the study is to create a biorepository of multiple body site specimens for future microbiota research.

Methods and Analysis: The WARRIOR project collects biological specimens, including nasal, oral, and skin swabs, and saliva and stool samples, along with extensive data on diet and MDRO risk factors, as an ancillary study of the Survey of the Health of Wisconsin (SHOW). The SHOW is a population-based health survey collecting data on several different health determinants and outcomes, as well as objective body measurements and biological specimens. WARRIOR participants include 600 randomly selected Wisconsin residents age 18 and over. Specimens are screened for MDRO colonization and DNA is extracted for 16S rRNA microbiota sequencing. Data will be analyzed to assess the relationship between dietary fiber, the gut microbiota composition, and gut MDRO colonization.

For peer review only

For peer review only - http://bmjopen.bmj.com/site/about/guidelines.xhtml

Ethics and Dissemination: The WARRIOR project is approved by the University of Wisconsin Institutional Review Board. The main results of this study will be published in a peer-reviewed scientific journal.

Keywords: Infectious Disease Epidemiology, Infectious Disease Public Health, Microbiology, Molecular Biology

Strengths and Limitations:

- This study uses a large, non-clinical, population based sample with a wide variety of exposures to MDRO risk factors.
- The extensive data and biological specimens collected by the SHOW and the WARRIOR project allow for future use in many more studies examining a variety of different hypotheses.
- The primary limitation of this study is its cross-sectional nature, however plans for follow-up data collection are underway.

BACKGROUND

Trillions of microorganisms colonize the human body and play an important role in our health by affecting metabolism, nutrition, immune function, and nervous system signaling.[1] Given their association with these varying biological mechanisms, imbalance, or dysbiosis, of the gut microbiota has been linked to many adverse health effects including increased risk for infection, obesity, diabetes, inflammatory bowel disease, allergic disease, frailty in aging, and mental health conditions.[1,2] There is no consensus on what microbial composition constitutes a

For peer review only - http://bmjopen.bmj.com/site/about/guidelines.xhtml

healthy gut microbiota, although a more diverse microbiota is thought to be better, especially in the case of healthy immune response and protection against infection.[3]

Infection with multi-drug resistant organisms (MDROs) is increasingly common and effective treatment options are rapidly decreasing.[4] Vancomycin-resistant enterococci (VRE), fluoroquinolone-resistant Gram negative bacilli (FQRGNB), methicillin-resistant *Staphylococcus aureus* (MRSA), and *Clostridium difficile* (*C. diff*) are all MDROs with the capacity to cause seriously detrimental health effects.[5] VRE often causes infections associated with hospitalization, including urinary, bloodstream, catheter and surgical wound infections.[6] FQRGNB can cause pneumonia, sepsis, meningitis, and surgical site infections.[7] *S. aureus* is carried by approximately 30% of the U.S. population, while MRSA is carried by about 1%.[8] *S. aureus* carriage can be commensal, but leads to increased risk for infection by MRSA.[9] *C. diff* causes more than 450,000 infections, leading to 15,000 mortalities annually, and has exceeded MRSA as the most frequent cause of hospital-acquired infection.[10,11] The lack of effective treatment options for these infections also endangers the efficacy and outcomes of other medical treatments, including surgery and those for cancer.[12] MDROs are often transmitted in health care settings but are increasingly being acquired through community sources.[13] In addition to causing clinical disease, MDROs can cause asymptomatic colonization which is not only a strong predictor of future infection,[14] but can also be a source of transmission via asymptomatic carriers of MDROs.[15] Preventing colonization by MDROs is therefore vital to preventing infection.

A balanced microbiota can prevent colonization and infection with MDROs and other pathogens via several pathways. One mechanism is competitive inhibition, whereby commensal microbes compete for the same resources and mucosal binding sites as pathogenic bacteria and

For peer review only - http://bmjopen.bmj.com/site/about/guidelines.xhtml

limit their growth.[16] The makeup of the microbiota also plays a large role in the development of the immune system and continues to influence immune response and maintain homeostasis throughout our lives.[17] Beneficial bacteria within the microbiota produce cytokines, short and long chain fatty acids, and other signaling molecules that increase mucus production, and strengthen epithelial barriers, as well as increasing Type 1 T helper cell (Th1) response, all of which help to fight off pathogenic bacteria.[18]

Many factors are known to influence the composition of the human gut microbiota, including age, sex and genetics, as well as modifiable factors including birth-mode, diet, exercise, environment, smoking, cohabitation, animal contact, and use of antibiotics, probiotics, and prebiotics.[19–23] Recent literature suggests dietary factors can alter the gut microbiota and may play a role in the risk of infection by gut pathogens.[24] Dietary fiber appears promising in promoting a diverse, healthy gut microbiota by selecting for fiber-degrading microbes that produce immune-enhancing compounds like butyrate.[25] Butyrate, and other short-chain fatty acids, are end-products of microbial fermentation that can enter systemic circulation and inhibit the expression of specific pro-inflammatory cytokines.[26] Moreover, disease causing disturbances to the gut microbiota may be due to Western diets abundant in fats and simple carbohydrates but lacking in fiber.[27] Although these links between fiber and immune function via the gut microbiota are promising, there is a paucity of data on the relationship of fiber with colonization resistance against MDROs, particularly in non-clinical populations.

The purpose of the Winning the War on Antibiotic Resistance (WARRIOR) study is to examine the relationships between dietary fiber, the gut microbiota, and colonization by MDROs in a state-wide, non-clinical, population-based sample of adults, and to further create a microbiome sample repository for future research. We aim to determine the association between

For peer review only - http://bmjopen.bmj.com/site/about/guidelines.xhtml

For peer review only

diets either high or low in fiber and gut microbial diversity in order to examine the different effects of specific types of dietary fiber on the gut microbiota and MDRO colonization. The primary hypothesis is that higher dietary fiber consumption will be associated with increased gut microbial diversity and lower prevalence of MDRO colonization.

METHODS/DESIGN

Overview

The WARRIOR project aims to collect data and biological samples from 600 Wisconsin residents age 18 and over. WARRIOR is an ancillary study of the ongoing Survey of the Health of Wisconsin (SHOW), for which methods have been previously published.[28] A description of the WARRIOR project and the full SHOW protocol are available on the SHOW website (www.show.wisc.edu). The SHOW is an annual cross-sectional, statewide, population-based health survey, modeled after the National Health and Nutrition Examination Survey (NHANES), which collects a wide range of health, behavior, and environment data as well as objective body measurements and biological specimens. The SHOW was initiated in 2008 and the WARRIOR project is a two-year ancillary study that began at the start of the 2016 survey year. Survey components that were added to the SHOW by the WARRIOR project include additional dietary assessments, questions about MDRO risk factors, and additional specimen collection including swabs of oral, skin and nasal tissues, as well as saliva and stool samples. A study schematic outlines the various study components in Figure 1.

Recruitment and Compensation

For peer review only - http://bmjopen.bmj.com/site/about/guidelines.xhtml

Subjects are enrolled for the WARRIOR project during the SHOW recruitment, and complete the WARRIOR project components in addition to the SHOW survey components. The SHOW participants age 18 and over, all of whom meet the inclusion and exclusion criteria listed in Table 1, are invited to participate in the WARRIOR project. Participants complete an informed consent for both the SHOW and WARRIOR components, as approved by the University of Wisconsin-Madison Institutional Review Board. Participants are compensated for each component of the survey that they complete.

Table 1. List of inclusion and exclusion criteria for participation in the WARRIOR project.
Inclusion Criteria
• The selected household is the individual's usual place of residence<br>• Age 18 years or older<br>• Mentally capable of giving written informed consent<br>• Able to communicate answers to interview question
Exclusion Criteria
• Residents of nursing homes, hospitals, mental institutions, penal institutions, jails, halfway houses, or who are under the jurisdiction of the Department of Corrections<br>• Students not currently residing in the selected residence<br>• Full-time members of the armed forces or activated units of the National Guard who are currently stationed away from home and do not usually sleep in the residence<br>• Individuals who are visiting the household<br>• Individuals who have two residences and spend the greater number of nights at the other residence<br>• Individuals who have voluntarily disclosed a diagnosis of mental incapacity

Dietary Assessment

The WARRIOR project added two dietary assessments, in addition to those already included in the SHOW, that allow for the assessment of usual total fiber intake and fiber from different sources, and intake of macronutrients, phytochemicals, vitamins and minerals. Usual diet over the past year is queried using the National Cancer Institute's diet history questionnaire II (DHQ II).[29] The second added dietary component is an Automated Self-Administered 24-Hour Dietary Assessment (ASA24) [30] completed online by participants, which queries intake

For peer review only - http://bmjopen.bmj.com/site/about/guidelines.xhtml

over a 24-hour period. Dietary fiber intake will be assessed for statistical analysis by average daily grams of consumption.

When the WARRIOR project started, participants were asked to complete the ASA24 four times. Completion of the ASA24 was found to be difficult for many participants due to lack of a reliable internet connection, as well as the length and complexity of the assessment. Completion of all four ASA24s added significantly to participant survey fatigue, and completion rates were 21% for 1 recall, 23% for 2 recalls and 16% for 3 or 4 recalls after the first five months. Ultimately, our protocol was modified to request the completion of the ASA24 twice, at appointments where there are computers and personnel assistance for online completion. Participants are compensated for attempting to complete at least one ASA24.

MDRO Risk Factor Assessment

Several risk factors for MDRO colonization, outlined in the conceptual model illustrated in Figure 2, were incorporated into the SHOW's interview and questionnaire components (Supplement 1). Given the novelty of this study, standard questionnaires assessing exposure to MDRO risk factors were not readily available. Thus, questions were developed by the WARRIOR project team, a group with wide ranging expertise in microbiology, epidemiology, infectious disease, and nutrition. Questions were piloted to evaluate face validity. Exposure to domestic and farm animals are assessed because they can carry MDROs and can affect non-pathogenic components of the microbiome. We ask about farm exposure, where MDROs are often present, particularly among livestock, and the use of antibiotics and proton pump inhibitors, which can have substantial and direct effects on the bacteria within the microbiome by selecting for antibiotic resistance. Questions about exposure to hospitals and history of MDRO

For peer review only - http://bmjopen.bmj.com/site/about/guidelines.xhtml

infection, both important predictors of future MDRO infection, are also included. All SHOW and WARRIOR questionnaires and data codebooks are available at https://www.med.wisc.edu/show/data-service-center/, and MDRO risk factor assessment instruments can been found in Supplement 1. Because these questions are distributed throughout the existing SHOW components, they did not suffer noticeably different completion rates from SHOW components.

Biological Sampling

In addition to the blood and urine specimens collected by the SHOW, the WARRIOR project collects saliva and stool samples, and separate swabs of the nose, mouth, and skin (combined axilla/groin). Participants self-collect a stool sample at home using a collection kit provided by the SHOW interviewer that includes a stool collection hat, a sterile 60mL specimen cup, a sterile wood tongue depressor, gloves, a specimen label, a biohazard bag, a brown paper bag, and an instruction sheet. Participants collect the stool sample within the 24 hours prior to their SHOW clinic visit and refrigerate the sample until submitting it at their appointment. At the clinic appointment approximately 1-2 mL of saliva is collected using a sterile collection aid and a sterile tube, and swabs of the axilla/groin, nares, and buccal mucosa and tonsils are taken using a dual head BBL CultureSwab with liquid stuart transport medium (Becton, Dickinson and Company, Franklin Lakes, NJ). All WARRIOR samples are then shipped and received at the Infectious Disease Research Laboratory at the University of Wisconsin – Madison within 24 hours, where they are immediately processed for MDRO colonization testing, and then frozen at -80°C for later use in microbiome analysis. While stool collection and shipment proved to be

For peer review only - http://bmjopen.bmj.com/site/about/guidelines.xhtml

easier for participants than anticipated, saliva collection was more inconsistent than expected, as ease and rate of saliva production can vary greatly among individuals.

Microbiological Analysis

In 2016, swabs, saliva, and stool were screened for the presence MRSA, VRE, and FQRGNB; in 2017 screening for *C. diff* was added. Specimens are processed immediately upon receipt by the lab. Swabs are vortexed in 1 mL of tryptic soy broth (TSB) (Remel, Lenexa, KS) while 100 µL of saliva and 0.1 g of stool are used to inoculate the TSB, resulting in a total of 5 assays per subject that completes all biological components of the WARRIOR project. Broths are incubated overnight aerobically at 36 °C. Aliquots of each broth are plated to mannitol salt agar (Remel, Lenexa KS) supplemented with 4 mg/L of cefoxitin (Sigma-Aldrich, St. Louis, MO) for MRSA detection, [31] enterococcosel agar (BD/Difco, Sparks, MD) supplemented with 6 mg/L of vancomycin (Sigma-Aldrich, St. Louis, MO) for VRE detection, and MacConkey's agar (BD/Difco, Sparks, MD) supplemented with 4 mg/L of ciprofloxacin (Sigma-Aldrich, St. Louis, MO) for detection of FQRGNB. Colonies matching suspected morphology on selective agar are subcultured on blood agar plates (BAP) (BD, Sparks, MD) for identification. Identification of isolates is performed using conventional biochemical methods and identification is confirmed via sequencing of the 16S rRNA gene. Resistance to cefoxitin and ciprofloxacin are determined using Kirby-Bauer disc diffusion susceptibility testing methods and breakpoints published in the Clinical Laboratory Standards Institute (CLSI) documents M07-A10 and M100-S26.[32,33] The E-test (Bio-Merieux, Marcy l'Etoile, France) is used to determine the minimum inhibitory concentration (MIC) of vancomycin. For the added *C. diff* detection, 0.1 g of stool is inoculated into 1 mL of pre-reduced *Clostridium difficile* Brucella Broth (CDBB) and incubated

For peer review only - http://bmjopen.bmj.com/site/about/guidelines.xhtml

anaerobically at 36 °C overnight.[34] 50 µL is plated on a *Clostridium difficile* Brucella Agar (CDBA) plate and incubated for 48 hours at 36 °C. Colonies matching suspected colony morphology are subcultured to a pre-reduced BAP and subsequently identified using Gram staining and catalase testing. Presence of toxin genes is assessed using an in-house PCR assay and bacterial identification is confirmed via sequencing of the 16S rRNA gene.[35] All positive antibiotic resistant isolates are stocked and stored at -80°C for future unspecified research.

Microbiota analysis is performed using DNA extracted and purified from stool samples to address the aims of the WARRIOR project, and DNA extracted from other sample matrices will be used for future unspecified research. The purified DNA is then normalized to a concentration of 5ng/µL and amplified using PCR with barcoded primers to the 16S V4 region and sequenced on an Illumina Miseq (2x250 bp reads).[36] Stored DNA samples are available as a resource for additional metagenomic research and additional analyses as new technologies are developed.

Stool genomic DNA extraction:

Approximately 180-220 mg of each fecal sample is added to a 2 mL bead-beating tube containing 500 µL 2X Sodium Chloride-Tris-EDTA (STE) buffer, 300 mg of 1.0-mm-diameter zirconia/silica beads and vortexed to homogenize the stool. The sample is then centrifuged for 15 min at 4 °C at 500 x *g*. A total of 800 µL of 2X STE buffer is added to the supernatant and up to 1000 µL is transferred to a new bead-beat tube containing 0.1-mm-diameter zirconia/silica beads, and one 4 mm stainless steel bead. For chemical lysis, 115 µL of an enzymatic cocktail containing 50 µL lysozyme (10 mg/mL), 10 µL mutanolysin (1 mg/mL), 5 µL lysostaphin (5 mg/mL), and 50 µL 20% sodium dodecyl sulfate is added to each tube. Additionally, 700 µL phenol:chloroform:isoamyl alcohol is added to the sample. Bead-beat tubes are then vortexed

For peer review only - http://bmjopen.bmj.com/site/about/guidelines.xhtml

and incubated at 56 °C for 30 min. For mechanical lysis, bead-beat tubes are vortexed and then placed in a Mini-BeadBeater-24 (Cat 112011, Biospec Products, Bartlesville, OK) and beat for 3 min. Tubes are centrifuged at 16,000 x *g* for 10 min at 4 °C. The top aqueous layer is transferred to a clean 2 mL tube and washed with an additional 500 μL phenol:chloroform:isoamyl alcohol and vortexed. The sample is then centrifuged at 16,000 x *g* for 10 min at 4 °C. The phenol:chloroform:isoamuyl alcohol wash is repeated between 2 and 10 times to remove impurities from the sample until the aqueous layer is clean. The top aqueous layer is then transferred to a clean 2 mL microcentrifuge tube containing 70 μL of 3M sodium acetate and 700 μL isopropanol. The samples are inverted several times and subsequently incubated at -20 °C for 30 min to 1 hr. Each sample is centrifuged at 16,000 x *g* (4 °C) for 20 min to collect the DNA pellet, which is then washed with 500 μL cold 70% ethanol. The ethanol wash is repeated, and sample DNA pellets are dried for 5 min using a Savant SpeedVac (DNA120-230, Thermo Scientific, Waltham, MA). Finally, dried DNA pellets are re-suspended in 100 μL TE buffer and stored overnight at 4 °C or at 37 °C for one hour to dissolve the DNA pellet. Samples are then purified using NucleoSpin® Gel and PCR Clean-up kit according to manufacturer's directions (Macherey-Nagel, Germany) and eluted in 40 μL TE buffer. DNA is quantified using PicoGreen in a microplate reader (BioTek Instruments) and stored long-term at -80 °C.

Swab and saliva genomic DNA extraction:

The swab head is placed into a 2mL bead-beating tube containing 750 μL 1X PBS and 500 mg of 0.1-mm-diameter zirconia/silica beads. For chemical lysis, 25 μL of an enzymatic cocktail containing 5 μL lysozyme (10 mg/mL), 15 μL mutanolysin (1 mg/mL), and 5 μL lysostaphin (5 mg/mL) is added to each bead-beat tube and vortexed. The bead-beat tubes are then incubated at

For peer review only - http://bmjopen.bmj.com/site/about/guidelines.xhtml

37 °C for 30 min before 60 µL of a second enzymatic cocktail containing 10 µL proteinase K (20 mg/mL) and 50 µL 10% sodium dodecyl sulfate is added to each tube. Bead-beat tubes are then vortexed and incubated at 55 °C for 45 min. For mechanical lysis, bead-beat tubes are vortexed and then placed in a Mini-BeadBeater-24 (Cat 112011, Biospec Products, Bartlesville, OK) and beat for 3 min. Tubes are centrifuged at 16,000 x *g* for 3 min at 4 °C. The top aqueous layer is transferred to a clean 2 mL microcentrifuge tube containing 70 µL of 3M sodium acetate and 700 µL isopropanol. The samples are inverted several times and subsequently incubated at -20 °C for 30 min to 1 hr. The following ethanol wash, pellet drying and resuspension, column purification, DNA quantification and storage steps are identical to those used in the Stool genomic DNA extraction method above.

Statistical Considerations

The proposed sample size of 600 subjects will provide 80% power to detect a partial correlation (after adjustment for covariates) of 0.125 between dietary fiber intake and the primary outcome, a diversity index using a two-sided 2.5% level test.

Raw sequencing data will be processed using mothur.[36] Contigs (overlapping sequences) will be compiled, and low-quality reads will be removed. Sequences of short length and chimeras will be detected and removed using UCHIME.[37] Sequences will be assigned to operational taxonomic units (OTUs) at the species level (97% similarity) using the GreenGenes database.[38] OTU counts and the diversity (Shannon and Simpson) and richness (ACE and Chao) indices will be calculated.[39–41]

Several different regression methods will be used to assess the association of the usual intake of total dietary fiber and fiber from specific sources to gut microbial diversity, as well as

For peer review only - http://bmjopen.bmj.com/site/about/guidelines.xhtml

the relationship between fiber consumption and MDRO colonization. For example, to assess the association between dietary fiber consumption and gut microbial diversity, least squares linear regression will estimate mean species diversity as a function of dietary fiber. Usual grams of daily dietary fiber intake will be assessed by quantiles of consumption as fits the distribution of the data. Control variables will be added sequentially in groups; initial models will adjust only for demographic factors, subsequent models will add medications, and final models will add comorbidity and other risk factor data. Each variable in the model building process will be assessed individually, and variables that are not significant at the ≤ 0.2 level will not be included in the final model. Logistic regression models will estimate proportion of subjects colonized, dichotomized as colonized by any MDRO versus not colonized by any MDRO, as a function of dietary fiber using a similar modeling strategy.

DISCUSSION

Emergence of antibiotic resistance and MDROs are a global public health crisis. These infections are often very serious, leading to increased medical care usage and death. Gaining a better understanding of how the gut microbiota influence colonization of MDROs will help in developing new therapeutic and prevention strategies.

This is the first statewide microbiota study assessing the relationship of MDROs and diet in a random, non-clinical, general population sample. Studies of community acquired MDROs are becoming more common, however many of these sample from community-living facilities, daycares, or within livestock workers.[42–44] This study is innovative in that it samples by household within census block groups, and participants have a wider variety of exposure levels to different community acquired MDRO risk factors.

For peer review only - http://bmjopen.bmj.com/site/about/guidelines.xhtml

Other than low rates of ASA24 completion, participation in the added WARRIOR project components exceeded expectations. We anticipated approximately 50% of the SHOW participants would be willing to enroll in the WARRIOR project. In the first year of recruitment however, participation rates were much higher. Most people were willing to participate by submitting one or more biological samples. Having a large part of the compensation structured around the WARRIOR project components also helped with recruitment. Incorporating the MDRO risk factor questions within the usual SHOW survey likely also helped bolster completion rates.

While this study will help us better understand the relationship of dietary fiber, the gut microbiome, and MDRO colonization, and serves as a biorepository for future analysis using the other biological samples collected, there are some limitations. Dietary intake data, and many confounding variables to be considered, are collected by self-report, although there are important exceptions (e.g., physical activity and sleep are assessed by multiday accelerometry). The current WARRIOR project protocols are cross-sectional; however, the recently funded Population-based Microbiome Research Core (PMRC) [45] will conduct longitudinal follow-up of the WARRIOR sample. PMRC will collect an additional stool sample, environmental samples, and reassess MDRO risk factor exposures, including questions about infection history after the WARRIOR project. This data will be useful for many future studies, including analysis assessing infection risk in addition to MDRO colonization analyzed by the WARRIOR project.

The data collected for the WARRIOR project, in addition to the extensive SHOW data, creates a rich resource that can be used for many future studies. Future directions include investigating other components of the diet, and other exposures that may be associated with the gut microbiota and MDRO colonization. Given the many varied biological samples taken, a

For peer review only - http://bmjopen.bmj.com/site/about/guidelines.xhtml

variety of relationships with the oral, skin, and nasal microbiota could also be examined. Further assessment of the stool samples, including metagenomics and strain typing, is also a likely future direction. The established study infrastructure provided by the SHOW also allows for the possibility of collecting additional specimens in the future, e.g., environmental samples such as water and dust, or additional analysis of individual-level data generated from the SHOW biorepository. The ongoing infrastructure also supports additional data collection and longitudinal follow-up using these same protocols. The WARRIOR project serves as a model for population based microbiome research and findings will provide important insights into human variability and the role of the microbiome in protection or exacerbation of the global MDRO crisis.

ACKNOWLEDGEMENTS

Ethics Approval: The SHOW and WARRIOR projects were reviewed and approved by the University of Wisconsin Institutional Review Board (2013-0251). All subjects consented to study participation.

Competing Interests: None declared.

Funding: This work was supported by the University of Wisconsin School of Medicine and Public Health through the Wisconsin Partnership Program. This funding source had no role in the collection, analysis, or interpretation of data or the writing of this manuscript.

Contributors: SE made contributions to the acquisition of data and was a major contributor in writing the manuscript. KM and PP made contributions to design and acquisition of data and critically revised the manuscript for important intellectual content. JM, DS, SKS, KP, RG, and GS made contributions to conception and design of the study and critically revised the

For peer review only - http://bmjopen.bmj.com/site/about/guidelines.xhtml

manuscript for important intellectual content. MD and AK made contributions to the design and acquisition of data and were involved in drafting and revising the manuscript. AS and NS made substantial contributions to the conception and design of the study and acquisition of the data, and critically revised the manuscript for important intellectual content. All authors read and approved the final manuscript.

REFERENCES

1 Bull MJ, Plummer NT. Part 1: The Human Gut Microbiome in Health and Disease. *Integr Med Clin J* 2014;**13**:17–22.

2 Zapata HJ, Quagliarello VJ. The Microbiota and Microbiomein Aging: Potential Implications in Health and Age-related Diseases. *J Am Geriatr Soc* 2015;**63**:776–81. doi:10.1111/jgs.13310

3 Chang JY, Antonopoulos DA, Kalra A, *et al.* Decreased Diversity of the Fecal Microbiome in Recurrent Clostridium difficile—Associated Diarrhea. *J Infect Dis* 2008;**197**:435–8. doi:10.1086/525047

4 Marston HD, Dixon DM, Knisely JM, *et al.* Antimicrobial Resistance. *JAMA* 2016;**316**:1193–204. doi:10.1001/jama.2016.11764

5 Pacio GA, Visintainer P, Maguire G, *et al.* Natural History of Colonization With Vancomycin□Resistant Enterococci, Methicillin□Resistant Staphylococcus aureus, and Resistant Gram□Negative Bacilli Among Long□Term–Care Facility Residents. *Infect Control Hosp Epidemiol* 2003;**24**:246–50. doi:10.1086/502201

6 VRE in Healthcare Settings | HAI | CDC. https://www.cdc.gov/HAI/organisms/vre/vre.html (accessed 14 Nov 2016).

For peer review only - http://bmjopen.bmj.com/site/about/guidelines.xhtml

7 CDC - Gram-negative Bacteria Infections in Healthcare Settings - HAI. https://www.cdc.gov/hai/organisms/gram-negative-bacteria.html (accessed 27 Oct 2016).

8 Graham I Philip L, Lin SX, Larson EL. A U.S. Population-Based Survey of Staphylococcus aureus Colonization. *Ann Intern Med* 2006;**144**:318–25. doi:10.7326/0003-4819-144-5-200603070-00006

9 Kluytmans J a. JW, Wertheim HFL. Nasal carriage of Staphylococcus aureus and prevention of nosocomial infections. *Infection* 2005;**33**:3–8. doi:10.1007/s15010-005-4012-9

10 Miller BA, Chen LF, Sexton DJ, *et al.* Comparison of the Burdens of Hospital-Onset, Healthcare Facility-Associated Clostridium difficile Infection and of Healthcare-Associated Infection due to Methicillin-Resistant Staphylococcus aureus in Community Hospitals. *Infect Control Hosp Epidemiol* 2011;**32**:387–90. doi:10.1086/659156

11 Lessa FC, Mu Y, Bamberg WM, *et al.* Burden of Clostridium difficile Infection in the United States. *N Engl J Med* 2015;**372**:825–34. doi:10.1056/NEJMoa1408913

12 WHO | Antimicrobial resistance. WHO. http://www.who.int/mediacentre/factsheets/fs194/en/ (accessed 14 Nov 2016).

13 Vysakh PR, Jeya M. A Comparative Analysis of Community Acquired and Hospital Acquired Methicillin Resistant Staphylococcus Aureus. *J Clin Diagn Res JCDR* 2013;**7**:1339–42. doi:10.7860/JCDR/2013/5302.3139

14 Safdar N, Bradley EA. The Risk of Infection after Nasal Colonization with Staphylococcus Aureus. *Am J Med* 2008;**121**:310–5. doi:10.1016/j.amjmed.2007.07.034

15 Riggs MM, Sethi AK, Zabarsky TF, *et al.* Asymptomatic carriers are a potential source for transmission of epidemic and nonepidemic Clostridium difficile strains among long-term

For peer review only

For peer review only - http://bmjopen.bmj.com/site/about/guidelines.xhtml

care facility residents. *Clin Infect Dis Off Publ Infect Dis Soc Am* 2007;**45**:992–8. doi:10.1086/521854

16 Coyte KZ, Schluter J, Foster KR. The ecology of the microbiome: Networks, competition, and stability. *Science* 2015;**350**:663–6. doi:10.1126/science.aad2602

17 Hooper LV, Littman DR, Macpherson AJ. Interactions Between the Microbiota and the Immune System. *Science* 2012;**336**:1268–73. doi:10.1126/science.1223490

18 Round JL, Mazmanian SK. The gut microbiota shapes intestinal immune responses during health and disease. *Nat Rev Immunol* 2009;**9**:313–23. doi:10.1038/nri2515

19 Yatsunenko T, Rey FE, Manary MJ, *et al.* Human gut microbiome viewed across age and geography. *Nature* 2012;**486**:222–7. doi:10.1038/nature11053

20 Dominianni C, Sinha R, Goedert JJ, *et al.* Sex, Body Mass Index, and Dietary Fiber Intake Influence the Human Gut Microbiome. *PLoS ONE* 2015;**10**. doi:10.1371/journal.pone.0124599

21 Khachatryan ZA, Ktsoyan ZA, Manukyan GP, *et al.* Predominant Role of Host Genetics in Controlling the Composition of Gut Microbiota. *PLOS ONE* 2008;**3**:e3064. doi:10.1371/journal.pone.0003064

22 Dominguez-Bello MG, Costello EK, Contreras M, *et al.* Delivery mode shapes the acquisition and structure of the initial microbiota across multiple body habitats in newborns. *Proc Natl Acad Sci* 2010;**107**:11971–5. doi:10.1073/pnas.1002601107

23 Sartor RB. Therapeutic manipulation of the enteric microflora in inflammatory bowel diseases: antibiotics, probiotics, and prebiotics. *Gastroenterology* 2004;**126**:1620–33. doi:10.1053/j.gastro.2004.03.024

For peer review only - http://bmjopen.bmj.com/site/about/guidelines.xhtml

For peer review only

24 Kau AL, Ahern PP, Griffin NW, *et al.* Human nutrition, the gut microbiome and the immune system. *Nature* 2011;**474**:327–36. doi:10.1038/nature10213

25 Kuo S-M. The Interplay Between Fiber and the Intestinal Microbiome in the Inflammatory Response. *Adv Nutr* 2013;**4**:16–28. doi:10.3945/an.112.003046

26 Nastasi C, Candela M, Bonefeld CM, *et al.* The effect of short-chain fatty acids on human monocyte-derived dendritic cells. *Sci Rep* 2015;**5**:16148. doi:10.1038/srep16148

27 Filippo CD, Cavalieri D, Paola MD, *et al.* Impact of diet in shaping gut microbiota revealed by a comparative study in children from Europe and rural Africa. *Proc Natl Acad Sci* 2010;**107**:14691–6. doi:10.1073/pnas.1005963107

28 Nieto FJ, Peppard PE, Engelman CD, *et al.* The Survey of the Health of Wisconsin (SHOW), a novel infrastructure for population health research: rationale and methods. *BMC Public Health* 2010;**10**:785. doi:10.1186/1471-2458-10-785

29 Diet History Questionnaire (DHQ-II) and Canadian Diet History Questionnaire (C-DHQ II). https://epi.grants.cancer.gov/dhq2/about/ (accessed 17 Jun 2017).

30 Automated Self-Administered 24-Hour (ASA24®) Dietary Assessment Tool. https://epi.grants.cancer.gov/asa24/ (accessed 17 Jul 2017).

31 Safdar N, Narans L, Gordon B, *et al.* Comparison of culture screening methods for detection of nasal carriage of methicillin-resistant Staphylococcus aureus: a prospective study comparing 32 methods. *J Clin Microbiol* 2003;**41**:3163–6.

32 Clinical and Laboratory Standards Institute. Performance standards for antimicrobial susceptibility testing; 26th informational supplement. Wayne, PA: : Clinical and Laboratory Standards Institute 2016.

For peer review only - http://bmjopen.bmj.com/site/about/guidelines.xhtml

33 Clinical and Laboratory Standards Institute. Methods for dilution antimicrobial susceptibility tests for bacteria that grow aerobically; approved standard—10th ed. Wayne, PA: : Clinical and Laboratory Standards Institute 2006.

34 Nerandzic MM, Donskey CJ. Effective and reduced-cost modified selective medium for isolation of Clostridium difficile. *J Clin Microbiol* 2009;**47**:397–400. doi:10.1128/JCM.01591-08

35 Warrack S, Duster M, Van Hoof S, *et al.* Clostridium difficile in a children's hospital: Assessment of environmental contamination. *Am J Infect Control* 2014;**42**:802–4. doi:10.1016/j.ajic.2014.03.008

36 Kozich JJ, Westcott SL, Baxter NT, *et al.* Development of a Dual-Index Sequencing Strategy and Curation Pipeline for Analyzing Amplicon Sequence Data on the MiSeq Illumina Sequencing Platform. *Appl Environ Microbiol* 2013;**79**:5112–20. doi:10.1128/AEM.01043-13

37 Edgar RC, Haas BJ, Clemente JC, *et al.* UCHIME improves sensitivity and speed of chimera detection. *Bioinformatics* 2011;**27**:2194–200. doi:10.1093/bioinformatics/btr381

38 DeSantis TZ, Hugenholtz P, Larsen N, *et al.* Greengenes, a Chimera-Checked 16S rRNA Gene Database and Workbench Compatible with ARB. *Appl Environ Microbiol* 2006;**72**:5069–72. doi:10.1128/AEM.03006-05

39 Shannon CE. A mathematical theory of communication. *Bell Syst Tech J* 1948;:379–423.

40 Simpson EH. Measurement of diversity. *Nature* 1949.

41 Gotelli NJ, Colwell RK. Estimating species richness. *Biol Divers Front Meas Assess* 2011;**12**:39–54.

For peer review only

42 Mathews AA, Marina T, Appalaraju B. Prevalence of community acquired MRSA in a tertiary care facility. *J Commun Dis* 2010;**42**:249–54.

43 Neyra RC, Frisancho JA, Rinsky JL, *et al.* Multidrug-resistant and methicillin-resistant Staphylococcus aureus (MRSA) in hog slaughter and processing plant workers and their community in North Carolina (USA). *Environ Health Perspect* 2014;**122**:471–7. doi:10.1289/ehp.1306741

44 Sedighi I, Moez HJ, Alikhani MY. Nasal carriage of methicillin resistant Staphylococcus aureus and their antibiotic susceptibility patterns in children attending day-care centers. *Acta Microbiol Immunol Hung* 2011;**58**:227–34. doi:10.1556/AMicr.58.2011.3.6

45 PMRC. UW Sch. Med. Public Health. https://www.med.wisc.edu/show/pmrc/ (accessed 19 Dec 2017).

FIGURE TITLE AND LEGEND

Figure 1. A study schematic outlining components of data and specimen collection. Abbreviations: Automated Self-Administered 24-Hour Dietary Assessment (ASA24); Multi-drug resistant organism (MDRO).

Figure 2. A conceptual model illustrating the pathways between dietary fiber consumption and MDRO colonization, including mediators and confounding factors. Abbreviations: Multi-drug resistant organism (MDRO).

For peer review only

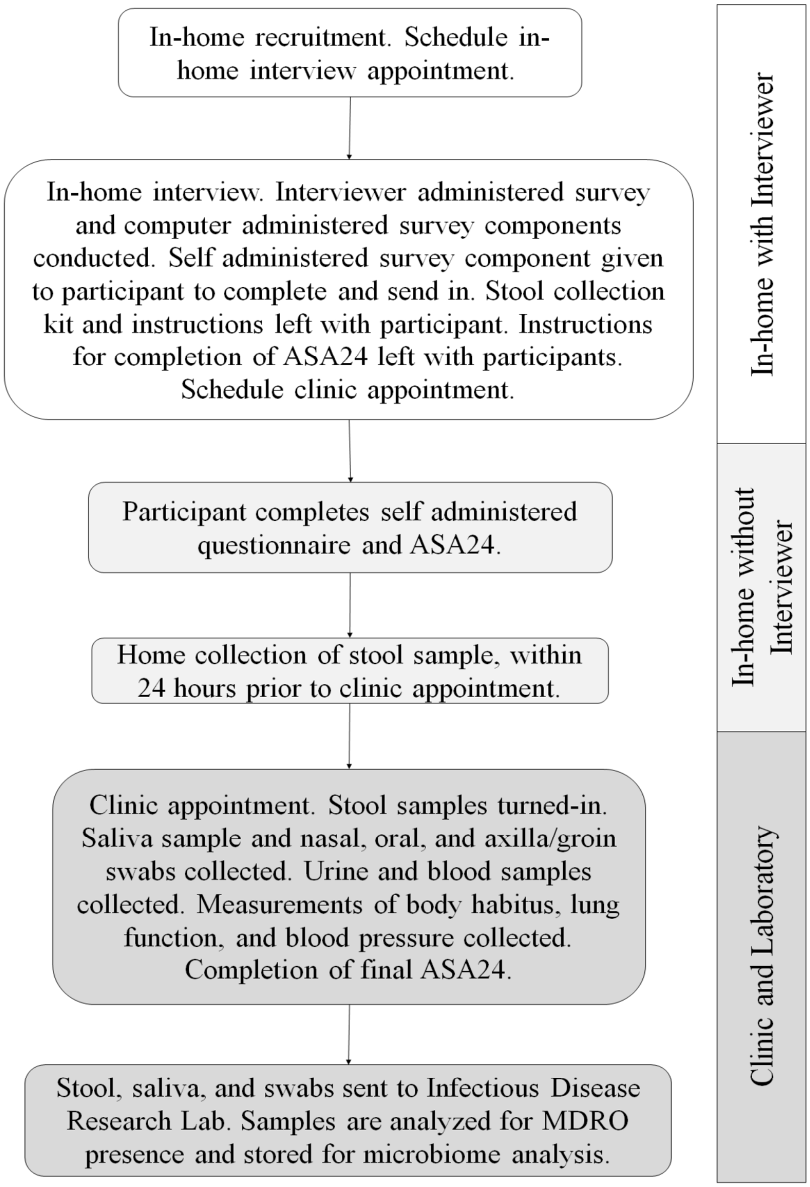

Figure 1. A study schematic outlining components of data and specimen collection. Abbreviations: Automated Self-Administered 24-Hour Dietary Assessment (ASA24); Multi-drug resistant organism (MDRO).

353x514mm (300 x 300 DPI)

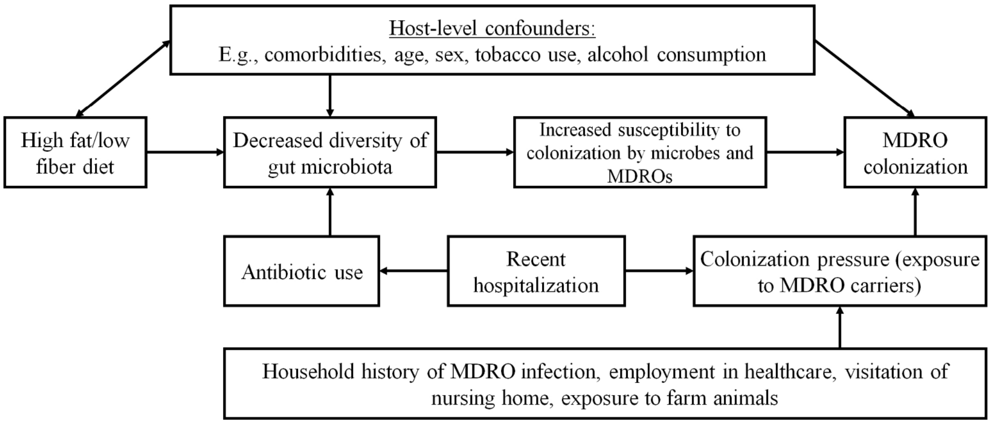

Figure 2. A conceptual model illustrating the pathways between dietary fiber consumption and MDRO colonization, including mediators and confounding factors. Abbreviations: Multi-drug resistant organism (MDRO).

187x79mm (300 x 300 DPI)

Multi-Drug Resistant Organisms-related data in the Survey of the Health of Wisconsin instruments

For peer review only

Please refer to the index below to find SHOW instruments that have data related to MDRO. All these instruments can be found in subsequent pages of this document in the order that they are listed here.

SAQ: Self-Administered Questionnaire

CAPI: Computer-Assisted Personal Interview

	Instrument	Description	Details	Mode
1.	ALQ	Alcohol	History of drinking alcoholic beverages	SAQ
2.	ANX	Animal Exposure	Time spent on a farm and with farm animals	SAQ
3.	HHQ	Health History Questionnaire	History of medical conditions, diabetes complications, questions on comorbidities; HHQ194, HHQ580, HHQ581	CAPI
19.	HMH	Household Health History (Microbiome Household)	Healthy history information regarding a participant's household that may influence his/her microbiome	SAQ
20.	HMI	Your Health History (Microbiome Individual)	Health history information that may influence a participant's microbiome	SAQ
24.	HOQ	Housing Characteristics	HOQ250_2: What kind of pets do you keep inside your home now?	SAQ
27.	IUQ	Insurance, Access, and Utilization	Questions on hospital stays; IUQ190 IUQ192, IUQ194	CAPI
40.	LAB	LAB/Sample Collection	LAB300, LAB310, LAB320, LAB330, LAB340, LAB341, LAB342, LAB350, LAB360, LAB370, LAB380, LAB390, LAB400	CAPI
52.	OCQ	Occupation	Work in healthcare setting; OCQ185, OCQ186	CAPI
58.	PHQ	Depression	Two-item depression screener; derived depression diagnosis and score: PHQ2_DX, PHQ2_SCORE	CAPI
60.	PTSD	Post-Traumatic Stress Disorder	Abbreviated PTSD Checklist – Civilian version; derived PTSD diagnosis and score: PTSD6_DX_SCORE, PTSD6_TOTAL_SCORE	CAPI
61.	RXQ	Medications	Number of medications, antibiotics	CAPI
71.	SMQ	Smoking	Smoking history	SAQ

For peer review only

Alcohol Consumption

The next set of questions are about drinking alcoholic beverages. Alcoholic beverages include liquor (such as whiskey or gin), beer, wine, wine coolers, and any other type of drink with alcohol in it.

1. **How many glasses of wine or wine coolers do you usually have per week?**
   This means 5 ounce glasses of wine or 12 ounce bottles of wine cooler (size of a regular can of soda).

   Enter number of glasses: [ ][ ] ALQ160_R2 FMT_NUMERIC

2. **How many glasses, bottles, or cans of beer do you usually have per week?**
   This means 12 ounce glasses, bottles, or cans of beer (size of a regular can of soda).

   Enter number of cans, glasses, or bottles: [ ][ ] ALQ170_R2 FMT_NUMERIC

3. **How many drinks of hard liquor do you usually have per week?**
   This means one-and-a-half ounce shots.

   Enter number of hard liquor drinks (e.g. 1.5 ounce shots): [ ][ ] ALQ180_R2 FMT_NUMERIC

4. **In the past 12 months, on how many days did you have 5 or more drinks of any alcoholic beverage?**

   If you had 5 or more alcoholic beverages about 1 day per week on average, enter 52. If you usually did this about 2 times per month, enter 24.

   If there was no day in the past 12 months where you had 5 or more drinks, enter 0.

   Enter number of days: [ ][ ][ ] ALQ130_R3 FMT_NUMERIC

5. **Was there ever a time in your life when you drank 5 or more drinks of any kind of alcoholic beverage almost every day?**

   ALQ120_R3 FMT_YES_NO

   ○ Yes
   ○ No

For peer review only - http://bmjopen.bmj.com/site/about/guidelines.xhtml

Animal Exposure

Now we would like to ask you some questions about time you have spent on a farm and with farm animals.

A farm is defined as any establishment from which $1,000 or more of agricultural products were produced or sold, or would normally have been sold, during the year.

	Yes	No	Don't know
**1. Do you live on a farm?**	○	○	○
	ANX010	FMT_YES_NO.	
**2. Do you live on a hobby farm (i.e., a small farm operated for pleasure or supplemental income rather than for primary income)?**	○	○	○
	ANX020	FMT_YES_NO.	
**3. At any time in the past year, did you work, paid or unpaid, on a farm?**	○	○	○
	ANX030	FMT_YES_NO.	

If you filled in only "No" or "Don't know" in response to questions 1 through 3, you are done with the Animal Exposure questionnaire. Please go to question 1, page 40. →

If you filled in "Yes" in response to any questions 1 through 3, please continue with question 4.

↓

4. What kinds of animals are kept on the farm on which you live or have worked *(fill in all that apply)*?

○ Beef cattle ANX040_a
○ Dairy cows ANX040_b
○ Horses ANX040_c
○ Donkeys ANX040_d
○ Llamas ANX040_e
○ Chickens ANX040_f
○ Ostriches ANX040_g
○ Pigs ANX040_h
○ Goats for dairy ANX040_i
○ Goats for meat ANX040_j
○ Sheep ANX040_k
○ Other: *Print below.* ANX040_l

ANX040_m $FMT_CHAR.

a through l are FMT_YES_NO.

5. In the past year, were antibiotics given to any of the animals raised on the farm on which you lived or worked?

○ Yes ○ No ○ Don't know ANX050 FMT_YES_NO.

For peer review only - http://bmjopen.bmj.com/site/about/guidelines.xhtml

HEALTH HISTORY PART I

HHQ100pre This next questionnaire is about your personal health history.

I will ask you if a health professional ever told you that you have or had certain common health problems in your lifetime and if so, how old you were when these occurred.

For some health problems I will ask you about the treatment you received. If there [illegible] ny[illegible] question that makes you uncomfortable you may ask me to skip the question.

INTERVIEWER: HIT ENTER TO CONTINUE

For peer review only

HHQ100 Has a doctor or other health professional ever told you that you had congestive heart failure?

HHQ100 FMT_YES_NO.

<1> YES
<2> NO

<d> [illegible]
<r> REFUSED

HHQ120 (Has a doctor or other health professional ever told you that you had [illegible])

[illegible]**angina,** also called **angina pectoris**?

HHQ120 FMT_YES_NO.

<1> YES
<2> NO

<d> [illegible]
<r> REFUSED

HHQ130 (Has a doctor or other health professional ever told you that you had [illegible])

[illegible]a **heart attack?**

HHQ130 FMT_YES_NO.

<1> YES
<2> NO **(skip to HHQ140)**

<d> [illegible] **(skip to HHQ140)**
<r> REFUSED **(skip to HHQ140)**

HHQ131 How many heart attacks have you had?

HHQ131 FMT_NUMERIC.

<1-99>

HEALTH HISTORY

<d> [illegible]
<r> REFUSED

HHQ132 How old were you when you were first told you had a heart attack?

HHQ132 FMT_NUMERIC.

<1-130> YEARS

<d> [illegible]
<r> REFUSED

HHQ140 Have you ever had heart surgery?

HHQ140 FMT_YES_NO.

<1> YES
<2> NO **(skip to HHQ150)**

<d> [illegible] **(skip to HHQ150)**
<r> REFUSED **(skip to HHQ150)**

HHQ141 Which of the following types of heart surgery have you had?

(INTERVIEWER: HAND CARD. ENTER ALL THAT APPLY.)

<1> BYPASS SURGERY
<2> ANGIOPLASTY
<3> VALVE SURGERY
<4> PACEMAKER
<5> OTHER (SPECIFY)

<d> [illegible]
<r> REFUSED

**1st RESPONSE**	**HHQ141_A**	**FMT_HHQ141_.**
**2nd RESPONSE**	**HHQ141_B**	**FMT_HHQ141_.**
**3rd RESPONSE**	**HHQ141_C**	**FMT_HHQ141_.**
**4th RESPONSE**	**HHQ141_D**	**FMT_HHQ141_.**
**5th RESPONSE**	**HHQ141_E**	**FMT_HHQ141_.**
**OTHER RESPONSE**	**HHQ141_OTHER**	**$FMT_CHAR.**

[illegible]

HHQ150 Has a doctor or other health professional ever told you that you had a transient ischemic attack (TIA)?

HHQ150 FMT_YES_NO.

<1> YES
<2> NO

<d> [illegible] <r> REFUSED

HEALTH HISTORY

HHQ160 (Has a doctor or other health professional ever told you that you had □)

□a **stroke?**

HHQ160 FMT_YES_NO.

<1> YES
<2> NO **(skip to HHQ180)**

<d> □2 1 □ 7 . 1 2 : **(skip to HHQ180)**
<r> REFUSED **(skip to HHQ180)**

HHQ162 How old were you when you were first told that you had a stroke?

HHQ162 FMT_NUMERIC.

<1-130> YEARS

<d> □2 1 □ 7 . 1 2 :
<r> REFUSED

HHQ180 (Has a doctor or other health professional ever told you that you had □)

□**high cholesterol/hyperlipidemia**?

HHQ180 FMT_YES_NO.

<1> YES
<2> NO **(skip to HHQ190)**

<d> □2 1 □ 7 . 1 2 : **(skip to HHQ190)**
<r> REFUSED **(skip to HHQ190)**

HHQ183 How is your high cholesterol/hyperlipidemia currently being treated? List all that apply.

(INTERVIEWER: HAND CARD. ENTER ALL THAT APPLY)

<1> NO TREATMENT
<2> PRESCRIBED MEDICINE
<3> WEIGHT CONTROL/LOSS
<4> EXERCISE
<5> SPECIAL DIET
<6> OTHER (SPECIFY)

<d> □2 1 □ 7 . 1 2 :
<r> REFUSED

**1st RESPONSE**	**HHQ183_A**	**FMT_HHQ183_.**
**2nd RESPONSE**	**HHQ183_B**	**FMT_HHQ183_.**
**3rd RESPONSE**	**HHQ183_C**	**FMT_HHQ183_.**

For peer review only - http://bmjopen.bmj.com/site/about/guidelines.xhtml

HEALTH HISTORY

4th RESPONSE	HHQ183_D	FMT_HHQ183_.
5th RESPONSE	HHQ183_E	FMT_HHQ183_.
OTHER RESPONSE	HHQ183_OTHER	$FMT_CHAR.

HHQ190 (Has a doctor or other health professional ever told you that you had

diabetes?

HHQ190 FMT_YES_NO.

<1> YES
<2> NO **(skip to HHQ200)**

<d> 2 1 7 . 1 2 : **(skip to HHQ200)**
<r> REFUSED **(skip to HHQ200)**

HHQ191 Which type of diabetes have you had?

(INTERVIEWER: PICK ONLY ONE)

HHQ191 FMT_HHQ191_.

<1> TYPE I
<2> TYPE II
<3> ONLY WHEN PREGNANT
<4> BORDERLINE DIABETES WHICH IS SOMETIMES CALLED PRE-DIABETES

<d> 2 1 7 . 1 2 :
<r> REFUSED

HHQ192 How old were you when you were first told you had diabetes?

HHQ192 FMT_NUMERIC.

<1-130> YEARS

<d> 2 1 7 . 1 2 :
<r> REFUSED

HHQ193 How is your diabetes currently being treated or controlled?

(INTERVIEWER: HAND CARD. ENTER ALL THAT APPLY.)

<1> NO TREATMENT
<2> INSULIN
<3> ORAL ANTI-DIABETICS (PILLS)
<4> WEIGHT CONTROL/LOSS
<5> EXERCISE
<6> SPECIAL DIET
<7> OTHER (SPECIFY)

For peer review only

For peer review only - http://bmjopen.bmj.com/site/about/guidelines.xhtml

HEALTH HISTORY

<d> 2 1 7 . 1 2 :
<r> REFUSED

**1st RESPONSE**	**HHQ193_A**	**FMT_HHQ193_.**
**2nd RESPONSE**	**HHQ193_B**	**FMT_HHQ193_.**
**3rd RESPONSE**	**HHQ193_C**	**FMT_HHQ193_.**
**4th RESPONSE**	**HHQ193_D**	**FMT_HHQ193_.**
**5th RESPONSE**	**HHQ193_E**	**FMT_HHQ193_.**
**6th RESPONSE**	**HHQ193_F**	**FMT_HHQ193_.**
**OTHER RESPONSE**	**HHQ193_OTHER**	**$FMT_CHAR.**

HHQ194 Has a doctor or other health care professional ever told you that you had any of the following complications associated with diabetes?

(INTERVIEWER: HAND CARD. ENTER ALL THAT APPLY.) ENTER X TO EXIT.

<1> NEUROPATHY (NERVE DISEASE)
<2> RETINOPATHY (EYE DISEASE)
<3> NEPHROPATHY (KIDNEY DISEASE)
<4> OTHER (SPECIFY)

<d> 2 1 7 . 1 2 :
<r> REFUSED

**1st RESPONSE**	**HHQ194_A**	**FMT_HHQ194_.**
**2nd RESPONSE**	**HHQ194_B**	**FMT_HHQ194_.**
**3rd RESPONSE**	**HHQ194_C**	**FMT_HHQ194_.**
**4th RESPONSE**	**HHQ194_D**	**FMT_HHQ194_.**
**OTHER RESPONSE**	**HHQ194_OTHER**	**$FMT_CHAR.**

HHQ200 (Has a doctor or other health professional ever told you that you had)

high blood pressure/hypertension?

HHQ200 **FMT_YES_NO.**

<1> YES
<2> NO **(skip to HHQ210)**

<d> 2 1 7 . 1 2 : **(skip to HHQ210)**
<r> REFUSED **(skip to HHQ210)**

HHQ202 How old were you when you were first told that you had **high blood pressure/hypertension**?

HHQ202 **FMT_NUMERIC.**

<1-130> YEARS

For peer review only - http://bmjopen.bmj.com/site/about/guidelines.xhtml

HEALTH HISTORY

<d> □2 1 □ 7 . 1 2 :
<r> REFUSED

HHQ203 How is your **high blood pressure/hypertension** currently treated? List all that apply.

(INTERVIEWER: HAND CARD. ENTER ALL THAT APPLY.)

<1> NO TREATMENT
<2> PRESCRIBED MEDICINE
<3> WEIGHT CONTROL/LOSS
<4> EXERCISE
<5> SPECIAL DIET
<6> OTHER (SPECIFY)

<d> □2 1 □ 7 . 1 2 :
<r> REFUSED

□ □7 □ □□ 7 2 □□□7

**1st RESPONSE**	**HHQ203_A**	**FMT_HHQ203_.**
**2nd RESPONSE**	**HHQ203_B**	**FMT_HHQ203_.**
**3rd RESPONSE**	**HHQ203_C**	**FMT_HHQ203_.**
**4th RESPONSE**	**HHQ203_D**	**FMT_HHQ203_.**
**5th RESPONSE**	**HHQ203_E**	**FMT_HHQ203_.**
**OTHER RESPONSE**	**HHQ203_OTHER**	**$FMT_CHAR.**

HHQ210 (Has a doctor or other health professional ever told you that you have □)

□**asthma**?

HHQ210 FMT_YES_NO.

<1> YES
<2> NO **(skip to HHQ230r2)**

<d> □2 1 □ 7 . 1 2 : **(skip to HHQ230r2)**
<r> REFUSED **(skip to HHQ230r2)**

HHQ212 How old were you when you were first told you have asthma?

HHQ212 FMT_NUMERIC.

<1-130> YEARS

<d> □2 1 □ 7 . 1 2 :
<r> REFUSED

HHQ214 Do you still have asthma?

HHQ214 FMT_YES_NO.

<1> YES

For peer review only

HEALTH HISTORY

<2> NO

<d> ⌧2 1 ⌧ 7 . 1 2 :
<r> REFUSED

HHQ215 During the last 12 months, have you had an episode of asthma or an asthma attack?

HHQ215 FMT_YES_NO.

<1> YES
<2> NO

<d> ⌧2 1 ⌧ 7 . 1 2 :
<r> REFUSED

HHQ216 During the past 12 months, have you visited an emergency room or urgent care because of your asthma?

HHQ216 FMT_YES_NO.

<1> YES
<2> NO

<d> ⌧2 1 ⌧ 7 . 1 2 :
<r> REFUSED

HHQ217 How is your asthma currently being treated or controlled?

(INTERVIEWER: HAND CARD. ENTER ALL THAT APPLY.)

<1> USE NOTHING/NO TREATMENT
<2> INHALED BRONCHODILATOR
<3> INHALED STERIOD
<4> ORAL MEDICATION
<5> INJECTED MEDICATIONS
<6> CONTROLLING ALLERGIES AND/OR ASTHMA TRIGGERS
<7> WEIGHT CONTROL/LOSS/EXERCISE/SPECIAL DIET
<8> OTHER (SPECIFY)

<d> ⌧2 1 ⌧ 7 . 1 2 :
<r> REFUSED

**1st RESPONSE**	**HHQ217_A**	**FMT_HHQ217_.**
**2nd RESPONSE**	**HHQ217_B**	**FMT_HHQ217_.**
**3rd RESPONSE**	**HHQ217_C**	**FMT_HHQ217_.**
**4th RESPONSE**	**HHQ217_D**	**FMT_HHQ217_.**
**5th RESPONSE**	**HHQ217_E**	**FMT_HHQ217_.**
**6th RESPONSE**	**HHQ217_F**	**FMT_HHQ217_.**
**7th RESPONSE**	**HHQ217_G**	**FMT_HHQ217_.**
**OTHER RESPONSE**	**HHQ217_OTHER**	**$FMT_CHAR.**

For peer review only

HEALTH HISTORY

HHQ218 During the past 30 days, how many days did you take a prescription asthma medication to PREVENT an asthma attack from occurring?

HHQ218 FMT_HHQ218_.

<1> NEVER
<2> 1-14 DAYS
<3> 15-24 DAYS
<4> 25-30 DAYS

<d> 2 1 7 . 1 2 :
<r> REFUSED

HHQ219 During the past 30 days, how many days did you take a prescription asthma medication DURING AN ASTHMA ATTACK to stop it?

HHQ219 FMT_HHQ219_.

<0> NEVER
<1> 1-4 TIMES
<2> 5-14 TIMES
<3> 15-29 TIMES
<4> 30-59 TIMES
<5> 60-99 TIMES
<6> MORE THAN 100 TIMES

<d> 2 1 7 . 1 2 :
<r> REFUSED

HHQ230r2 (Has a doctor or other health professional ever told you that you had)

chronic bronchitis or **emphysema?**

HHQ230_R2 FMT_YES_NO.

<1> YES
<2> NO

<d> 2 1 7 . 1 2 :
<r> REFUSED

HHQ270 (Has a doctor or other health professional ever told you that you had)

allergies or **hay fever**?

HHQ270 FMT_YES_NO.

<1> YES
<2> NO **(End of HHQ; GO TO SIQ230)**

<d> 2 1 7 . 1 2 : **(End of HHQ; GO TO SIQ230)**

For peer review only

HEALTH HISTORY

<r> REFUSED **(End of HHQ; GO TO SIC230)**

HHQ271 Which types of allergies have you had?

(INTERVIEWER: HAND CARD. ENTER ALL THAT APPLY)

<1> TREES, GRASSES, PLANTS, POLLEN
<2> MEDICINES
<3> FOODS
<4> CHEMICALS/SCENTS
<5> MOLDS
<6> ANIMALS/DANDER
<7> DUST MITES
<10> STINGING INSECTS
<11> OTHER (SPECIFY)

<d> □2 1 □ 7 . 1 2 :
<r> REFUSED

**1st RESPONSE**	**HHQ271_A**	**FMT_HHQ271_.**
**2nd RESPONSE**	**HHQ271_B**	**FMT_HHQ271_.**
**3rd RESPONSE**	**HHQ271_C**	**FMT_HHQ271_.**
**4th RESPONSE**	**HHQ271_D**	**FMT_HHQ271_.**
**5th RESPONSE**	**HHQ271_E**	**FMT_HHQ271_.**
**6th RESPONSE**	**HHQ271_F**	**FMT_HHQ271_.**
**7th RESPONSE**	**HHQ271_G**	**FMT_HHQ271_.**
**8th RESPONSE**	**HHQ271_H**	**FMT_HHQ271_.**
**9th RESPONSE**	**HHQ271_I**	**FMT_HHQ271_.**
**OTHER RESPONSE**	**HHQ271_OTHER**	**$FMT_CHAR.**

HHQ276 Do you still have allergies or hay fever?

HHQ276 FMT_YES_NO.

<1> YES
<2> NO

<d> □2 1 □ 7 . 1 2 :
<r> REFUSED

(SIQ230 and SIQ231 for MEN >=40 YEARS OLD. If <40, skip to SDQ010 below)

SIQ230 Have you ever had a prostate blood test, PSA test, and/or a rectal exam?

SIQ230 FMT_YES_NO.

<1> YES
<2> NO **(Skip to SDQ010)**

<d> □2 1 □ 7 . 1 2 **(Skip to SDQ010)**

For peer review only - http://bmjopen.bmj.com/site/about/guidelines.xhtml

HEALTH HISTORY

<r> REFUSED **(Skip to SDQ010)**

SIQ231 Within how many years?

SIQ231 FMT_NUMERIC.

INTERVIEWER: IF LESS THAN 1 YEAR, ENTER 1 FOR YEARS

<1-130> YEARS

<d> ⊠2 1 ⊠ 7 . 1 2 :
<r> REFUSED

For peer review only

SDQ010 At the **present time**, would you say your eyesight, with glasses or contact lenses, if you wear them, is excellent, good, fair, poor or very poor?

SDQ010 FMT_EGFPVP.

<1> EXCELLENT
<2> GOOD
<3> FAIR
<4> POOR
<5> VERY POOR

<d> ⊠2 1 ⊠ 7 . 1 2 :
<r> REFUSED

SDQ270 Overall, how would you describe the condition of your teeth?

Would you say excellent, very good, good, fair or poor?

SDQ270 FMT_EVGGFP.

<1> EXCELLENT
<2> VERY GOOD
<3> GOOD
<4> FAIR
<5> POOR

<d> ⊠2 1 ⊠ 7 . 1 2 :
<r> REFUSED

SDQ340 Have you ever been diagnosed by a dentist as having gum disease?

SDQ340 FMT_YES_NO.

<1> YES
<2> NO

⊠ ⊠ ⊠2 1 ⊠ 7 . 1 2 :
<r> REFUSED

For peer review only - http://bmjopen.bmj.com/site/about/guidelines.xhtml

HEALTH HISTORY

SDQ360 During the past 12 months, was there a time when you needed dental care but did not get it at that time?

SDQ360 FMT_YES_NO.

<1> YES
<2> NO **(Skip to HHQ400)**

<d> [illegible] **(Skip to HHQ400)**
<r> REFUSED **(Skip to HHQ400)**

For peer review only

SDQ361 What were the reasons that you could not get the dental care you needed?

(INTERVIEWER: ENTER ALL THAT APPLY. HAND CARD)

<1> COULD NOT AFFORD THE COST
<2> DID NOT WANT TO SPEND THE MONEY
<3> DO NOT HAVE INSURANCE
<4> INSURANCE DID NOT COVER RECOMMENDED PROCEDURES

<5> INSURANCE ONLY COVERS A PORTION OF THE COST
<6> DENTAL OFFICE IS TOO FAR AWAY
<7> DENTAL OFFICE IS NOT OPEN AT CONVENIENT TIMES

<8> ANOTHER DENTIST RECOMMENDED NOT DOING IT
<9> AFRAID, OR DO NOT LIKE DENTISTS
<10> UNABLE TO TAKE TIME OFF FROM WORK
<11> TOO BUSY
<12> I DID NOT THINK ANYTHING SERIOUS WAS WRONG--EXPECTED DENTAL PROBLEMS TO GO AWAY
<13> THE DENTIST WOULD NOT ACCEPT MY INSURANCE
<14> OTHER (SPECIFY)

<d> [illegible]
<r> REFUSED

[illegible]

**1st response**	**SDQ361_A**	**FMT_SDQ361_.**
**2nd response**	**SDQ361_B**	**FMT_SDQ361_.**
**3rd response**	**SDQ361_C**	**FMT_SDQ361_.**
**4th response**	**SDQ361_D**	**FMT_SDQ361_.**
**5th response**	**SDQ361_E**	**FMT_SDQ361_.**
**6th response**	**SDQ361_F**	**FMT_SDQ361_.**
**7th response**	**SDQ361_G**	**FMT_SDQ361_.**
**8th response**	**SDQ361_H**	**FMT_SDQ361_.**
**9th response**	**SDQ361_I**	**FMT_SDQ361_.**
**10th response**	**SDQ361_J**	**FMT_SDQ361_.**
**11th response**	**SDQ361_K**	**FMT_SDQ361_.**
**12th response**	**SDQ361_L**	**FMT_SDQ361_.**
**13th response**	**SDQ361_M**	**FMT_SDQ361_.**
**14th response**	**SDQ361_N**	**FMT_SDQ361_.**

For peer review only - http://bmjopen.bmj.com/site/about/guidelines.xhtml

HEALTH HISTORY

Other response SDQ361_OTHER $FMT_CHAR.

HHQ400 Has a doctor or other health professional ever told you that you were overweight?

HHQ400 FMT_YES_NO.

<1> YES
<2> NO **(Skip to HHQ480)**

<d> □2 1 □ 7 . 1 2 : **(Skip to HHQ480)**
<r> REFUSED **(Skip to HHQ480)**

HHQ412 How old were you when you were first told you were overweight?

HHQ412 FMT_NUMERIC.

<0-130> YEARS OLD

<d> □2 1 □ 7 . 1 2 :
<r> REFUSED

HHQ480 Has a doctor or other health professional ever told you that you had cancer?

HHQ480 FMT_YES_NO.

<1> YES
<2> NO **(Skip to HHQ500int)**

<d> □2 1 □ 7 . 1 2 : **(Skip to HHQ500int)**
<r> REFUSED **(Skip to HHQ500int)**

HHQ481 Which types of cancer on this card have you had?

(INTERVIEWER: ENTER □ / / 7 □ □ 7 □ 3 3 / □ □ 1 7 □ 5 □ □ □ : □ □ 1

<10> BLADDER
<11> BLOOD
<12> BONE
<13> BRAIN
<14> BREAST
<15> CERVIX/CERVICAL
<16> COLON
<17> ESOPHAGUS
<18> GALLBLADDER
<19> KIDNEY
<20> LARYNX/WINDPIPE
<21> LEUKEMIA
<22> LIVER
<23> LUNG
<24> LYMPHOMA/ HODGKINS DISEASE
<25> MELANOMA
<27> NERVOUS SYSTEM
<28> OVARY/OVARIAN
<29> PANCREAS/PANCREATIC
<30> PROSTATE
<31> RECTUM/RECTAL
<32> SKIN (NON MELANOMA)
<33> SKIN (UNKNOWN)
<34> SOFT TISSUE (MUSCLE/FAT)
<35> STOMACH
<36> TESTES/TESTICULAR
<37> THYROID
<38> UTERUS/UTERINE
<39> OTHER

<66> MORE THAN 3

<d> □2 1 □ 7 . 1 2 :

For peer review only - http://bmjopen.bmj.com/site/about/guidelines.xhtml

HEALTH HISTORY

<26> MOUTH/TONGUE/LIP <r> REFUSED

HHQ481_A FMT_HHQ481_.
HHQ481_B FMT_HHQ481_.
HHQ481_C FMT_HHQ481_.
HHQ481_D FMT_HHQ481_.
HHQ481_OTHER $FMT_CHAR.

HHQ500int Now we will ask you questions about certain illnesses that have occurred in your biological or blood relatives--- your grandparents, parents, aunts, uncles, brothers, sisters, and children. Please do not include half or step sisters or brothers, cousins, nieces, nephews, or grandchildren. Please include both living and deceased relatives.

INTERVIEWER: HIT ENTER TO CONTINUE

HHQ510_R2 Were any of your biological or blood relatives ever told by a doctor or other health professional that they had diabetes?

HHQ510 FMT_YES_NO.

<1> YES
<2> NO

<d> □2 1 □ 7 . 1 2 :
<r> REFUSED

HHQ520_R2 (Were any of your biological or blood relatives ever told by a doctor or other health professional that they had □

□ □ O □□ □ □ P □ □ □ □ □ □ □ □ □ □ □ □ □ □ □ P □ □ □ □ □ □

HHQ520 FMT_YES_NO.

<1> YES
<2> NO

<d> □2 1 □ 7 . 1 2 :
<r> REFUSED

HHQ530_R2 (Were any of your biological or blood relatives ever told by a doctor or other health professional that they had □

□asthma?

HHQ530 FMT_YES_NO.

<1> YES
<2> NO

For peer review only

HEALTH HISTORY

<d> [illegible]
<r> REFUSED

HHQ550_R2 (Were any of your biological or blood relatives ever told by a doctor or other health professional that they had [illegible]

[illegible]high blood pressure or hypertension?

HHQ550 FMT_YES_NO.

<1> YES
<2> NO

<d> [illegible]
<r> REFUSED

HHQ570_R3 (Were any of your biological or blood relatives ever told by a doctor or other health professional that they had [illegible]

[illegible] a heart attack or a stroke?

HHQ570_R2 FMT_YES_NO.

<1> YES
<2> NO **(Skip to HHQ580new)**

<d> [illegible] **(Skip to HHQ580new)**
<r> REFUSED **(Skip to HHQ580new)**

HHQ572_R3 What was the youngest age at which any biological or blood relative was first diagnosed with heart attack or a stroke?

HHQ572_R2 FMT_NUMERIC.

<0-130> YEARS OLD

<d> [illegible]
<r> REFUSED

HHQ580new Has a doctor or other health professional ever told you that you had any of the following?
(INTERVIEWER: HAND CARD. ENTER [illegible] FOR [illegible] NONE OR FOR NO FURTHER DIAGNOSES)

<a> ALCOHOL ABUSE
<b> [illegible]
<c> ANEMIA
<d> ANXIETY
<e> AUTISM SPECTRUM DISORDER
<t> KIDNEY STONES
<u> LEARNING DISABILITY
<ua> LIVER DISEASE
<v> LYME DISEASE
<w> MIGRAINE HEADACHE
<x> MILD COGNITIVE IMPAIRMENT

For peer review only

HEALTH HISTORY

<f>	CELIAC DISEASE	<y>	MULTIPLE SCLEROSIS
<g>	CHLAMYDIA	<z>	OSTEOARTHRITIS
<h>	[illegible]	<aa>	OSTEOPOROSIS
<i>	CHRONIC KIDNEY DISEASE	<bb>	[illegible]
<j>	DEPRESSION	<cc>	POST TRAUMATIC STRESS DISORDER (PTSD)
<k>	DRUG ABUSE	<dd>	PSORIASIS
<l>	ECZEMA/DERMATITIS	<ee>	REFLUX/GERD
<m>	EPILEPSY	<ff>	RHEUMATOID ARTHRITIS
<n>	GONERRHEA	<gg>	SHINGLES OR CHICKEN POX
<oa>	HEPATITIS A	<hh>	SICKLE CELL DISEASE
<ob>	HEPATITIS B	<ii>	STOMACH OR INTESTINAL ULCER
<oc>	HEPATITIS C	<jj>	SYPHILIS
<p>	HERPES TYPE 1/COLD SORES	<kk>	TUBERCULOSIS
<q>	HIV INFECTION/AIDS	<ll>	URINARY INCONTINENCE
<r>	HUMAN PAPILLOMA VIRUS (HPV)	<mm>	URINARY TRACT INFECTION
<s>	IRRITABLE BOWEL SYNDROME		

<xd> [illegible] <xr> REFUSED
<xx> NO FURTHER DIAGNOSES

HHQ580_A $FMT_DISEASE.
HHQ580_B $FMT_DISEASE.
HHQ580_C $FMT_DISEASE.
HHQ580_D $FMT_ DISEASE.
HHQ580_E $FMT_ DISEASE.
HHQ580_F $FMT_ DISEASE.
HHQ580_G $FMT_ DISEASE.
HHQ580_H $FMT_ DISEASE.
HHQ580_I $FMT_ DISEASE.
HHQ580_J $FMT_ DISEASE.
HHQ580_K $FMT_ DISEASE.
HHQ580_L $FMT_ DISEASE.

HHQ581 Has a doctor or other health professional ever told you that you had any of these following conditions?

(INTERVIEWER: HAND CARD. ENTER ALL THAT APPLY, [illegible] FOR [illegible] NONE OR FOR NO FURTHER DIAGNOSES)

<1> CONNECTIVE TISSUE DISEASE
<2> PERIPHERAL VASCULAR DISEASE
<3> HEMIPLEGIA
<4> SKIN OR SOFT TISSUE INFECTION

<d> [illegible] <r> REFUSED
<x> NO FURTHER DIAGNOSES

HHQ581_A FMT_HHQ581_R2_.

For peer review only - http://bmjopen.bmj.com/site/about/guidelines.xhtml

HEALTH HISTORY

HHQ581_B FMT_HHQ581_R2_.
HHQ581_C FMT_HHQ581_R2_.
HHQ581_D FMT_HHQ581_R2_.

CGQ_intro There are situations in which people provide regular **unpaid care or assistance** to a family member **including children** or a friend who has **a long-term illness or a disability**.

INTERVIEWER: HIT ENTER TO CONTINUE

CGQ010 **In the past 12 months**, did you provide any such care or assistance to a family member or friend living with you or living elsewhere?

CGQ010 FMT_YES_NO.

<1> YES
<2> NO **(Skip to RXQ032pre)**

<d> [illegible]2 1 [illegible] 7 . 1 2 : **(Go to CGQ020)**
<r> REFUSED **(Skip to RXQ032pre)**

CGQ020 Are you currently giving unpaid help to a family member or friend?

CGQ020 FMT_YES_NO.

<1> YES
<2> NO

<d> [illegible]2 1 [illegible] 7 . 1 2 :
<r> REFUSED

For peer review only

Household Health History

These questions ask about the health history of others, rather than yourself.

1. In the past 12 months, have you visited someone staying in a healthcare facility (e.g., hospital, nursing home, inpatient rehabilitation facility)?

- ○ Yes
- ○ No → **Go to question 4** HMH010 FMT_YES_NO.
- ○ Don't know → **Go to question 4**

2. In the past 12 months, did you provide help in caring for the person(s) while they were staying in the healthcare facility? *By help in caring, we mean having physical or hands-on contact with the person.*

○ Yes ○ No ○ Don't know HMH020 FMT_YES_NO.

3. In the past 12 months, about how many total days did you make a visit to someone who was staying in a healthcare facility (e.g., hospital, nursing home, inpatient rehabilitation facility)?

[|] total number of days HMH030 FMT_NUMERIC.

4. Has anyone in your household had an infection with a drug-resistant germ?

○ Yes ○ No ○ Don't know HMH040 FMT_YES_NO.

5. Has anyone in your household had an infection from a hospital or healthcare setting?

- ○ Yes → *Specify the infection(s) that they had below.* HMH050 FMT_YES_NO.
  [ ] HMH055 $FMT_CHAR.
- ○ No
- ○ Don't know

6. Have you ever visited a person who was placed in isolation while in a hospital, nursing home, or inpatient rehabilitation facility? *That is, you were required to wear at least a pair of gloves and a gown before seeing them.*

○ Yes ○ No ○ Don't know HMH060 FMT_YES_NO.

For peer review only - http://bmjopen.bmj.com/site/about/guidelines.xhtml

Your Health History

This next set of questions is about your health history.

1. At the time of your birth, were you delivered by Caesarean section?

- ◯ Yes
- ◯ No
- ◯ Don't know

HMI010 FMT_YES_NO.

2a. Were you breastfed as an infant?

- ◯ Yes
- ◯ No → **Go to question 3**
- ◯ Don't know

HMI020 FMT_YES_NO.

2b. For how long were you breastfed?

☐☐ months

- ◯ Don't know

HMI025 FMT_NUMERIC.

3. Have you ever had surgery on your digestive system (e.g. esophagus, stomach, liver, appendix, small and large intestines, gall bladder, and/or pancreas)?

- ◯ Yes
- ◯ No
- ◯ Don't know

HMI030 FMT_YES_NO.

4. In the <u>past 12 months</u>, have you had any of the following medical devices?
Fill in all that apply.

◯ Urinary catheter	HMI040_a	FMT_YES_NO.
◯ Vascular catheter	HMI040_b	FMT_YES_NO.
◯ Feeding tube	HMI040_c	FMT_YES_NO.
◯ Rectal tube	HMI040_d	FMT_YES_NO.
◯ Don't know	HMI040_e	FMT_YES_NO.

For peer review only - http://bmjopen.bmj.com/site/about/guidelines.xhtml

5. In the past 12 months, have you had dialysis treatment?

○ Yes
○ No HMI050 FMT_YES_NO.
○ Don't know

6a. Have you ever been a patient in a nursing home or inpatient rehabilitation facility?

○ Yes
○ No → **Go to question 7a, page 11** HMI060 FMT_YES_NO.
○ Don't know

6b. How many times were you a patient in a nursing home or inpatient rehabilitation facility?

☐☐ times HMI062 FMT_NUMERIC.

○ Don't know

6c. When was your most recent stay in a nursing home or inpatient facility? *Please tell us what month and year this visit began.*

☐☐ month HMI065_m FMT_NUMERIC.

☐☐☐☐ year HMI065_y FMT_NUMERIC.

6d. What was the approximate length of stay?

☐☐ days HMI068 FMT_NUMERIC.

For peer review only

7a. In the past year, have you taken an antibiotic *(a drug used to treat an infection)*?

- ○ Yes
- ○ No → **Go to question 8a**
- ○ Don't know → **Go to question 8a**

HMI070 FMT_YES_NO.

7b. In the table below, please list the name(s) of the antibiotics, the illness or condition for which you took them, and the length of time you took them. *If you were prescribed the same antibiotic more than once in the past year, list it multiple times.*

Name the antibiotic you took in the last year below:	*The reason (illness or condition) for taking the medication:*	*For how many days did you take this antibiotic?*
1. HMI071_a $FMT_CHAR.	HMI071_b $FMT_CHAR.	HMI071_c $FMT_CHAR.
2. HMI072_a $FMT_CHAR.	HMI072_b $FMT_CHAR.	HMI072_c $FMT_CHAR.
3. HMI073_a $FMT_CHAR.	HMI073_b $FMT_CHAR.	HMI073_c $FMT_CHAR.
4. HMI074_a $FMT_CHAR.	HMI074_b $FMT_CHAR.	HMI074_c $FMT_CHAR.
5. HMI075_a $FMT_CHAR.	HMI075_b $FMT_CHAR.	HMI075_c $FMT_CHAR.

☐ **If you have had more than five antibiotic prescriptions in the past year, please check this box.** HMI076 FMT_YES_NO.

For peer review only

8a. Are you currently using probiotic supplements? *Specifcally, we are referring to pills containing healthy bacteria.*

- ○ Yes
- ○ No → **Go to question 9, page 12**
- ○ Don't know → **Go to question 9, page 12**

HMI080 FMT_YES_NO.

8b. When was the last time you took the probiotic supplement?

○ Today or ☐☐ number of days ago HMI085 FMT_NUMERIC.

For peer review only - http://bmjopen.bmj.com/site/about/guidelines.xhtml

9. In the <u>past 12 months</u>, have you taken a proton pump inhibitor? *Proton pump inhibitors are drugs that suppress the production of acid in your stomach.* Some examples of trade (generic) names are: Aciphex® (rabeprazole), Protonix® (pantoprazole), Nexium® (esomeprazole), Prevacid® (lansoprazole), Kapidex® (dexlansoprazole), Zegerid® (omeprazole/sodium bicarbonate), Prilosec® (omeprazole), Dexilant® (dexlansoprazole).

- ◯ Yes
- ◯ No HMI090 FMT_YES_NO.
- ◯ Don't know

10. Has a doctor or other health care provider ever told you that you had an infection with a drug-resistant germ? *A germ is resistant when one or more drugs ordinarily used to treat an infection with that germ cannot kill it.*

- ◯ Yes → *Specify the infection(s) you had below.* HMI100 FMT_YES_NO.

  HMI105 $FMT_CHAR.
- ◯ No
- ◯ Don't know

11. Has a doctor or other health care provider ever told you that you got an infection from a hospital or health care setting?

- ◯ Yes → *Specify the infection(s) you had below.* HMI110 FMT_YES_NO.

  HMI115 $FMT_CHAR.
- ◯ No
- ◯ Don't know

12. Have you ever been put in isolation as a patient in a hospital, nursing home, or inpatient rehabilitation facility? *That is, visitors were required to wear at least a pair of gloves and gown before seeing you.*

- ◯ Yes
- ◯ No HMI120 FMT_YES_NO.
- ◯ Don't know

For peer review only - http://bmjopen.bmj.com/site/about/guidelines.xhtml

[illegible]

[illegible]

[illegible]

F O 1

- ◯ Before 1900
- ◯ 1901 to 1950
- ◯ 1951 to 1978
- ◯ 1979 to 1990
- ◯ 1991 and after
- ◯ Don't know

F O 1D R F O 0,

How long ha e you li ed at this address

F O 1

- ◯ 0-1 years
- ◯ 1-3 years
- ◯ 3-10 years
- ◯ >10 years

F O 1D R F O 0,

3 hat kind of pets do you keep inside your home now ***Fill in all that apply.***

F O03 0

- ◯ None
- ◯ Cat
- ◯ Dog
- ◯ Bird
- ◯ Hamster, mice, guinea pig, gerbils
- ◯ Reptile
- ◯ Fish
- ◯ Other

F O03 0	FMT_YES_NO.
HOQ250_R2_B	FMT_YES_NO.
HOQ250_R2_C	FMT_YES_NO.
HOQ250_R2_D	FMT_YES_NO.
HOQ250_R2_E	FMT_YES_NO.
HOQ250_R2_F	FMT_YES_NO.
HOQ250_R2_G	FMT_YES_NO.
HOQ250_R2_H	FMT_YES_NO.

Do you ha e a basement in this home

HOQ066_R2

- ◯ Yes
- ◯ No

HOQ066_R2 FMT_YES_NO.

For peer review only - http://bmjopen.bmj.com/site/about/guidelines.xhtml

[illegible]

St◯m radiators
- ◯ Hot water radiators / heaters
- ◯ Forced air system using gas or fuel oil
- ◯ Active solar
- ◯ Wood burning stoves
- ◯ Portable electric space heaters
- ◯ Other types of space heaters
- ◯ Other: *Print below.*
- ◯ Don't know

[illegible]

FMO.5.]P0
- ◯ Private well
- ◯ Community water supply → RWRHWR
- ◯ Don't know → RWRHWR

FMO.5.]P0DKR]FMO.5.]P0],

[illegible]

FMO.53]P0
- ◯ feet
- ◯ 50-99 feet
- ◯ 100-149 feet
- ◯ > 150 feet
- ◯ Don't know

FMO.53]P0DKR]FMO.53],

RREDKRPHDWHU OWHWHDWPHWWHPDWKRPHRDWHU

FMO.6.]P0
- ◯ Yes
- ◯ No → RWRHWRSDH
- ◯ Don't know → RWRHWRSDH

FMO.6.]P0DKR]WCQ]LM,

HDRWKHDWHUWHW HDWPHWWHPRKRPH *Fill in all that apply.*

FMO.61]P0
- ◯ [illegible]
- ◯ [illegible]
- ◯ Water softener
- ◯ Aerator
- ◯ Reverse osmosis
- ◯ None of these are in our home
- ◯ Other: *Print below.*
- ◯ Don't know

FMO.61]P0]? DKR]WCQ]LM,
FMO.61]P0]@ DKR]WCQ]LM,
FMO.61]P0]A DKR]WCQ]LM,
FMO.61]P0]B DKR]WCQ]LM,
FMO.61]P0]C DKR]WCQ]LM,
FMO.61]P0]D DKR]WCQ]LM,
FMO.61]P0]EDKR]WCQ]LM,
FMO.61]P0]MRFCP"DKR]AF?P,

For peer review only - http://bmjopen.bmj.com/site/about/guidelines.xhtml

7 ⊠ ⊠ ⊠ ⊠ ⊠ ⊠⊠ ⊠ ⊠ F ⊠ ⊠ ⊠ ⊠ ⊠ ⊠ ⊠ ⊠ ⊠ ⊠ ⊠ F ⊠ ⊠ P ⊠ F ⊠ O ⊠ ⊠ ⊠ F ⊠
⊠ P ⊠ 7 ⊠ ⊠ ⊠ ⊠ ⊠ ⊠ ⊠
P ⊠ P ⊠ ⊠ ⊠ ⊠ O ⊠ ⊠ ⊠ O ⊠ ⊠ ⊠ ⊠ ⊠ ⊠ F ⊠ P ⊠ ⊠ ⊠ ⊠

⊠⊠ ⊠ ⊠ ⊠ J ⊠ ⊠ ⊠ O ⊠ ⊠ ⊠ P ⊠ ⊠ ⊠ ⊠ ⊠ ⊠ ⊠ ⊠ ⊠ ⊠⊠ ⊠ ⊠ ⊠ ⊠
⊠⊠ ⊠ ⊠ ⊠ ⊠ ⊠ ⊠ ⊠ ⊠ ⊠ ⊠ ⊠ ⊠ ⊠ O ⊠ ⊠ ⊠ ⊠O ⊠ ⊠ ⊠ ⊠ ⊠ ⊠ ⊠ J ⊠ ⊠ ⊠ ⊠ O ⊠

- 0 times
- 1 time
- 2-3 times
- 4-10 times
- > 10 times
- Don't know

[illegible]

- 0 times → [illegible]
- 1 time
- 2-3 times
- 4-10 times
- > 10 times
- Don't know

[illegible] *Fill in all that apply.*

- Kitchen
- Bathroom
- Living room or family room
- Bedroom
- Laundry room
- Basement
- Other: *Print below.*
- Don't know

For peer review only - http://bmjopen.bmj.com/site/about/guidelines.xhtml

INSURANCE, ACCESS, UTILIZATION (IUQ)

IUQ010pre The next questions are about health insurance and your use of the health care system.

INTERVIEWER: HIT ENTER TO CONTINUE

IUQ010 During the last 12 months, how many months did you have health insurance?

(INTERVIEWER: IF NO INSURANCE DURING 12 PRECEDING MONTHS, ENTER "0".)

IUQ010 FMT_NUMERIC.

<0-12> **MONTHS** **(If 0, skip to IUQ012. If 12, skip to IUQ020_R2. Otherwise, go to IUQ015.)**

<d> DON'T KNOW **(Skip to IUQ015)**
<r> REFUSED **(Skip to IUQ015)**

IUQ012 If you wanted to, could you be covered by health insurance through a job or through a household family member's job? That is, do you or a household family member parent or spouse have an employer that offers health insurance?

IUQ012 FMT_IUQ012 B.

<1> EMPLOYER (EITHER YOURS OR FAMILY MEMBER'S) OFFERS HEALTH INSURANCE **(SKIP TO IUQ014)**
<2> EMPLOYER (EITHER YOURS OR FAMILY MEMBER'S) **DOES NOT** OFFER HEALTH INSURANCE **(GO TO IUQ013)**

<d> DON'T KNOW **(GO TO IUQ013)**
<r> REFUSED **(GO TO IUQ013)**

IUQ013 Did you consider purchasing individual health insurance through the new health care program, known as the "Affordable Care Act" or "Obamacare," that allows many individuals to purchase subsidized insurance through the Marketplace?

Would you say yes, but it was too expensive because you did not qualify for a subsidy, yes, but it was too expensive even with a subsidy, you were not eligible to purchase through the marketplace, or you did not consider purchasing coverage through the Marketplace?

(INTERVIEWER: HAND CARD)

IUQ013 FMT_IUQ013 B.

<1> YES, BUT IT WAS TOO EXPENSIVE BECAUSE I DID NOT QUALIFY FOR A SUBSIDY **(SKIP TO IUQ100)**
<2> YES, BUT IT WAS TOO EXPENSIVE EVEN WITH A SUBSIDY (**SKIP TO IUQ100)**
<3> I WAS NOT ELIGIBLE TO PURCHASE THROUGH THE MARKETPLACE **(SKIP TO IUQ100)**
<4> I DID NOT CONSIDER PURCHASING COVERAGE THROUGH THE MARKETPLACE **(SKIP TO IUQ100)**

For peer review only - http://bmjopen.bmj.com/site/about/guidelines.xhtml

INSURANCE, ACCESS, UTILIZATION (IUQ)

<d> DON'T KNOW **(SKIP TO IUQ100)**
<r> REFUSED **(SKIP TO IUQ100)**

IUQ014 Why don't you have health insurance coverage from that employer? You are not eligible for the health insurance plan, it is too expensive and you cannot afford the premiums, or you do not think it is worth it?

IUQ014 FMT_IUQ014 B.

<1> I AM NOT ELIGIBLE FOR THE HEALTH INSURANCE PLAN **(SKIP TO IUQ100)**
<2> IT IS TOO EXPENSIVE – CANNOT AFFORD THE PREMIUMS **(SKIP TO IUQ100)**
<3> I DO NOT THINK IT IS WORTH IT **(Skip to IUQ100)**

<d> DON'T KNOW **(SKIP TO IUQ100)**
<r> REFUSED **(SKIP TO IUQ100)**

IUQ015 FMT_YES_NO.

IUQ015 Do you currently have health insurance?

<1> YES **(GO TO IUQ020_R2)**
<2> NO **(GO TO IUQ020_R2)**

<d> DON'T KNOW **(SKIP TO IUQ100)**
<r> REFUSED **(SKIP TO IUQ100)**

IUQ020_R2 What kinds of health insurance or health care coverage do you have now, or did you have during the last 12 months? In answering this question, please EXCLUDE plans that pay for only one type of service, such as nursing home care, accidents, family planning, or dental care, and plans that only provide extra cash when hospitalized.

(INTERVIEWER: HAND CARD. ENTER ALL THAT APPLY.)

<1> EMPLOYER OR UNION SPONSORED PLAN **[GOTO IUQ021]**
<2> PRIVATE INDIVIDUALLY PURCHASED HEALTH PLAN **[GOTO IUQ025]**
<3> MEDICARE, FOR PEOPLE 65 OR OLDER OR PEOPLE WITIH CERTAIN DISABILITIES **[GOTO IUQ030]**

<5> MEDICAID, MEDICAL ASSISTANCE, MA, BADGER CARE, BADGER CARE PLUS **[GOTO IUQ030]**

<8> INDIAN HEALTH SERVICE **[GOTO IUQ030]**
<9> MILITARY CARE (TRICARE/VA/CHAMP-VA) **[GOTO IUQ030]**
<10> OTHER PLAN (SPECIFY) **[GOTO IUQ025]**

<d> DON'T KNOW **[goto IUQ025]**
<r> REFUSED **[goto IUQ025]**

For peer review only - http://bmjopen.bmj.com/site/about/guidelines.xhtml

INSURANCE, ACCESS, UTILIZATION (IUQ)

HIT “x” TO EXIT

**IUQ020_R2_A**	**FMT_IUQ020_R2_.**
**IUQ020_R2_B**	**FMT_IUQ020_R2_.**
**IUQ020_R2_C**	**FMT_IUQ020_R2_.**
**IUQ020_R2_D**	**FMT_IUQ020_R2_.**
**IUQ020_R2_E**	**FMT_IUQ020_R2_.**
**IUQ020_R2_F**	**FMT_IUQ020_R2_.**
**IUQ020_R2_G**	**FMT_IUQ020_R2_.**
**IUQ020_R2_H**	**FMT_IUQ020_R2_.**
**IUQ020_R2_I**	**FMT_IUQ020_R2_.**
**IUQ020_R2_J**	**FMT_IUQ020_R2_.**
**IUQ020_R2_OTHER**	**FMT_CHAR R2_.**

IUQ021 Do you get the Employer or Union Sponsored Plan coverage through your own job or from a family member’s insurance plan?

**IUQ021**	**FMT_IUQ021_.**

<1> YOUR OWN JOB OR EMPLOYER

<2> A FAMILY MEMBER’S JOB OR EMPLOYER
<3> OTHER

<d> DON’T KNOW
<r> REFUSED

[ALL RESPONSES AT IUQ021 GO TO IUQ023]

IUQ023 Was your job based coverage purchased through the SHOP, Small Business Health Options Program?

**IUQ023**	**FMT_YES_NO.**

<1> YES **(IF IUQ020_R2 = 2, 10, d, or r GO TO IUQ025, ELSE SKIP TO IUQ026)**

<2> NO **(IF IUQ020_R2 = 2, 10, d, or r GO TO IUQ025, ELSE SKIP TO IUQ030)**

<d> DON’T KNOW **(IF IUQ020_R2 = 2, 10, d, or r GO TO IUQ025, ELSE SKIP TO IUQ030)**
<r> REFUSED **(IF IUQ020_R2 = 2, 10, d, or r GO TO IUQ025, ELSE SKIP TO IUQ030)**

IUQ025 The next questions ask about the new health care program, known as the Affordable Care Act or “Obamacare.” As you may know, the health care law creates health insurance exchanges or marketplaces where people can shop for insurance on

For peer review only

INSURANCE, ACCESS, UTILIZATION (IUQ)

Healthcare.gov. Some people can get financial help in the form of a tax credit from the federal government to buy a health insurance policy through these marketplaces.

Did you or a family member buy your private health insurance plan from this Marketplace, healthcare.gov?

IUQ025 FMT_YES_NO.

<1> YES **(GO TO IUQ026)**

<2> NO **(SKIP TO IUQ030)**

<d> DON'T KNOW **(GO TO IUQ026)**
<r> REFUSED **(GO TO IUQ026)**

IUQ026 Do you know what kind of health plan you have? Bronze, silver, gold, platinum, catastrophic or are you not sure?

IUQ026 FMT_IUQ026_.

<1> BRONZE
<2> SILVER
<3> GOLD
<4> PLATINUM
<5> CATASTROPHIC
<6> NOT SURE

<d> DON'T KNOW
<r> REFUSED

IUQ027 Did you or your family member get a federal tax credit or subsidy to help with or reduce the costs of buying your health insurance plan?

<1> YES
<2> NO

IUQ027 FMT_YES_NO.

<d> DON'T KNOW
<r> REFUSED

IUQ030 Does your health insurance plan, including any supplemental coverage you might have, cover all of the costs, some of the costs, or none of the costs associated with prescription medications?

IUQ030 FMT_ALL_SOME_NONE.

<1> ALL
<2> SOME
<3> NONE **(SKIP TO IUQ040)**

<d> DON'T KNOW
<r> REFUSED

For peer review only

For peer review only - http://bmjopen.bmj.com/site/about/guidelines.xhtml

INSURANCE, ACCESS, UTILIZATION (IUQ)

IUQ035 Is this prescription drug coverage through your regular plan or through a supplemental insurance program for prescription drug coverage?

(INTERVIEWER: ENTER ALL THAT APPLY)

<1> REGULAR PLAN
<2> SUPPLEMENTAL, MEDICARE PART D
<3> SUPPLEMENTAL, WISCONSIN SENIOR CARE
<4> SUPPLEMENTAL, OTHER (SPECIFY)

<d> DON'T KNOW
<r> REFUSED

HIT 'x' TO EXIT

**IUQ035_A**	**FMT_IUQ035_.**
**IUQ035_B**	**FMT_IUQ035_.**
**IUQ035_C**	**FMT_IUQ035_.**
**IUQ035_D**	**FMT_IUQ035_.**
**IUQ035_OTHER**	**FMT_CHAR.**

IUQ040 Does your insurance plan cover all of the costs, some of the costs, or none of the costs associated with preventive dental services including oral exam, cleaning, sealant, etc.?

**IUQ040**	**FMT_ALL_SOME_NONE.**

<1> ALL **(SKIP TO IUQ050)**
<2> SOME **(GO TO IUQ044)**
<3> NONE **(GO TO IUQ044)**

<d> DON'T KNOW
<r> REFUSED

IUQ044 If your health insurance plan did not cover all of the costs, do you have a separate dental plan that pays for preventive services?

<1> YES
<2> NO

**IUQ044**	**FMT_YES_NO.**

<d> DON'T KNOW
<r> REFUSED

IUQ050 Does your health insurance plan cover all of the costs, some of the costs, or none of the costs associated with other preventive services for adults, like checkups, immunizations, and screenings?

<1> ALL
<2> SOME
<3> NONE

**IUQ050**	**FMT_ALL_SOME_NONE.**

<d> DON'T KNOW
<r> REFUSED

For peer review only

INSURANCE, ACCESS, UTILIZATION (IUQ)

IUQ070 Does your plan require you to sign up with a certain primary care doctor, group of doctors, or a certain clinic that you must go to for all of your routine care?

<1> YES
<2> NO

IUQ070 FMT_YES_NO.

<d> DON'T KNOW
<r> REFUSED

IUQ100 In the last 12 months, have you used the internet to seek information or advice on your health, or that of your family?

<1> YES
<2> NO

IUQ100 FMT_YES_NO.

<d> DON'T KNOW
<r> REFUSED

IUQ105 In the last 12 months, have you telephoned a health care professional to discuss a health problem or question related to yourself or your family?

<1> YES
<2> NO

IUQ105 FMT_YES_NO.

<d> DON'T KNOW
<r> REFUSED

IUQ110 In the last 12 months, have you emailed a health care professional to discuss a health problem or question related to yourself or your family?

<1> YES
<2> NO

IUQ110 FMT_YES_NO.

<d> DON'T KNOW
<r> REFUSED

IUQ115 How often do you need to have someone help you when you read instructions, pamphlets, or other written material from your doctor or pharmacy?

Would you say never, rarely, sometimes, often or always?

<1> NEVER
<2> RARELY
<3> SOMETIMES
<4> OFTEN
<5> ALWAYS

IUQ115 FMT_FREQ_IUQ115_.

<d> DON'T KNOW
<r> REFUSED

For peer review only

INSURANCE, ACCESS, UTILIZATION (IUQ)

IUQ120 Do you have a usual place where you go when you feel sick or need advice about your health?

(INTERVIEWER: HAND CARD)

**IUQ120**	**FMT_IUQ120_.**
**IUQ120_OTHER**	**FMT_CHAR.**

<1> YES, I USUALLY GO TO A HOSPITAL EMERGENCY ROOM
<2> YES, I USUALLY GO TO A HOSPITAL OUTPATIENT DEPARTMENT
<3> YES, I USUALLY GO TO A CLINIC OR DOCTOR'S OFFICE
<4> YES, I USUALLY GO TO A COMMUNITY HEALTH CENTER
<5> YES, I USUALLY GO TO SOME OTHER PLACE (SPECIFY)
<6> NO, I DON'T HAVE A USUAL PLACE OF CARE **(Skip to IUQ140)**

<d> DON'T KNOW
<r> REFUSED

IUQ125 What is the name of the health facility you usually go to when you feel sick or need advice about your health and on what streets in what town/city is this facility located?

NAME: ______________________________

STREET ON WHICH THE FACILITY IS LOCATED: ______________________

NEAREST INTERSECTING OR CROSS STREET: ______________________

TOWN/CITY: ______________________________

<d> DON'T KNOW
<r> REFUSED

**IUQ125_A**	**FMT_CHAR.**
**IUQ125_C**	**FMT_CHAR.**
**IUQ125_D**	**FMT_CHAR.**
**IUQ125_B**	**FMT_CHAR.**

IUQ128 When you go to this health facility, do you usually see a general doctor, a specialist doctor, a nurse practitioner or physician assistant or someone else?

<1> GENERAL DOCTOR
<2> SPECIALIST DOCTOR
<3> NURSE PRACTITIONER/PHYSICIAN ASSISTANT
<4> SOMEONE ELSE

<d> DON'T KNOW
<r> REFUSED

**IUQ128**	**FMT_IUQ128_.**

IUQ130 When you go to this health facility ,do you usually see the same health care provider?

<1> YES
<2> NO **(SKIP TO IUQ140)**

**IUQ130**	**FMT_YES_NO.**

<d> DON'T KNOW
<r> REFUSED

For peer review only - http://bmjopen.bmj.com/site/about/guidelines.xhtml

INSURANCE, ACCESS, UTILIZATION (IUQ)

IUQ137 What is the specialty of the health care provider you usually see?

<1> INTERNAL MEDICINE
.
<2> FAMILY PRACTICE

<3> OBSTETRICS/GYNECOLOGY
<4> OTHER SPECIALIST (SPECIFY)

<d> DON'T KNOW
<r> REFUSED

**IUQ137**	**FMT_IUQ137 B.**
**IUQ137_OTHER**	**FMT_CHAR.**

IUQ140 Sometimes people take fewer medicines than their health care provider prescribed, or they don't have their prescription filled right away.

At any time during the last 12 months, have you taken less medicine than your doctor prescribed or not had your prescription filled **because of the cost**?

<1> YES
<2> NO

<d> DON'T KNOW
<r> REFUSED

**IUQ140**	**FMT_YES_NO.**

IUQ170 In the last 12 months, how many different **times** have you seen a mental health professional such as a psychologist, psychiatrist, counselor, or psychiatric nurse about a personal problem or a problem with alcohol or drugs?

<0-76> TIMES DURING PREVIOUS YEAR

<d> DON'T KNOW
<r> REFUSED

**IUQ170**	**FMT_NUMERIC.**

IUQ180 In the last 12 months, how many different **times** did you go to a hospital emergency room for medical treatment for yourself?

<0-76> TIMES DURING PREVIOUS YEAR

<d> DON'T KNOW
<r> REFUSED

**IUQ180**	**FMT_NUMERIC.**

IUQ190 In the last 12 months, how many different **times** were you a patient in a hospital for at least one night or longer?

<0> NO TIMES **(skip to IUQ220)**
<1-76> TIMES DURING PREVIOUS YEAR

<d> DON'T KNOW **(skip to IUQ220)**

**IUQ190**	**FMT_NUMERIC.**

For peer review only

For peer review only - http://bmjopen.bmj.com/site/about/guidelines.xhtml

INSURANCE, ACCESS, UTILIZATION (IUQ)

<r> REFUSED **(skip to IUQ220)**

IUQ192 For each time you were in the hospital in the last 12 months, for how many days did you stay in the hospital? Begin with your most recent hospital stay and tell us about your stay for up to 5 hospital stays.

{stay 1} <1-30> DURATION

**IUQ192_S1**	**FMT_NUMCAT.**
**IUQ192_S2**	**FMT_NUMCAT.**
**IUQ192_S3**	**FMT_NUMCAT.**
**IUQ192_S4**	**FMT_NUMCAT.**
**IUQ192_S5**	**FMT_NUMCAT.**

UNITS
{unit 1} <1> DAYS
<2> MONTHS

**IUQ192_U1**	**FMT_DAYS_MONTHS.**
**IUQ192_U2**	**FMT_DAYS_MONTHS.**
**IUQ192_U3**	**FMT_DAYS_MONTHS.**
**IUQ192_U4**	**FMT_DAYS_MONTHS.**
**IUQ192_U5**	**FMT_DAYS_MONTHS.**

<d> DON'T KNOW
<r> REFUSED

STAY #1 @s1 DURATION @u1 UNIT (Will display number given in IUQ190)
STAY #2 @s2 DURATION @u2 UNIT
STAY #3 @s3 DURATION @u3 UNIT
STAY #4 @s4 DURATION @u4 UNIT
STAY #5 @s5 DURATION @u5 UNIT

IUQ194 For any of your hospitalizations in the last 12 months, how many times [endif]were you in an intensive care unit?

<0> NO
<1> YES <1-[fill IUQ190]> TIMES **[maximum is number of hospitalizations in IUQ190**]

**IUQ194**	**FMT_NUMCAT.**

<d> DON'T KNOW
<r> REFUSED

IUQ200 How would you rate the quality of the care you received when you were most recently a patient in a hospital for at least one night or longer **during the last year?**

Would you say it was excellent, very good, good, fair or poor?

<1> EXCELLENT
<2> VERY GOOD
<3> GOOD
<4> FAIR
<5> POOR

**IUQ200**	**FMT_EVGGFP.**

<d> DON'T KNOW
<r> REFUSED

IUQ220 How long has it been since you last saw a doctor or health care provider for a

For peer review only - http://bmjopen.bmj.com/site/about/guidelines.xhtml

INSURANCE, ACCESS, UTILIZATION (IUQ)

routine physical exam, check-up or screening procedure?

<0> NEVER **(SKIP TO IUQ260pre)**
<1-76> ENTER NUMBER **(Go to IUQ230)**

IUQ220_N FMT_NUMCAT.
IUQ220_U FMT_FREQ.

<d> DON'T KNOW **(Go to IUQ225)**
<r> REFUSED **(Skip to IUQ230)**

<1> DAYS **(Skip to IUQ230)**
<2> WEEKS **(Skip to IUQ230)**
<3> MONTHS **(Skip to IUQ230)**
<4> YEARS **(If more than 1 year, skip to IUQ260pre. Otherwise skip to IUQ230)**

IUQ225 Has it been never, 6 months or less, more than 6 months but no more than 1 year ago, more than 1 year ago but no more than 3 years ago or more than 3 years ago?

IUQ225 FMT_IUQ225_255_.

<1> NEVER **(Skip to IUQ260pre)**
<2> 6 MONTHS OR LESS **(Go to IUQ230)**
<3> MORE THAN 6 MONTHS BUT NO MORE THAN 1 YEAR AGO **(GO TO IUQ230)**
<4> MORE THAN 1 YEAR BUT NO MORE THAN 3 YEARS AGO **(Skip to IUQ260pre)**
<5> MORE THAN 3 YEARS AGO **(Skip to IUQ260pre)**

<d> DON'T KNOW **(Skip to IUQ260pre)**
<r> REFUSED **(Skip to IUQ260pre)**

IUQ230 How would you rate the quality of the care you received when you last saw a doctor or health care provider for a routine physical exam, check-up, or screening procedure during the last year?

IUQ230 FMT_EVGGFP.

Would you say it was excellent, very good, good, fair or poor?

<1> EXCELLENT
<2> VERY GOOD
<3> GOOD
<4> FAIR
<5> POOR

<d> DON'T KNOW
<r> REFUSED

IUQ260pre The next questions are about your **overall** level of satisfaction with quality and access to health care.

For peer review only - http://bmjopen.bmj.com/site/about/guidelines.xhtml

INSURANCE, ACCESS, UTILIZATION (IUQ)

INTERVIEWER: HIT ENTER TO CONTINUE

IUQ260_R2 Sometimes people have problems getting health care when they need it. During the last 12 months, was there any time that you felt that you needed medical care or surgery but did not get it?

IUQ260_R2 FMT_YES_NO.

<1> YES **(Go to IUQ265)**
<2> NO **(Skip to IUQ270)**

<d> DON'T KNOW **(Skip to IUQ270)**
<r> REFUSED **(Skip to IUQ270)**

IUQ265 What was the main reason you didn't get the health care you needed?

IUQ265 FMT_IUQ265_.
IUQ265_OTHER $FMT_CHAR.

(INTERVIEWER: HAND CARD)

<1> I couldn't afford health care
<2> My insurance company wouldn't approve, cover or pay for care
<3> My insurance company required a referral but I couldn't get one
<4> The doctor (or clinic) refused to accept my insurance plan
<5> Medical care was too far away
<6> It was too expensive to get to health care
<7> I couldn't get there when the doctor's office was open
<8> It took too long to get an appointment
<9> I couldn't get through on the telephone to make an appointment
<10> The waiting list was too long
<11> Other (Specify)

<d> DON'T KNOW
<r> REFUSED

IUQ270 In the past 12 months, did you experience **delay** in obtaining any type of health care?

IUQ270 FMT_YES_NO.

<1> YES
<2> NO **(Skip to IUQ280)**

<d> DON'T KNOW **(Skip to IUQ280)**
<r> REFUSED **(Skip to IUQ280)**

IUQ275 What was the main reason for the difficulty or delay in obtaining health care?

(INTERVIEWER: HAND CARD)

IUQ275 FMT_IUQ275_.

<1> I couldn't afford health care
<2> My insurance company wouldn't approve, cover or pay for care
<3> My insurance company required a referral but I couldn't get one
<4> The doctor refused to accept my insurance plan

For peer review only - http://bmjopen.bmj.com/site/about/guidelines.xhtml

INSURANCE, ACCESS, UTILIZATION (IUQ)

<5> Medical care was too far away
<6> It was too expensive to get to health care
<7> I couldn’t get there when the doctor’s office was open
<8> It took too long to get an appointment
<9> I couldn’t get through on the telephone to make an appointment
<10> The waiting list was too long
<11> Other (Specify)

<d> DON’T KNOW
<r> REFUSED

IUQ280 Overall, how would you rate the quality of the health care you received during the last 12 months?

IUQ280 FMT_EVGGFP.

(INTERVIEWER: HAND CARD)

Would you say it was excellent, very good, good, fair, poor or you did not receive any care?

<1> EXCELLENT
<2> VERY GOOD
<3> GOOD
<4> FAIR
<5> POOR
<6> NOT APPLICABLE (DID NOT RECEIVE ANY CARE)

<d> DON’T KNOW
<r> REFUSED

IUQ290 Overall, how satisfied were you with **the way** health care services were provided during the last 12 months?

IUQ290 FMT_SATIS_5CAT.

(INTERVIEWER: HAND CARD)

Were you very satisfied, somewhat satisfied, neither satisfied nor dissatisfied, somewhat dissatisfied, very dissatisfied or you did not receive any care?

<1> VERY SATISFIED
<2> SOMEWHAT SATISFIED
<3> NEITHER SATISFIED NOR DISSATISFIED
<4> SOMEWHAT DISSATISFIED
<5> VERY DISSATISFIED
<6> NOT APPLICABLE (DID NOT RECEIVE ANY CARE)

<d> DON’T KNOW
<r> REFUSED

IUQ300 I want to hear you read as many words as you can from this list. Begin with the first word and read aloud. When you come to a word you cannot read, do the best you can or say, 'blank' and go onto the next word.

For peer review only

INSURANCE, ACCESS, UTILIZATION (IUQ)

IUQ300 FMT_NUMERIC.

INTERVIEWER: HAND CARD AND RECORD THE NUMBER OF CORRECT PRONUNCIATIONS

IF THE RESPONDENT TAKES MORE THAN FIVE SECONDS ON A WORD, POINT TO THE NEXT WORD, IF NECESSARY, TO MOVE THE SUBJECT ALONG. IF THE SUBJECT BEGINS TO MISS EVERY WORD, HAVE HIM OR HER PRONOUNCE ONLY KNOWN WORDS.

Menopause

Antibiotics

Exercise

Jaundice

Rectal

Anemia

Behavior

<0-7> CORRECT PRONUNCIATIONS

<d> DON'T KNOW
<r> REFUSED

For peer review only

Laboratory Tests (LAB)

[CONSENT QUESTIONS HAVE BEEN REMOVED. SEE CONS2 CODEBOOK.]

LABdate Date of Lab: **LABDATE DATE.**
<d> Don’t know <r> Refused

Date:
MM/DD/YYYY

[PARTICIPATION VARIABLES ARE NOT INCLUDED IN CODEBOOKS]

PART010 Was this an in-home appointment?

<1> YES **(Skip to LAB020)**
<2> NO **(go to PART020)**

PART020 Is the participant a confirmed driver who needs to be reimbursed for mileage to the appointment?
<1> YES **(go to PART030)**
<2> NO **(Skip to PART040)**

PART030 What was the round trip mileage to the sample collection site?
<0-99> ENTER NUMBER OF MILES

PART040 Does the participant need to be compensated for childcare?
<1> ONE CHILD ($12)
<2> TWO CHILDREN ($15)
<3> THREE OR MORE CHILDREN ($19.50)
<4> NO COMPENSATION REQUIRED

PART050 Does the participant need to be reimbursed for expenses like a bus, parking, or a taxi?
<1> YES, FOR A BUS **(go to PART060)**
<2> YES, FOR PARKING **(go to PART060)**
<3> YES, FOR A TAXI **(go to PART060)**
<4> NO **(Skip to LAB010)**

PART060 What was the total of the other expenses?
<0.00-99.00> ENTER NUMBER OF DOLLARS

LAB020 Phlebotomist Numbers:

<0-9999>

(ENTER ALL THAT APPLY, ENTER ‘x’ WHEN DONE)

LAB030 Processor Numbers:
<0-9999>

(ENTER ALL THAT APPLY, ENTER ‘x’ WHEN DONE)

For peer review only

Laboratory Tests (LAB)

LAB300 Did the participant attempt to donate any samples for the Wisconsin Microbiome Study?

<1> YES **(go to LAB310)**
<2> NO **(Skip to LAB410)** **LAB300 FMT_YES_NO.**

LAB310 Type: **Nasal Swab**
Status: **LAB310_S FMT_LAB_STATUS.**
<d> DONE
<f> FAILED **[goto LAB320]**
<r> REFUSED **[goto LAB320]**
<na> NOT ATTEMPTED **[goto LAB320]**

Collection Time: (Military time) **LAB310_C HHMM5.**
HH:MM

Refrigeration Time: (Military time) **LAB310_R HHMM5.**
HH:MM

LAB320 Type: **Oropharyngeal Swab**
Status: **LAB320_S FMT_LAB_STATUS.**
<d> DONE
<f> FAILED **[goto LAB330]**
<r> REFUSED **[goto LAB330]**
<na> NOT ATTEMPTED **[goto LAB330]**

Collection Time: (Military time) **LAB320_C HHMM5.**
HH:MM

Refrigeration Time: (Military time) **LAB320_R HHMM5.**
HH:MM

LAB330 Type: **Saliva Cup**
Status: **LAB330_S FMT_LAB_STATUS.**
<d> DONE
<f> FAILED **[goto LAB340]**
<r> REFUSED **[goto LAB340]**
<na> NOT ATTEMPTED **[goto LAB340]**

Collection Time: (Military time) **LAB330_C HHMM5.**
HH:MM

Refrigeration Time: (Military time) **LAB330_R HHMM5.**
HH:MM

LAB340 Type: **Axilla/Groin Swab**
Status: **LAB340_S FMT_LAB_STATUS.**

For peer review only

Laboratory Tests (LAB)

<d> DONE
<f> FAILED **[goto LAB341]**
<r> REFUSED **[goto LAB341]**
<na> NOT ATTEMPTED **[goto LAB341]**

Collection Time: (Military time) **LAB340_C HHMM5.**
HH:MM

Refrigeration Time: (Military time) **LAB340_R HHMM5.**
HH:MM

LAB341 What under-arm product do you most often use?

<1> DEODORANT ALONE
<2> ANTIPERSPIRANT ALONE
<3> DEODORANT/ANTIPERSPIRANT COMBINATION
<4> I USE NO PRODUCTS UNDER MY ARM [GO TO LAB350]
<5> OTHER PRODUCT, PLEASE SPECIFY

____

<d> DON'T KNOW <r> REFUSED **LAB341 FMT_LAB341_.**

LAB342 How often do you use the product above?

<1> LESS THAN ONCE A MONTH
<2> A FEW TIMES A MONTH
<3> A FEW TIMES A WEEK
<4> EVERY DAY

<d> DON'T KNOW <r> REFUSED **LAB342 FMT_LAB342_.**

LAB350 **Stool Sample**
Status: **LAB350 FMT_LAB_STATUS.**
<d> DONE
<i> INCOMPLETE – GAVE SHIPPER **(skip to LAB410)**
<r> REFUSED **(skip to LAB410)**

LAB360 When was the stool sample produced?
<d> Don't know <r> Refused
Date: **LAB360_DATE DATE.**
MM/DD/YYYY

Time: (Military time) **LAB360_TIME HHMM5.**
HH:MM

LAB370 When was the stool sample first refrigerated?
<d> Don't know <r> Refused

For peer review only - http://bmjopen.bmj.com/site/about/guidelines.xhtml

Laboratory Tests (LAB)

Date: MM/DD/YYYY — **LAB370_DATE DATE.**

Time: (Military time) HH:MM — **LAB370_TIME HHMM5.**

LAB380 When was sample received by phlebotomist:

Date: MM/DD/YYYY — **LAB380_DATE DATE.**

Time: (Military time) HH:MM — **LAB380_TIME HHMM5.**

LAB390 Time placed in refrigerator or cooler: (Military time) HH:MM — **LAB390_TIME HHMM5.**

LAB400 When were samples removed from cooler and mailed?

Date: MM/DD/YYYY — **LAB400_DATE DATE.**

Time: (Military time) HH:MM — **LAB400_TIME HHMM5.**

LAB410 Did the subject attempt to donate any samples for SHOW Core?

<1> YES **(Go to LAB010)**
<2> NO **(Skip to 250)** — **LAB410 FMT_YES_NO.**

LAB010 Check for any of the follow that restricted your choice of arm/vein:
(CHECK ALL THAT APPLY, ENTER ‘x’ WHEN DONE)

<1>	Mastectomy	**LAB010_A**	**FMT_LAB010_.**
<2>	Hematoma	**LAB010_B**	**FMT_LAB010_.**
<3>	Burns, Scars, Tattoos	**LAB010_C**	**FMT_LAB010_.**
<4>	Damaged veins	**LAB010_D**	**FMT_LAB010_.**
<5>	Shunt, Fistula or Graft	**LAB010_E**	**FMT_LAB010_.**
<6>	Recent IV	**LAB010_F**	**FMT_LAB010_.**
<7>	Caste	**LAB010_G**	**FMT_LAB010_.**
<8>	Edema	**LAB010_H**	**FMT_LAB010_.**
<9>	Obesity	**LAB010_I**	**FMT_LAB010_.**
<10>	Skin sores	**LAB010_J**	**FMT_LAB010_.**

LAB040

When was the last time you ate or drank anything other than plain water?

[INTERVIEWER: THIS QUESTION ELICITS THE LAST TIME THE SP ATE OR DRANK ANYTHING AND DETERMINES FASTING TIME. SPS ARE ALLOWED

For peer review only - http://bmjopen.bmj.com/site/about/guidelines.xhtml

Laboratory Tests (LAB)

TO CONSUME DIET SODA, BLACK COFFEE, OR TEA WITH ARTIFICIAL SWEETENERS LIKE SWEET'N LOW, NUTRASWEET, EQUAL, OR SPLENDA SINCE THESE HAVE NO EFFECT ON STUDY ANALYTES.

PARTICIPANTS ARE NOT ALLOWED TO HAVE CONSUMED: COFEE OR TEA WITH CREAM OR SUGAR, FLAVORED WATER, ALCOHOL, GUM, MINTS, LOZENGES, COUGH DROPS, COLD REMEDIES, ANTACIDS, ANTI-DIARRHEALS, LAXATIVES, OR DIETARY SUPPLEMENTS SUCH AS VITAMINS AND MINERALS.]

Last Ate:
<d> Don't know <r> Refused

Date: **LAB040_DATE DATE**
MM/DD/YYYY

Time: (Military time) **LAB040_TIME HHMM5.**
HH:MM

LAB050 1ST Draw Attempt TIME
(Military time)
<d> Don't know <r> Refused

HH:MM

Draw Time: **LAB050 HHMM5.**
HH:MM

LAB060 Type: **5mL SST Gold top for ML**

ML Label**:** [ALLOW 9 CHARACTERS]

Status: **LAB060 FMT_LAB_STATUS.**

<d> DONE
<f> FAILED
<p> PARTIAL
<r> REFUSED
<na> NOT ATTEMPTED

QC: <1> Checked <2> Unchecked

Comments?
<1> Enter Comments
<2> No Comments

For peer review only - http://bmjopen.bmj.com/site/about/guidelines.xhtml

For peer review only

Laboratory Tests (LAB)

LAB070 Type: **10mL Redtop for Repository 1**

SPID Label **(SCAN):**

Status: **LAB070 FMT_LAB_STATUS.**
<d> DONE
<f> FAILED
<p> PARTIAL
<r> REFUSED
<na> NOT ATTEMPTED

QC: <1> Checked <2> Unchecked

Comments?
<1> Enter Comments
<2> No Comments

LAB080 Type: **10mL Redtop for Repository 2**

SPID Label **(SCAN): [FILL FROM LAB070]**

Status: **LAB080 FMT_LAB_STATUS.**
<d> DONE
<f> FAILED
<p> PARTIAL
<r> REFUSED
<na> NOT ATTEMPTED

QC: <1> Checked <2> Unchecked

Comments?
<1> Enter Comments
<2> No Comments

LAB090 Type: **10 mL Lavender 1**

SPID Label **(SCAN): [FILL FROM LAB070]**

Status: **LAB090 FMT_LAB_STATUS.**
<d> DONE
<f> FAILED
<p> PARTIAL
<r> REFUSED
<na> NOT ATTEMPTED

NO DNA: <1> Checked <2> Unchecked

QC: <1> Checked <2> Unchecked

For peer review only

Laboratory Tests (LAB)

Comments?
<1> Enter Comments
<2> No Comments

LAB100 Type: **10 mL Lavender 2**

SPID Label **(SCAN): [FILL FROM LAB070]**

Status: **LAB100 FMT_LAB_STATUS.**
<d> DONE
<f> FAILED
<p> PARTIAL
<r> REFUSED
<na> NOT ATTEMPTED

NO DNA: <1> Checked <2> Unchecked

QC: <1> Checked <2> Unchecked

Comments?
<1> Enter Comments
<2> No Comments

LAB110 Type: **3 mL Lavender for ML 1**

ML Label:

Status: **LAB110 FMT_LAB_STATUS.**
<d> DONE
<f> FAILED
<p> PARTIAL
<r> REFUSED
<na> NOT ATTEMPTED

QC: <1> Checked <2> Unchecked

Comments?
<1> Enter Comments
<2> No Comments

LAB120 Type: **3 mL Lavender for ML 2**

ML Label:

For peer review only - http://bmjopen.bmj.com/site/about/guidelines.xhtml

Laboratory Tests (LAB)

Status: **LAB120 FMT_LAB_STATUS.**
<d> DONE
<f> FAILED
<p> PARTIAL
<r> REFUSED
<na> NOT ATTEMPTED

QC: <1> Checked <2> Unchecked

Comments?
<1> Enter Comments
<2> No Comments

LAB125 Type: **2.5 mL PaxGene Redtop for Biorepository**

SPID Label: **[FILL FROM LAB070]**

Status: **LAB125 FMT_LAB_STATUS.**
<d> DONE
<f> FAILED
<r> REFUSED
<na> NOT ATTEMPTED

QC: <1> Checked <2> Unchecked

Comments?
<1> Enter Comments
<2> No Comments

LAB130 **Number of Attempted Sticks**
Status: **LAB130 FMT_NUMERIC.**
<1-10> ATTEMPTS
<f> FAILED
<r> REFUSED
<na> NOT ATTEMPTED

Comments?
<1> Enter Comments
<2> No Comments

LAB140 END DRAW TIME **LAB140 HHMM5.**
(Military time)

<d> Don’t know <r> Refused

HH:MM

For peer review only

Laboratory Tests (LAB)

LAB150	Plasma Centrifuge Start Time:<br>(Military time)<br><br><d> Don't know <r> Refused<br><br>Blood: HH:MM	**LAB150 HHMM5.**
LAB155	Serum Centrifuge Start Time:<br>(Military time)<br><br><d> Don't know <r> Refused<br><br>HH:MM	**LAB155 HHMM5.**
LAB160	Number of plasma vials:<br><br><0-30> CRYOVIALS<br><br><d> Don't know <r> Refused	**LAB160 FMT_NUMERIC.**
LAB170	Number of serum vials:<br><br><0-30> CRYOVIALS<br><br><d> Don't know <r> Refused	**LAB170 FMT_NUMERIC.**
LAB180	Plasma Freezer Entry Time<br>(Military time)<br><br><d> Don't know <r> Refused<br><br>HH:MM	**LAB180 HHMM5.**
LAB185	Serum Freezer Entry Time<br><br>(Military time)<br><br><d> Don't know <r> Refused<br><br>HH:MM	**LAB185 HHMM5.**
LAB200	Type: **Urine Sample**<br><br>SPID Label: **[FILL FROM LAB070]**	

For peer review only

Laboratory Tests (LAB)

Status: **LAB200 FMT_LAB_STATUS.**
<1> DONE

<7> REFUSED **(goto LAB235)**
<8> FAILED **(goto LAB235)**
<9> NOT ATTEMPTED **(goto LAB235)**

Comments?
<1> Enter Comments
<2> No Comments

LAB190 Urine Sample Collection time (Military time) **LAB190 HHMM5.**

<d> Don't know <r> Refused

HH:MM

Urine Sample Centrifuge Time **LAB191 HHMM5.**
HH:MM

LAB210 Urine Sample **LAB210 FMT_NUMERIC.**

<0-50> mL of urine centrifuged

<d> Don't know
<r> Refused

LAB220 Number of urine vials: **LAB220 FMT_NUMERIC.**

<0-30> CRYOVIALS

<d> Don't know <r> Refused

QC: <1> Checked <2> Unchecked

LAB230 Urine Freezer Time (Military time) **LAB230 HHMM5.**

HH:MM

<d> Don't know <r> Refused

LAB235 BLOOD SPOTS
SPID Label:

For peer review only - http://bmjopen.bmj.com/site/about/guidelines.xhtml

Laboratory Tests (LAB)

Blood Spot Status: **LAB235 FMT_LAB_STATUS.**
<d> DONE <r> REFUSED (Skips to LAB240)
<f> FAILED (skips toLAB240) <na> NOT ATTEMPTED-BLOOD DRAW COMPLETED (Skip to LAB240)

Blood Spot Collection Time **LAB235_TIM1 HHMM5.**
(Military time)

HH:MM

Blood Spot Freezer Time **LAB235_TIM2 HHMM5.**
(Military time)

HH:MM

Number of spots completed on card
<0-8> **LAB235_NBS FMT_NUMERIC.**
<d> Don't know <r> Refused

LAB236 Blood Spots Comments?
<1> Enter Comments
<2> No Comments

LAB240 Saliva Sample
SPID Label: **[FILL FROM LAB070]**

Saliva Status: **LAB240_A FMT_LAB_STATUS.**
<d> DONE <r> REFUSED
<f> FAILED <na> NOT ATTEMPTED-BLOOD DRAW COMPLETED

Saliva Collection Time **LAB240 HHMM5.**
(Military time)

HH:MM

Saliva Freezer Time **LAB241 HHMM5.**
(Military time)

HH:MM

QC: <1> Checked <2> Unchecked

LAB250 Problems/Comments **LAB250 $FMT_CHAR.**
<1> Enter Comments

For peer review only - http://bmjopen.bmj.com/site/about/guidelines.xhtml

Laboratory Tests (LAB)

<2> No Comments

PART160 Was the 24 Hour Dietary Recall completed?
<1> YES
<2> NO, REFUSED
<3> PENDING PARTICIPATION

For peer review only

OCCUPATION (OCQ)

OCQ1pre In this part of the survey I will ask you questions about your work experience

INTERVIEWER: HIT ENTER TO CONTINUE

OCQ100 Which of the following were you doing **last week?**

(INTERVIEWER: HAND CARD.)

<1> Working at a job or business **(Skip to OCQ125)**
<2> With a job or business but not at work (for example, on vacation or sick) **(Skip to OCQ120)**
<3> Not working but looking for work **(Go on to OCQ110)**
<4> Not working at a job or business and not looking for a job **(Go on to OCQ110)**

<d> DON'T KNOW **(Skip to PAQ200pre)**
<r> REFUSED **(Skip to PAQ200pre)**

**OCQ100**	**FMT_OCQ100_.**

OCQ110 What is the main reason you are not in the paid workforce?

<1> TAKING CARE OF HOUSE OR FAMILY
<2> GOING TO SCHOOL
<3> RETIRED
<4> UNABLE TO WORK FOR HEALTH REASONS
<5> ON LAYOFF
<6> DISABLED
<7> OTHER (INTERVIEWER: SPECIFY RESPONSE FOLLOWED BY //)

<d> DON'T KNOW
<r> REFUSED

**OCQ110**	**FMT_OCQ110.**
**OCQ110_OTHER**	**$FMT_CHAR.**

OCQ115 How long have you been out of the paid workforce?

<1-76> ENTER NUMBER OF WEEKS, MONTHS, OR YEARS
<666> NEVER WORKED FOR PAY

<d> DON'T KNOW
<r> REFUSED

**OCQ115_N**	**FMT_NUMCAT.**

<2> WEEKS
<3> MONTHS

For peer review only - http://bmjopen.bmj.com/site/about/guidelines.xhtml

OCCUPATION (OCQ)

<4> YEARS

OCQ115_U	FMT_FREQ.

[SKIP TO PAQ200pre AFTER OCQ115]

OCQ120 What is the **main** reason you did not work **last week**?

<1> VACATION / LEAVE
<2> SICK OR TAKING CARE OF SICK FAMILY MEMBER
<3> CHILDCARE PROBLEMS
<4> EMPLOYER DID NOT SCHEDULE ME
<4> OTHER (INTERVIEWER: SPECIFY RESPONSE FOLLOWE D BY //)

<d> DON'T KNOW
<r> REFUSED

OCQ120	FMT_OCQ120_.
OCQ120_OTHER	$FMT_CHAR.

OCQ122 Do you **usually** work 35 hours or more per week in total at all jobs or businesses?

<1> YES
<2> NO

<d> DON'T KNOW
<r> REFUSED

OCQ122	FMT_YES_NO.

[SKIP TO OCQ130p AFTER OCQ122]

OCQ125 How many hours did you work **last week** at **all** jobs or businesses?

<1-168> HOURS WORKED LAST WEEK

<d> DON'T KNOW
<r> REFUSED

OCQ125	FMT_NUMERIC.

OCQ127 Do you **usually** work 35 hours or more per week in total at all jobs or businesses?

<1> YES
<2> NO

<d> DON'T KNOW
<r> REFUSED

OCQ127	FMT_YES_NO.

For peer review only - http://bmjopen.bmj.com/site/about/guidelines.xhtml

For peer review only

OCCUPATION (OCQ)

OCQ130p I'm going to ask you some questions about your currently held job. If you have more than one job, please answer these questions thinking only of the job which is the primary source of your income.

INTERVIEWER: HIT ENTER TO CONTINUE

OCQ130 What kind of work do you do for pay?

(INTERVIEWER: DO NOT PROBE. ENTER JOB TITLE OR WHATEVER THEY TELL YOU.)

<1> ENTER RESPONSE FOLLOWED BY //

<d> DON'T KNOW
<r> REFUSED

**OCQ130**	**FMT_OCQ_TEXTCODE.**
**OCQ130_TEXT**	**$FMT_CHAR.**

OCQ140 What are your most important activities or duties on this job or business?

(For example: sells cars, keeps account books, operates printing press, finished concrete.)

(INTERVIEWER: USE ACTIVE PROBES AS NECESSARY TO GET A CLEAR PICTURE OF WHAT THEY DO ON THEIR JOB.)

<1> ENTER RESPONSE FOLLOWED BY //

<d> DON'T KNOW
<r> REFUSED

**OCQ140**	**FMT_OCQ_TEXTCODE.**
**OCQ140_TEXT**	**$FMT_CHAR.**

For peer review only - http://bmjopen.bmj.com/site/about/guidelines.xhtml

OCCUPATION (OCQ)

OCQ150 What kind of business or industry is this?

(For example: TV and radio management, retail shoe store, state labor department, farm.)

(INTERVIEWER: USE ACTIVE PROBES AS NECESSARY TO GET A CLEAR PICTURE OF THE INDUSTRY IN WHICH THEY WORK. INCLUDE THE NAME OF THE BUSINESS, JOB OR INDUSTRY)

<1> ENTER RESPONSE FOLLOWED BY //

<d> DON'T KNOW
<r> REFUSED

**OCQ150**	**FMT_OCQ_TEXTCODE.**
**OCQ150_TEXT**	**$FMT_CHAR.**

OCQ160 Is this mainly manufacturing, wholesale trade, retail trade or something else?

<1> MANUFACTURING (MAKING A PRODUCT)
<2> WHOLESALE (SELLING TO BUSINESSES)
<3> RETAIL (SELLING TO CONSUMERS)
<4> SOMETHING ELSE (FOR EXAMPLE: EDUCATION, TRANSPORTATION, GOVERNMENT, HEALTHCARE, AGRICULTURE, MINING, INSURANCE, BANKING, ENTERTAINMENT, REAL ESTATE, SERVICES, ETC.)

<d> DON'T KNOW
<r> REFUSED

**OCQ160**	**FMT_OCQ160_225_.**

OCQ170 Looking at this card, which of these **best** describes this job or work situation?

(INTERVIEWER: HAND CARD)

<1> An employee of a **private** company, business, or individual for wages, salary or commission
<2> A **federal** government employee
<3> A **state** government employee
<4> A **local** government employee
<5> Self-employed in **own** business, professional practice or farm
<6> Working **without pay** in family business or farm

<d> DON'T KNOW
<r> REFUSED

**OCQ170**	**FMT_OCQ170_226_.**

For peer review only - http://bmjopen.bmj.com/site/about/guidelines.xhtml

OCCUPATION (OCQ)

OCQ175 On average, how many hours per week do you work at this job?

<1-168> HOURS PER WEEK

<d> DON'T KNOW
<r> REFUSED

OCQ175 FMT_NUMERIC.

OCQ180 At any time over the past year, have you worked in agriculture? For example farming, livestock production, commercial fishing, or forestry.

<1> YES
<2> NO

<d> DON'T KNOW
<r> REFUSED

OCQ180 FMT_YES_NO.

OCQ185 At any time over the past year, have you worked or volunteered in a setting that provides healthcare to patients?

(INTERVIEWER: HAND CARD)

For example: medical clinic, doctor's office, dentist's office, hospital, nursing home or some other health-care facility. This includes emergency responders and public safety personnel, part-time and unpaid work in a health care facility as well as professional nursing care provided in the home. This also includes non-health care professionals, such as administrative staff, who work in a health-care facility.

<1> YES
<2> NO **[Skip or go to OCQ14010pre]**

<d> DON'T KNOW
<r> REFUSED **[Skip or go to OCQ14010pre]**

OCQ185 FMT_YES_NO.

OCQ186 Did you provide direct patient care as part of your routine? By direct patient care, we mean physical or hands on contact with patients?

<1> YES
<2> NO

<d> DON'T KNOW

For peer review only - http://bmjopen.bmj.com/site/about/guidelines.xhtml

OCCUPATION (OCQ)

<r> REFUSED

OCQ186 FMT_YES_NO.

OCQ14010pre Many people shop, exercise, and run errands near where they work. To better understand the resources that might be available to you, we would now like to collect information about your job.

INTERVIEWER: HIT ENTER TO CONTINUE

OCQ14010 What is the address of your current place of employment?

<1> ENTER ADDRESS (goto STREET)
<99> WORKS FROM HOME **(SKIP TO PAQ200pre)**

OCQ14010_1 FMT_OCQ14010_1_.

<d> DON'T KNOW **(GO TO OCQ14020)**
<r> REFUSED **(GO TO OCQ14020)**

STREET ________________________

OCQ14010_A $FMT_CHAR.

CITY____________________________

OCQ14010_B $FMT_CHAR.

ZIPCODE ________

OCQ14010_C $FMT_CHAR.

OCQ14020 How many miles is your current place of employment from your home?

<0-300> MILES

<d> DON'T KNOW
<r> REFUSED

OCQ14020 FMT_NUMERIC.

For peer review only - http://bmjopen.bmj.com/site/about/guidelines.xhtml

Depression diagnostic and Severity Measure (PHQ)

PHQ001 Please indicate how much you have been bothered by these problems.

This section will take about 5 minutes.

Enter **1** to continue.

<1> CONTINUE

PHQ010 Over the **past 2** weeks, how often have you been bothered by any of the following problems:

Little interest or pleasure in doing things?

Would you say not at all, several days, more than half the days, or nearly every day?

Enter **1** for **not at all**, **2** for **several days**, **3** for **more than half the days**, or **4** for **nearly every day**.

<1> NOT AT ALL **PHQ010 FMT_PHQ_OFTEN.**

<2> SEVERAL DAYS
<3> MORE THAN HALF THE DAYS
<4> NEARLY EVERY DAY

<d> DON'T KNOW **<r> REFUSED**
<q> REPLAY QUESTION <h> REPLAY RESPONSES
<y> TURN QUESTION AUDIO OFF (SOUND IS NOW **ON**)
<s> TURN RESPONSE AUDIO OFF (SOUND IS NOW **ON**)

For peer review only

PHQ020 Over the **past 2** weeks, how often have you been bothered by any of the following problems:

Feeling down, depressed, or hopeless?

Would you say not at all, several days, more than half the days, or nearly every day?

Enter **1** for **not at all**, **2** for **several days**, **3** for **more than half the days**, or **4** for **nearly every day**.

<1> NOT AT ALL **PHQ020 FMT_PHQ_OFTEN.**
<2> SEVERAL DAYS
<3> MORE THAN HALF THE DAYS
<4> NEARLY EVERY DAY

<d> DON'T KNOW **<r> REFUSED**
<q> REPLAY QUESTION <h> REPLAY RESPONSES

For peer review only - http://bmjopen.bmj.com/site/about/guidelines.xhtml

Depression diagnostic and Severity Measure (PHQ)

<y> TURN QUESTION AUDIO OFF (SOUND IS NOW **ON**)
<s> TURN RESPONSE AUDIO OFF (SOUND IS NOW **ON**)

For peer review only

For peer review only - http://bmjopen.bmj.com/site/about/guidelines.xhtml

PTSD Checklist

Below is a list of problems and complaints that people sometimes have in response to stressful life experiences.

Please read each one below, and fill in the circle to indicate how much you have been bothered by the problem in the last month.

	Not at all	A little bit	Moderately	Quite a bit	Extremely
**1.** Repeated disturbing memories, thoughts, or images of a stressful experience from the past	○	○	○	○	○
**2.** Feeling very upset when something reminded you of a stressful experience from the past	○	○	○	○	○
**3.** Avoided activities or situations because they reminded you of a stressful experience from the past	○	○	○	○	○
**4.** Feeling distant or cut off from other people	○	○	○	○	○
**5.** Having trouble falling or staying asleep	○	○	○	○	○
**6.** Feeling irritable or having angry outbursts	○	○	○	○	○
**7.** Difficulty concentrating	○	○	○	○	○

For peer review only

PRESCRIPTION MEDICATIONS AND SELECT OTC DRUGS (RXQ)

RXQ032pre Now I'd like to gather information about any medication you might be taking.

INTERVIEWER: HIT ENTER TO CONTINUE

RXQ032 In the **past 30 days**, have you used a **prescription medicine**? Include only those products prescribed by a health professional such as a doctor, a nurse practitioner or a dentist.

RXQ032 FMT_YES_NO.

<1> YES
<2> NO **(Skip to RXQ296)**

<d> DON'T KNOW **(Skip to RXQ296)**
<r> REFUSED **(Skip to RXQ296)**

For peer review only

RXQ042 I'd like to see the CONTAINERS for **all** the prescription medicines that you used or took in the **past 30 days.**

INTERVIEWER: ENTER THE NAME OF EACH DRUG FROM THE PRESCRIPTION BOTTLE UNTIL NO MORE PRESCRIPTION MEDICINES, THEN HIT X TO EXIT QUESTION

<1> ENTER NAME(S)
<d> DON'T KNOW
<r> REFUSED

RXQ042@a___________________________________

RXQ042@b___________________________________

RXQ042@c ___________________________________

RXQ042@d ___________________________________

RXQ042@e___________________________________

RXQ042@f___________________________________

RXQ042@g___________________________________

RXQ042@h____________________________________

RXQ042@i ___________________________________

RXQ042@qq ARE THERE MORE PRESCRIPTION MEDICINES?

<1> YES (go to RXQ042@j) <2> NO

For peer review only - http://bmjopen.bmj.com/site/about/guidelines.xhtml

PRESCRIPTION MEDICATIONS AND SELECT OTC DRUGS (RXQ)

INTERVIEWER: ENTER THE NAME OF EACH DRUG FROM THE PRESCRIPTION BOTTLE UNTIL NO MORE PRESCRIPTION MEDICINES, THEN HIT X TO EXIT QUESTION

RXQ042p2@j_______________________________________

RXQ042p2@k_______________________________________

RXQ042p2@l _______________________________________

RXQ042p2@m _______________________________________

RXQ042p2@n _______________________________________

RXQ042p2@o_______________________________________

RXQ042p2@p_______________________________________

RXQ042p2@q _______________________________________

RXQ042p2@r_______________________________________

RXQ042p2@s _______________________________________

RXQ042p2@t _______________________________________

**RXQ042_A**	**$FMT_CHAR.**
**RXQ042_B**	**$FMT_CHAR.**
**RXQ042_C**	**$FMT_CHAR.**
**RXQ042_D**	**$FMT_CHAR.**
**RXQ042_E**	**$FMT_CHAR.**
**RXQ042_F**	**$FMT_CHAR.**
**RXQ042_G**	**$FMT_CHAR.**
**RXQ042_H**	**$FMT_CHAR.**
**RXQ042_I**	**$FMT_CHAR.**
**RXQ042_J**	**$FMT_CHAR.**
**RXQ042_K**	**$FMT_CHAR.**
**RXQ042_L**	**$FMT_CHAR.**
**RXQ042_M**	**$FMT_CHAR.**
**RXQ042_N**	**$FMT_CHAR.**
**RXQ042_O**	**$FMT_CHAR.**
**RXQ042_P**	**$FMT_CHAR.**
**RXQ042_Q**	**$FMT_CHAR.**
**RXQ042_R**	**$FMT_CHAR.**
**RXQ042_S**	**$FMT_CHAR.**
**RXQ042_T**	**$FMT_CHAR.**

RXQ231 Are there any **prescription medications** that you have used in the **past**

For peer review only - http://bmjopen.bmj.com/site/about/guidelines.xhtml

PRESCRIPTION MEDICATIONS AND SELECT OTC DRUGS (RXQ)

30 days for which you no longer have a prescription bottle or container? Again, these are products prescribed by a health professional such as a doctor, a nurse practitioner or a dentist.

RXQ231 FMT_YES_NO.

<1> YES
<2> NO **(Skip to RXQ294)**

<d> DON'T KNOW **(Skip to RXQ294)**
<r> REFUSED **(Skip to RXQ294)**

RXQ235 What is the name of each such drug?

INTERVIEWER: ENTER THE NAME OF EACH DRUG UNTIL NO MORE DRUGS, THEN HIT X TO EXIT QUESTION

<1> ENTER NAME(S)
<d> DON'T KNOW
<r> REFUSED

RXQ235@a ____________________________________

RXQ235@b ____________________________________

RXQ235@c ____________________________________

RXQ235@d ____________________________________

RXQ235@e ____________________________________

RXQ235@f ____________________________________

RXQ235@g ____________________________________

RXQ235@h ____________________________________

RXQ235@i ____________________________________

RXQ235@qq ARE THERE MORE PRESCRIPTION MEDICINES?

<1> YES (go to RXQ235@j) <2> NO

INTERVIEWER: ENTER THE NAME OF EACH DRUG UNTIL NO MORE DRUGS, THEN HIT X TO EXIT QUESTION

RXQ235p2@j ____________________________________

For peer review only - http://bmjopen.bmj.com/site/about/guidelines.xhtml

PRESCRIPTION MEDICATIONS AND SELECT OTC DRUGS (RXQ)

RXQ235p2@k ________________________________

RXQ235p2@l ________________________________

RXQ235p2@m ________________________________

RXQ235p2@n ________________________________

RXQ235p2@o ________________________________

RXQ235p2@p ________________________________

RXQ235p2@q ________________________________

RXQ235p2@r ________________________________

RXQ235p2@s ________________________________

RXQ235p2@t ________________________________

**RXQ235_A**	**$FMT_CHAR.**
**RXQ235_B**	**$FMT_CHAR.**
**RXQ235_C**	**$FMT_CHAR.**
**RXQ235_D**	**$FMT_CHAR.**
**RXQ235_E**	**$FMT_CHAR.**
**RXQ235_F**	**$FMT_CHAR.**
**RXQ235_G**	**$FMT_CHAR.**
**RXQ235_H**	**$FMT_CHAR.**
**RXQ235_I**	**$FMT_CHAR.**
**RXQ235_J**	**$FMT_CHAR.**
**RXQ235_K**	**$FMT_CHAR.**
**RXQ235_L**	**$FMT_CHAR.**
**RXQ235_M**	**$FMT_CHAR.**
**RXQ235_N**	**$FMT_CHAR.**
**RXQ235_O**	**$FMT_CHAR.**
**RXQ235_P**	**$FMT_CHAR.**
**RXQ235_Q**	**$FMT_CHAR.**
**RXQ235_R**	**$FMT_CHAR.**
**RXQ235_S**	**$FMT_CHAR.**
**RXQ235_T**	**$FMT_CHAR.**

RXQ294 Are there any other prescription medications that you used in the past 30 days?

**RXQ294**	**FMT_YES_NO.**

<1> YES
<2> NO

<d> DON'T KNOW
<r> REFUSED

For peer review only - http://bmjopen.bmj.com/site/about/guidelines.xhtml

PRESCRIPTION MEDICATIONS AND SELECT OTC DRUGS (RXQ)

[Loop back to RXQ235 as many times as needed.]

RXQ295 I have listed {TOTAL NUMBER} prescription medication(s) that you have taken in the **past 30 days**.

(**INTERVIEWER:** REVIEW TOTAL NUMBER OF PRESCRIBED MEDICATIONS AND THEIR NAMES WITH RESPONDENT)

(INTERVIEWER: USE PAGE UP/DOWN TO NAVIGATE THROUGH THE MEDICATIONS**).**

Is this correct?

RXQ295 FMT_YES_NO.

<1> YES
<2> NO (GO BACK TO ADD MEDICATION) **[goto RXQ042]**

RXQ296 In the last 30 days, have you taken any of the following types of over the counter, non-prescription drugs?

(INTERVIEWER: ENTER ALL THAT APPLY. HAND CARD)

<1> No
<2> Low dose aspirin to protect heart
<3> Drugs for pain/analgesics (including regular dose aspirin, Motrin, Tylenol, etc.)
<4> Allergy medications
<5> Drugs to help stop smoking, including nicotine gum
<6> Drugs for intestinal problems
<8> Drugs for cold and cough
<10> Drugs to help you lose or gain weight
<11> Vitamins or minerals (including calcium supplements)
<12> Other (Specify)

<d> DON'T KNOW
<r> REFUSED

**RXQ296_A**	**FMT_RXQ296_.**
**RXQ296_B**	**FMT_RXQ296_.**
**RXQ296_C**	**FMT_RXQ296_.**
**RXQ296_D**	**FMT_RXQ296_.**
**RXQ296_E**	**FMT_RXQ296_.**
**RXQ296_F**	**FMT_RXQ296_.**
**RXQ296_G**	**FMT_RXQ296_.**
**RXQ296_H**	**FMT_RXQ296_.**
**RXQ296_I**	**FMT_RXQ296_.**
**RXQ296_OTHER**	**$FMT_CHAR.**

For peer review only - http://bmjopen.bmj.com/site/about/guidelines.xhtml
For peer review only

PRESCRIPTION MEDICATIONS AND SELECT OTC DRUGS (RXQ)

RXQ300pre The following questions are about your exposure to vitamin D from supplements.

INTERVIEWER: HIT ENTER TO CONTINUE

RXQ300 In the **past 30 days**, have you used any multivitamins, vitamin D supplements with or without calcium, or cod liver oil?

<1> YES
<2> NO **(END QUESTIONNAIRE)**

<d> DON'T KNOW **(END QUESTIONNAIRE)**
<r> REFUSED **(END QUESTIONNAIRE)**

RXQ300 FMT_YES_NO.

RXQ301 I'd like to see the CONTAINERS for any multivitamins, vitamin D supplements with or without calcium, or cod liver oil that you took in the **past 30 days**.

INTERVIEWER: ENTER THE NAME OF EACH SUPPLEMENT FROM THE BOTTLE UNTIL NO MORE SUPPLEMENTS, THEN HIT X TO EXIT QUESTION

<1> ENTER NAME(S)
<d> DON'T KNOW
<r> REFUSED

RXQ301@a________________________________

RXQ301@b________________________________

RXQ301@c ________________________________

RXQ301@d ________________________________

RXQ301@e ________________________________

RXQ301@f ________________________________

RXQ301_A $FMT_CHAR.
RXQ301_B $FMT_CHAR.
RXQ301_C $FMT_CHAR.
RXQ301_D $FMT_CHAR.
RXQ301_E $FMT_CHAR.
RXQ301_F $FMT_CHAR.

RXQ302 Are there any multivitamin, **vitamin D supplements or bottles of cod liver oil** that you have used in the **past 30 days for which you no longer have a bottle or container**?

For peer review only - http://bmjopen.bmj.com/site/about/guidelines.xhtml

PRESCRIPTION MEDICATIONS AND SELECT OTC DRUGS (RXQ)

<1> YES **(GO TO RXQ 303)**
<2> NO **(SKIP TO RXQ304pre)**

<d> DON'T KNOW **(SKIP TO RXQ304pre)**
<r> REFUSED **(SKIP TO RXQ304pre)**

RXQ302 FMT_YES_NO.

RXQ303 What is the name of each such supplement?

INTERVIEWER: ENTER THE NAME OF EACH SUPPLEMENT UNTIL NO MORE SUPPLEMENTS, THEN HIT X TO EXIT QUESTION

<1> ENTER NAME(S)
<d> DON'T KNOW
<r> REFUSED

RXQ303@a ________________________________

RXQ303@b ________________________________

RXQ303@c ________________________________

RXQ303@d ________________________________

RXQ303@e ________________________________

RXQ303@f ________________________________

RXQ303_A $FMT_CHAR.
RXQ303_B $FMT_CHAR.
RXQ303_C $FMT_CHAR.
RXQ303_D $FMT_CHAR.
RXQ303_E $FMT_CHAR.
RXQ303_F $FMT_CHAR.

[if RXQ301@ ne <1> and RXQ303 ne <1> END SECTION]

RXQ304pre For these questions, please only think about the last 30 days. Please answer each question as best you can, and estimate if you are not sure.

INTERVIEWER: HIT ENTER TO CONTINUE

RXQ304_# In the last 30 days, on how many days per week have you taken [Fill drug name]? Would you say none or less than 1 day per week, 1 day per week, 2 days per week, 3 to 4 days per week, 5 to 6 days per week or every day?

(INTERVIEWER: HAND CARD)

For peer review only - http://bmjopen.bmj.com/site/about/guidelines.xhtml

PRESCRIPTION MEDICATIONS AND SELECT OTC DRUGS (RXQ)

<0> NONE OR LESS THAN 1 DAY PER WEEK
<1> 1 DAY PER WEEK
<2> 2 DAYS PER WEEK
<3> 3-4 DAYS PER WEEK
<5> 5-6 DAYS PER WEEK
<7> EVERY DAY

<d> DON'T KNOW
<r> REFUSED

**RXQ304_A**	**FMT_RXQ304_.**
**RXQ304_B**	**FMT_RXQ304_.**
**RXQ304_C**	**FMT_RXQ304_.**
**RXQ304_D**	**FMT_RXQ304_.**
**RXQ304_E**	**FMT_RXQ304_.**
**RXQ304_F**	**FMT_RXQ304_.**
**RXQ304_G**	**FMT_RXQ304_.**
**RXQ304_H**	**FMT_RXQ304_.**
**RXQ304_I**	**FMT_RXQ304_.**
**RXQ304_J**	**FMT_RXQ304_.**
**RXQ304_K**	**FMT_RXQ304_.**
**RXQ304_L**	**FMT_RXQ304_.**

RXQ305_# **INTERVIEWER: ENTER THE SERVING SIZE (NUMBER OF PILLS OR CHEWS OR VOLUME OF LIQUID OR AMOUNT OF POWDER) ON THE SUPPLEMENT LABEL OF [fill drug name].**

<0.00 – 30.00> NUMBER (SERVING SIZE)

<d> DON'T KNOW
<r> REFUSED

**RXQ305_A1**	**FMT_NUMERIC.**
**RXQ305_B1**	**FMT_NUMERIC.**
**RXQ305_C1**	**FMT_NUMERIC.**
**RXQ305_D1**	**FMT_NUMERIC.**
**RXQ305_E1**	**FMT_NUMERIC.**
**RXQ305_F1**	**FMT_NUMERIC.**
**RXQ305_G1**	**FMT_NUMERIC.**
**RXQ305_H1**	**FMT_NUMERIC.**
**RXQ305_I1**	**FMT_NUMERIC.**
**RXQ305_J1**	**FMT_NUMERIC.**
**RXQ305_K1**	**FMT_NUMERIC.**
**RXQ305_L1**	**FMT_NUMERIC.**

THE UNIT FOR THE SERVING OF [fill drug name]:

<1> PILLS OR CHEWS
<2> VOLUME IN MLS OR CC
<3> VOLUME IN OUNCES (OZ)
<4> TEASPOON (TSP)
<5> TABLESPOON (TBSP)

For peer review only - http://bmjopen.bmj.com/site/about/guidelines.xhtml

PRESCRIPTION MEDICATIONS AND SELECT OTC DRUGS (RXQ)

<6> SCOOPS (POWDER)

<d> DON'T KNOW
<r> REFUSED

RXQ305_A2	FMT_RXQ305_.
RXQ305_B2	FMT_RXQ305_.
RXQ305_C2	FMT_RXQ305_.
RXQ305_D2	FMT_RXQ305_.
RXQ305_E2	FMT_RXQ305_.
RXQ305_F2	FMT_RXQ305_.
RXQ305_G2	FMT_RXQ305_.
RXQ305_H2	FMT_RXQ305_.
RXQ305_I2	FMT_RXQ305_.
RXQ305_J2	FMT_RXQ305_.
RXQ305_K2	FMT_RXQ305_.
RXQ305_L2	FMT_RXQ305_.

RXQ306_# **INTERVIEWER: ENTER THE IU OF VITAMIN D PER SERVING ON THE SUPPLEMENT LABEL OF [Fill drug name]**

(IF NONE, ENTER 0):

<0-50,000> IU

<d> DON'T KNOW
<r> REFUSED

RXQ306_A	FMT_NUMERIC.
RXQ306_B	FMT_NUMERIC.
RXQ306_C	FMT_NUMERIC.
RXQ306_D	FMT_NUMERIC.
RXQ306_E	FMT_NUMERIC.
RXQ306_F	FMT_NUMERIC.
RXQ306_G	FMT_NUMERIC.
RXQ306_H	FMT_NUMERIC.
RXQ306_I	FMT_NUMERIC.
RXQ306_J	FMT_NUMERIC.
RXQ306_K	FMT_NUMERIC.
RXQ306_L	FMT_NUMERIC.

RXQ307 On the days you took the {fill drug name}, how many {fill unit} did you take?

<1-30.00> {fill unit}

<d> DON'T KNOW
<r> REFUSED

RXQ307_A	FMT_NUMERIC.
RXQ307_B	FMT_NUMERIC.
RXQ307_C	FMT_NUMERIC.

For peer review only - http://bmjopen.bmj.com/site/about/guidelines.xhtml

PRESCRIPTION MEDICATIONS AND SELECT OTC DRUGS (RXQ)

**RXQ307_D**	**FMT_NUMERIC.**
**RXQ307_E**	**FMT_NUMERIC.**
**RXQ307_F**	**FMT_NUMERIC.**
**RXQ307_G**	**FMT_NUMERIC.**
**RXQ307_H**	**FMT_NUMERIC.**
**RXQ307_I**	**FMT_NUMERIC.**
**RXQ307_J**	**FMT_NUMERIC.**
**RXQ307_K**	**FMT_NUMERIC.**
**RXQ307_L**	**FMT_NUMERIC.**

[REPEAT RXQ304 – RXQ307 for each drug listed at RXQ301 AND RXQ303]

For peer review only

For peer review only - http://bmjopen.bmj.com/site/about/guidelines.xhtml

Smoking and Other Tobacco Products

The next questions are about your history of using tobacco products.

1. Have you smoked 100 or more cigarettes in your entire life? SMQ020_R2 FMT_YES_NO.

○ Yes ○ No → **Go to question 11, page 21**

2. How old were you when you started smoking cigarettes regularly? SMQ030_R2. FMT_NUMERIC.

Enter age when you started smoking: [ ][ ]

3. Do you smoke cigarettes now?

○ Yes ○ No → **Go to question 9, page 21** SMQ040_R2 FMT_YES_NO.

4. Is your usual cigarette brand menthol or non-menthol?

○ Menthol ○ Non-menthol SMQ045_R2 FMT_SMQ045_.

5. On average, when you smoked during the past 30 days, about how many cigarettes did you smoke per day? *If you smoked less than 1 cigarette per day, enter 1 (1 pack = 20 cigarettes).*

Enter number of cigarettes per day: [ ][ ] SMQ050_R2 FMT_NUMERIC.

6. For about how many years have you smoked this amount?

Enter number of years: [ ][ ] SMQ060_R2 FMT_NUMERIC.

7. Would you like to completely quit smoking cigarettes?

○ Yes ○ No SMQ064_R2 FMT_YES_NO.

8. During the past 12 months, has a doctor or other health professional talked to you about your smoking?

○ Yes ○ No SMQ065_R2 FMT_YES_NO.

Go to question 11, page 21 →

For peer review only - http://bmjopen.bmj.com/site/about/guidelines.xhtml

Please answer questions 9 and 10 *only if* you answered NO to question 3. Otherwise, begin with question 11.

9. How old were you when you stopped smoking?

Enter the age you stopped smoking: [|] SMQ120_R2 FMT_NUMERIC.

10. On average, over the entire time you smoked, about how many cigarettes did you smoke per day? *If you smoked less than 1 cigarette per day, enter 1 (1 pack = 20 cigarettes).*

Enter number of cigarettes daily: [|] SMQ140_R2 FMT_NUMERIC.

<u>**Everyone should answer the following questions.**</u>

Now think about a typical week.

11. How many hours per week are you currently exposed to cigarette smoke in social settings outside your own home? *(This would include time spent with friends or relatives who smoke, time spent in restaurants or taverns, or other social affairs where people are smoking.)*

Enter hours per week: [|] SMQ230_R2 FMT_NUMERIC.

12. Do any people currently smoke cigarettes inside your home?

○ Yes ○ No SMQ185_R2 FMT_YES_NO.

The next questions are about your use of tobacco products other than cigarettes (such as cigars, pipes, water pipes, hookahs, very small cigars that look like cigarettes, bidis, or cigarillos).

13. Have you <u>ever</u> smoked tobacco products other than cigarettes? *(Do not include electronic cigarettes or e-cigarettes.)*

○ Yes ○ No SMQ233_R2 FMT_YES_NO.

14. Do you <u>now</u> smoke tobacco products other than cigarettes every day, some days, or not at all?

○ Every day
○ Some days
○ Not at all

SMQ240_R2 FMT_SMQFREQ.

For peer review only

15. Have you ever used any smokeless tobacco products, such as chewing tobacco, snuff, snus, dip, orbs, sticks, or strips?

◯ Yes ◯ No SMQ250_R2 FMT_YES_NO.

16. Do you now use any smokeless tobacco products, such as chewing tobacco, snuff, snus, dip, orbs, sticks, or strips?

◯ Every day
◯ Some days
◯ Not at all

SMQ260_R2 FMT_SMQFREQ.

17. In the past 12 months, how often has tobacco smoke entered your living space from somewhere else in or around your home (for example, from a neighbor)?

◯ Most of the time
◯ Often
◯ Sometimes
◯ Rarely
◯ Never

SMQ270_R2 FMT_SMQ270_.

18. During the past 7 days, on how many days did you ride in a vehicle where someone other than you was smoking tobacco? *If none, then enter 0.*

Enter the number of days: ☐ SMQ280_R2 FMT_NUMERIC.

◯ Don't know

19. Not counting motorcycles, in the vehicles that you or your family members who live with you own or lease, is smoking...

◯ Always allowed in all vehicles
◯ Sometimes allowed in at least one vehicle
◯ Never allowed in any vehicle
◯ I/we don't own or lease a vehicle

SMQ290_R2 FMT_SMQ290_.

For peer review only - http://bmjopen.bmj.com/site/about/guidelines.xhtml

For peer review only

20. Have you ever used electronic cigarettes or e-cigarettes?
An electronic cigarette, or e-cigarette, is a new product that looks like a regular cigarette, but is not lighted like a cigarette. It runs on a battery and has a smoke-like vapor that is produced electronically. The vapor contains nicotine, but the e-cigarette does not contain or burn any tobacco.

○ Yes ○ No SMQ300_R2 FMT_YES_NO.

21. Do you now use electronic cigarettes (e-cigarettes) every day, some days, or not at all?

○ Every day SMQ310_R2 FMT_SMQFREQ.
○ Some days
○ Not at all

22. Do you think secondhand smoke is...

○ Very harmful to one's health
○ Somewhat harmful to one's health SMQ320_R2 FMT_SMQ320_.
○ Not very harmful to one's health
○ Not harmful to one's health

For peer review only

STROBE Statement—checklist of items that should be included in reports of observational studies

	Item No	Recommendation
**Title and abstract**	1	(*a*) Indicate the study's design with a commonly used term in the title or the abstract
		(*b*) Provide in the abstract an informative and balanced summary of what was done and what was found
**Introduction**		
Background/rationale	2	Explain the scientific background and rationale for the investigation being reported
Objectives	3	State specific objectives, including any prespecified hypotheses
**Methods**		
Study design	4	Present key elements of study design early in the paper
Setting	5	Describe the setting, locations, and relevant dates, including periods of recruitment, exposure, follow-up, and data collection
Participants	6	(*a*) *Cohort study*—Give the eligibility criteria, and the sources and methods of selection of participants. Describe methods of follow-up<br>*Case-control study*—Give the eligibility criteria, and the sources and methods of case ascertainment and control selection. Give the rationale for the choice of cases and controls<br>*Cross-sectional study*—Give the eligibility criteria, and the sources and methods of selection of participants
		(*b*) *Cohort study*—For matched studies, give matching criteria and number of exposed and unexposed<br>*Case-control study*—For matched studies, give matching criteria and the number of controls per case
Variables	7	Clearly define all outcomes, exposures, predictors, potential confounders, and effect modifiers. Give diagnostic criteria, if applicable
Data sources/ measurement	8*	For each variable of interest, give sources of data and details of methods of assessment (measurement). Describe comparability of assessment methods if there is more than one group
Bias	9	Describe any efforts to address potential sources of bias
Study size	10	Explain how the study size was arrived at
Quantitative variables	11	Explain how quantitative variables were handled in the analyses. If applicable, describe which groupings were chosen and why
Statistical methods	12	(*a*) Describe all statistical methods, including those used to control for confounding
		(*b*) Describe any methods used to examine subgroups and interactions
		(*c*) Explain how missing data were addressed
		(*d*) *Cohort study*—If applicable, explain how loss to follow-up was addressed<br>*Case-control study*—If applicable, explain how matching of cases and controls was addressed<br>*Cross-sectional study*—If applicable, describe analytical methods taking account of sampling strategy
		(*e*) Describe any sensitivity analyses

Continued on next page

For peer review only

**Discussion**		
Key results	18	Summarise key results with reference to study objectives
Limitations	19	Discuss limitations of the study, taking into account sources of potential bias or imprecision. Discuss both direction and magnitude of any potential bias
Interpretation	20	Give a cautious overall interpretation of results considering objectives, limitations, multiplicity of analyses, results from similar studies, and other relevant evidence
Generalisability	21	Discuss the generalisability (external validity) of the study results
**Other information**		
Funding	22	Give the source of funding and the role of the funders for the present study and, if applicable, for the original study on which the present article is based

*Give information separately for cases and controls in case-control studies and, if applicable, for exposed and unexposed groups in cohort and cross-sectional studies.

Note: An Explanation and Elaboration article discusses each checklist item and gives methodological background and published examples of transparent reporting. The STROBE checklist is best used in conjunction with this article (freely available on the Web sites of PLoS Medicine at http://www.plosmedicine.org/, Annals of Internal Medicine at http://www.annals.org/, and Epidemiology at http://www.epidem.com/). Information on the STROBE Initiative is available at www.strobe-statement.org.

For peer review only
